# Rescuing Cancer Immunity by Plasma Exchange in Metastatic Melanoma (ReCIPE-M1): protocol for a single-institution, open-label safety trial of plasma exchange to clear sPD-L1 for immunotherapy

**DOI:** 10.1136/bmjopen-2021-050112

**Published:** 2022-05-11

**Authors:** Tara M Davidson, Nathan Foster, Fabrice Lucien, Svetomir Markovic, Haidong Dong, Jeffrey L Winters, Sean S Park, Jacob J Orme

**Affiliations:** 1 Department of Internal Medicine, Mayo Clinic, Rochester, Minnesota, USA; 2 Department of Quantitative Health Sciences, Mayo Clinic, Rochester, Minnesota, USA; 3 Department of Urology, Mayo Clinic, Rochester, Minnesota, USA; 4 Division of Medical Oncology, Mayo Clinic, Rochester, Minnesota, USA; 5 Department of Pathology and Laboratory Medicine, Mayo Clinic, Rochester, Minnesota, USA; 6 Radiation Oncology, Mayo Clinic, Rochester, Minnesota, USA

**Keywords:** ONCOLOGY, IMMUNOLOGY, Radiobiology, Prostate disease

## Abstract

**Background:**

Patients with metastatic melanoma rely on PD-(L)1 immunotherapy, but only one-third of patients experience treatment response and all initial responders eventually develop resistance. Tumour-derived extracellular vesicles expressing Programmed death ligand 1 (evPD-L1) and soluble Programmed death ligand 1 (sPD-L1) in peripheral blood of patients with melanoma limit PD-(L)1 immunotherapy and correlate with poor survival. Therapeutic plasma exchange (TPE) removes immunosuppressive evPD-L1 and sPD-L1. We hypothesise that TPE may rescue and restore antimelanoma immunity.

**Methods:**

In this two-arm study, 60 patients with metastatic melanoma progressing on checkpoint inhibition will be accrued. All patients will undergo radiotherapy on days 1–5 (at least one measurable lesion will not be irradiated) and ongoing checkpoint inhibition on day 8 and every 2–3 weeks per standard of care. Patients with baseline sPD-L1 level of ≥1.7 ng/mL and adequate clinical capacity will be enrolled in the TPE intervention arm and will undergo TPE on days 5–7, in addition to standard of care radiotherapy and immunotherapy. Other patients will remain in the standard of care arm.

The primary endpoint of the study is to evaluate safety. Secondary endpoints include kinetics of sPD-L1 and evPD-L1 and clinical response by RECIST (Response Evaluation Criteria in Solid Tumors) criteria. Study registered at ClinicalTrials.gov (NCT04581382).

**Ethics and dissemination:**

This trial has been approved by the Mayo Clinic Institutional Review Board. It will assess the safety and feasibility of TPE in improving outcomes for PD-(L)1 inhibitor immunotherapy in melanoma. Data will be maintained on a secure database with deidentified patient information. Data will be shared on publication in a peer-reviewed journal without the aid of professional writers. If successful, this trial will lay the ground for phase II studies that will include cancer treated with PD-(L)1 inhibitors which may benefit from TPE such as renal, bladder and lung cancers.

**Trial registration number:**

NCT04581382.

**Strengths and limitations of this study**

- Screens 60 patients with metastatic melanoma progressing despite checkpoint inhibition.
- Evaluates the safety of therapeutic plasma exchange (TPE) and kinetics of immunomodulatory molecules removed by TPE in vivo in melanoma.
- Involves no randomisation to intervention versus control arm in this early study.

## Introduction

The first immunotherapy with interferon was approved by the Food and Drug Administration in 1986.^1^ Since then, leveraging the body’s own immune system to target and kill malignant cells has become the fastest-growing therapeutic approach to cancer worldwide.^2^ While early immunotherapies focused on providing immunostimulatory cytokines (eg, pegilodecakin), more recent approaches have leveraged vaccination (eg, MAGE-A3), transplant of primed antitumour CD8+ T cells (ie, CAR [chimeric antigen receptor] T therapy) and inhibiting immune checkpoint signals (eg, programmed death 1 (PD-1) and programmed death ligand 1 (PD-L1) inhibitors like pembrolizumab, nivolumab and atezolizumab).^3^

The latter approach with PD-(L)1 inhibitors has shown spectacular results in many indications, leading to first-line approvals of checkpoint inhibitor immunotherapy in renal cell carcinoma, non-small cell lung cancer, melanoma and others.^4–6^ Unfortunately, even in these highly promising settings, less than half of the patients have objective response to PD-(L)1 inhibitor therapy alone.^7^ For instance, only about one in three melanomas respond to PD-(L)1 inhibitor pembrolizumab.^6^ Intensive research is focused on identifying causes for intrinsic resistance to PD-(L)1 inhibitors.

PD-L1 is classically thought to be a membrane-bound ligand. Recently, however, three additional functional forms of *extracellular* PD-L1 have been identified. Soluble PD-L1 can be produced by transcribed PD-L1 splice variants, of which four variants have been identified in melanoma.^8 9^ Metalloproteases ADAM10 and ADAM17 can also cleave the PD-L1 ectodomain from tumour cells and extracellular vesicles (EVs) to form soluble programmed death ligand 1 (sPD-L1).^10^ Lastly, tumour cells release extracellular vesicles expressing programmed death ligand 1 (evPD-L1).^11^

sPD-L1 levels are significantly higher in patients with metastatic melanoma at baseline compared with healthy volunteers, and elevated levels predict inferior outcomes.^8 10 12^ This implies that the same inflammatory/immune response that is known to play a role in PD-L1 expression also increases the rate of extracellular PD-L1 production. In one study, PD-L1 cell surface expression paralleled sPD-L1 secretion in response to cytokines in melanoma cells.^8^ Some cancer cells secrete a majority of their PD-L1 on exosomes, even with only minimal amounts of cellular PD-L1 found.^9 11^ The leading hypothesis is that the tumour microenvironment, including non-neoplastic cells, may also be contributing to the production of sPD-L1 and evPD-L1. Moreover, although radiotherapy has been proposed as a method to induce antitumour immunity, studies have also shown that radiation increases the production of extracellular PD-L1 through inflammatory cytokines and other mechanisms.^13 14^

**Figure 1:**
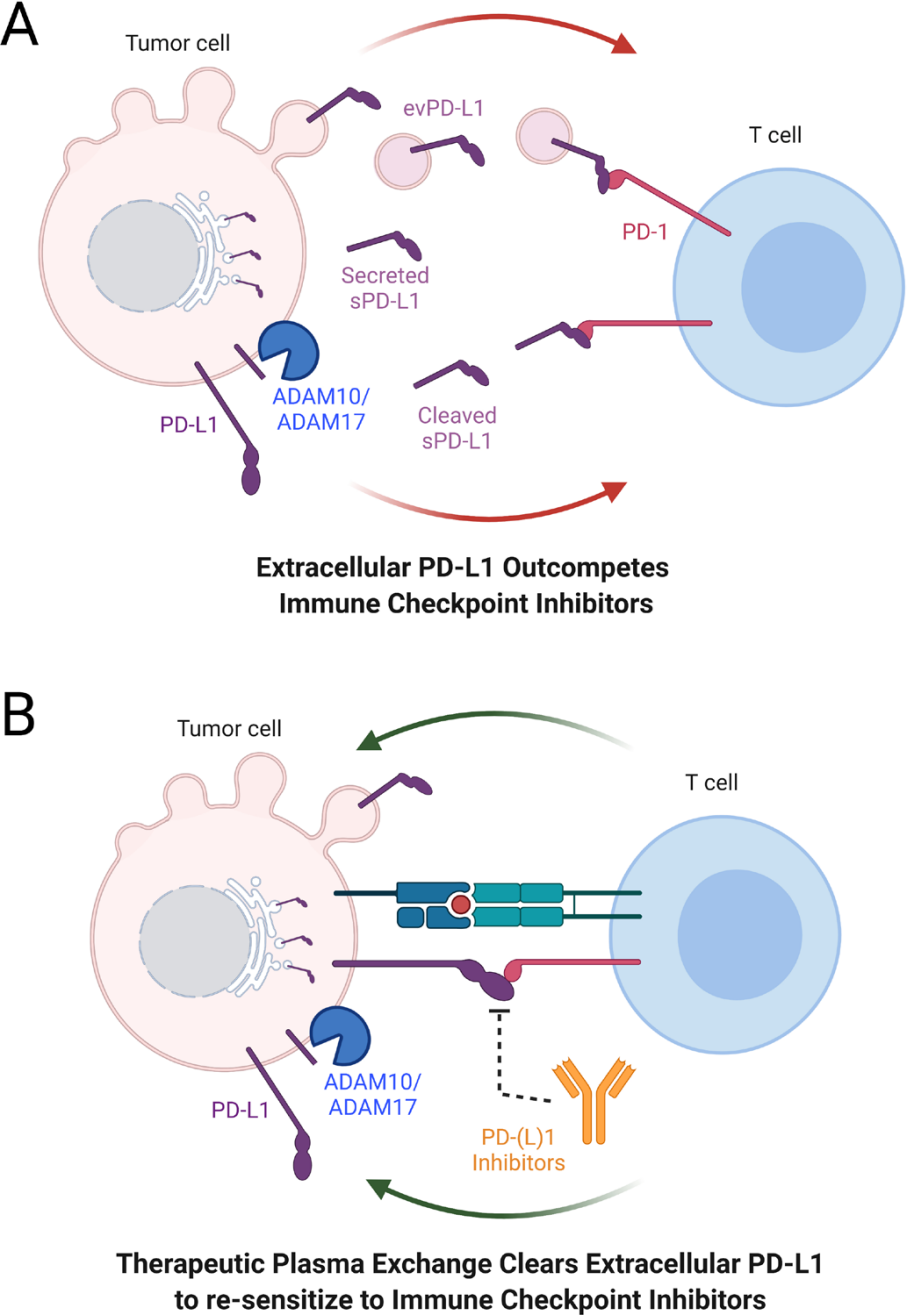
Extracellular PD-L1 outcompetes ICIs to block antitumour immunity. (A) While PD-(L)1 inhibitors prevent tumour cell downregulation of T cells, tumour cells secrete evPD-L1 and sPD-L1 (either cleaved by ADAM10/ADAM17 or as splice variants) that outcompete PD-(L)1 inhibitors and downregulate systemic anticancer immunity. (B) Therapeutic plasma exchange clears extracellular PD-L1 and may resensitise tumours to ICI therapy. evPD-L1, extracellular vesicles expressing programmed death ligand 1; ICI, immune checkpoint inhibitor; PD-1, programmed death 1; PD-L1, programmed death ligand 1; sPD-L1, soluble programmed death ligand 1.

While the roles of these extracellular PD-L1 forms continue to be elucidated, the results of sPD-L1 and evPD-L1 signalling are clear from both in vitro and in vivo studies: sPD-L1 and evPD-L1 outcompete PD-(L)1 inhibitors, initiate apoptosis in CD8+ T cells and cause systemic immunosuppression by limiting the ability of healthy peripheral blood mononuclear cells to kill tumour cells (figure 1A).^9 10 15^ Studies in multiple malignancies find a negative relationship between high sPD-L1 and outcomes.^16–27^ In melanoma, Zhou *et al* showed that high pretreatment levels of sPD-L1 were associated with increased likelihood of progressive disease in patients with melanoma treated by CTLA-4 or PD-1 blockade.^8^

We recently explored the potential of therapeutic plasma exchange (TPE) in removing sPD-L1 and evPD-L1.^28^ In brief, TPE is a procedure for passing blood through an apheresis machine to separate plasma from cellular components and is commonly used clinically for removal of deleterious antibodies.^29^ This has been described as a method to ‘clean’ or ‘flush out‘ the blood. Plasma containing the offending substance is replaced in this procedure with up to 30% crystalloid in the form of normal saline and either donor plasma or 5% human albumin derived from plasma donation. In our previous report, each session of TPE reduced sPD-L1 and evPD-L1 by 70.8% and 73.1%, respectively.^28^ This represents the first clinical intervention to remove either of these substances from circulation in vivo. It is hypothesised that the removal of these extracellular forms of PD-L1 may resensitise tumours to immune checkpoint inhibitor treatment (figure 1B).

Despite these important discoveries, several crucial questions remain unanswered. First, while TPE is commonly prescribed in the outpatient setting, it has not been studied specifically in cancer treated with immunotherapy. Second, the kinetics of sPD-L1 and evPD-L1 in patients with melanoma have not previously been determined. Pilot studies to establish the safety profile and durability of TPE-mediated changes in melanoma are warranted.

## Methods

### Overview of study design

This feasibility trial will assess (1) the safety of TPE through adverse events monitoring at 1 and 6 months post therapy (primary outcome); (2) the kinetics of evPD-L1, sPD-L1 and related immunosuppressive metabolites immediately and 2–3 weeks after TPE,; and (3) the relative response of patients to combined therapy through response rate, overall survival and progression-free survival.

### Recruitment, screening procedures and treatment arms

**Figure 2:**
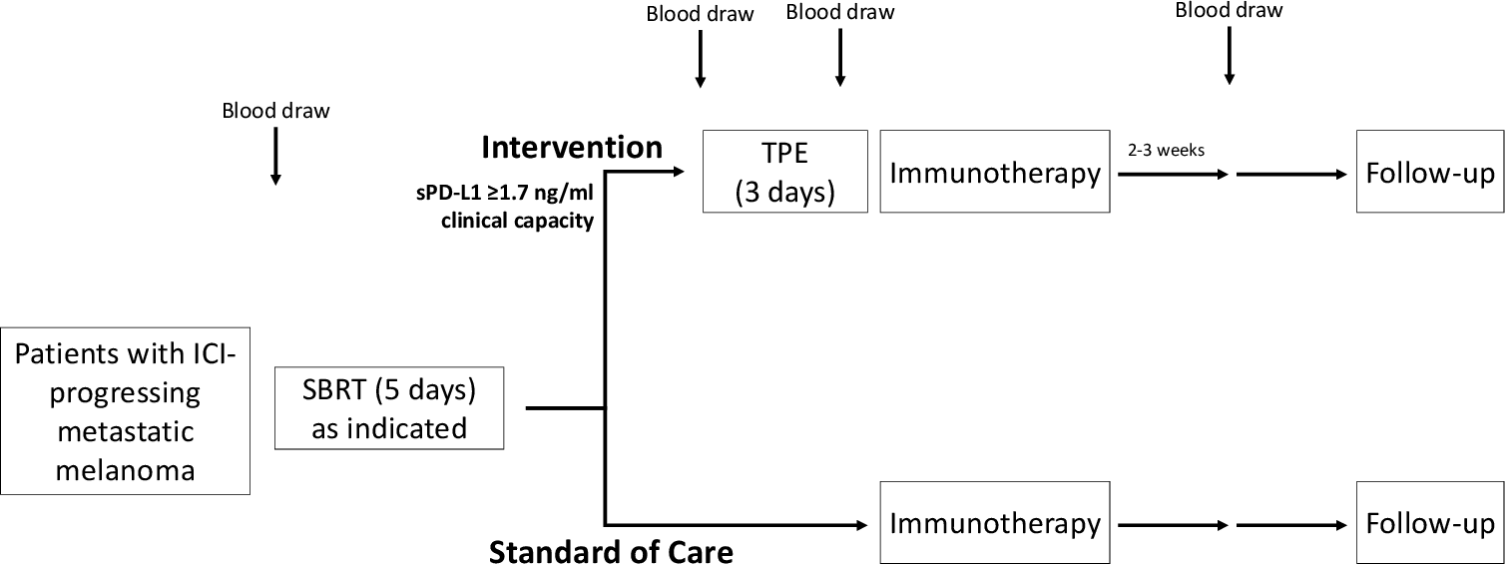
Study design in two arms. In brief, eligible patients undergo a registration blood draw and vascular access evaluation to determine the appropriate study arm. Patients with elevated baseline sPD-L1 (≥1.7 ng/mL) will enter the intervention arm if there is adequate clinical capacity. Other patients will enter the standard of care arm. ICI, immune checkpoint inhibitor; SBRT, stereotactic body radiation therapy; sPD-L1, soluble programmed death ligand 1; TPE, therapeutic plasma exchange.

Patients with metastatic melanoma progressing on standard of care on any PD-(L)1 inhibitor are eligible for this study (table 1 and figure 2). Potentially eligible patients will be approached by the clinical trial coordinator and will provide informed consent prior to a baseline screening blood draw. Sixty patients will be screened from December 2020 t0 October 2023 with an accrual goal of 20 patients to the TPE Intervention Arm. Inclusion criteria comprise age (18 or older), histologically confirmed melanoma, measurable disease per RECIST criteria, ECOG (Eastern Cooperative Oncology Group) status of 3 or better, sPD-L1 level 1.7 ng/mL or greater, and feasible vascular access. Exclusion criteria include pregnancy or nursing. Also excluded are those who consume biotin supplementation as it interferes with accurate measurement of sPD-L1.

**Table 1:** Study inclusion and exclusion criteria

Inclusion Criteria	Exclusion Criteria	Arm assignments	
≥18 years	Biotin supplement use	Intervention arm	sPD-L1 ≥1.7 ng/mL and available clinical capacity
Histological melanoma progressing despite immune checkpoint inhibitor	Pregnant or nursing		
Measurable disease		Standard of care arm	sPD-L1 <1.7 ng/mL or unavailable clinical capacity
ECOG ≤3			
Ability to consent			

Eligible patients for the study will be consenting adults with histologically confirmed melanoma receiving PD-(L)1 immunotherapy who are referred for stereotactic body radiation therapy. Patients who are receiving biotin supplements, pregnant or nursing are excluded. Patients with elevated sPD-L1 on registration will enter the intervention arm where clinical capacity is sufficient. Patients with low sPD-L1 or who enter the trial when clinical capacity is not available will enter the standard of care arm.

sPD-L1, soluble programmed death ligand 1.

Enrolled patients with sPD-L1 level at or above 1.7 ng/mL and able to undergo plasma exchange will be enrolled in the Intervention Arm. Other patients will be enrolled in the standard of care arm. All data will be collected in a deidentified fashion in a password-protected database.

### Study interventions

All patients will receive treatment with any PD-(L)1 inhibitor per clinician preference, including but not limited to the continuation of the existing checkpoint inhibitor, escalation to ipilimumab and nivolumab, or switching to an alternative checkpoint inhibitor. Other concomitant therapies will be allowed on the trial. Additional treatment comprises radiotherapy on days 1–5 and PD-(L)1 inhibitor treatment on day 8 and every 2–3 weeks thereafter as clinically indicated. Preferred targets for radiotherapy will be symptomatic lesions or lesions that can cause pain, fracture or other symptoms in the near future. Patients in the intervention arm, in addition to the aforementioned, will undergo serial blood draws prior to the first TPE session, after the last TPE session and just prior to the next dose of immunotherapy. We will assess the kinetics of sPD-L1 and evPD-L1 at these timepoints.

TPE will be performed using centrifugation-based cell separators, either the Fenwal Amicus (Fresenius KABI USA LLC, Lake Zurich, Illinois, USA) or the Spectra Optia (Terumo BCT, Lakewood, Colorado, USA). For each patient, a single plasma volume will be exchanged using either peripheral intravenous (preferred) or central lines for vascular access if a central line is present. Due to the possibility of sPD-L1 or PD-L1-positive EVs present in donor plasma, donor plasma (ie, fresh frozen plasma) will not be used as a replacement fluid. Rather, 5% normal serum human albumin with up to 500 mL of normal saline will be used as the replacement. The volume used is determined by the device based on patient gender, height, weight and haematocrit. Anticoagulation will consist of either 500 mL of acid citrate dextrose solution A (ACD-A) or 500 mL of ACD-A with 5000 units of unfractionated heparin. Anticoagulant to blood ratios will be 1:13 when ACD-A is used and 1:26 when ACD-A/heparin is used. Patients will not receive routine electrolyte replacement, but 10 mL of 10% calcium gluconate will be administered by slow intravenous push for signs and symptoms of hypocalcaemia related to the ACD-A anticoagulant.

Toxicity assessment using Common Terminology Criteria for Adverse Events (CTCAE) V.5.0 will be assessed within 7 days prior to registration as baseline in addition to daily during TPE and 2 weeks post first dose of immunotherapy if receiving nivolumab or 3 weeks post first dose of immunotherapy if receiving pembrolizumab as well as 3 months post TPE. Safety will also be assessed during any clinically indicated follow-up until progressive disease, death or up to 2 years after registration.

Imaging will be obtained as standard of care and is not stipulated by the study. CT, positron emission tomography and/or MRI scans can be used as clinically indicated. Imaging to assess tumour response will be based on RECIST/PERCIST (positron emission tomography response criteria in solid tumors) criteria V.1.1 until progression, death or up to 2 years after registration. At least one metastatic lesion will not be radiated to assess response per RECIST and/or PERCIST criteria for those in the intervention arm. All oligoprogressive lesions will be radiated for patients in the standard of care arm while on standard-of-care immunotherapy. As patients who progress on immunotherapy are enrolled in this trial, partial or complete response of >20% will be considered as a positive signal to proceed to a phase II trial. Study materials and procedures were approved by the Mayo Clinic Institutional Review Board (IRB) (approval number 19–008055).

### Measuring sPD-L1 and evPD-L1 levels

For measurements of sPD-L1, ELISA will be performed as previously published.^18^ Both secreted splice variant and shed sPD-L1 are reliably detected by this ELISA. In brief, paired mouse IgG2 monoclonal antibody clones H1A and B11 against extracellular human PD-L1 will be used in a capture-detection plate assay using a standard biotinylated antibody and streptavidin–HRP (Horseradish peroxidase) detection method. This assay is specific for sPD-L1 and does not exhibit cross reactivity to other B7-H homologues nor to evPD-L1. Concentrations are determined by optical density measurements along a known standard curve of recombinant human PD-L1. ELISAs are performed by technicians blinded to the identity of the samples.

For measurements of evPD-L1, flow cytometry will be used as previously published.^30^ In brief, plasma samples are centrifuged twice at 2000 *g* to deplete platelets. Resultant platelet-free plasma is analysed using an A60-Micro Plus Nanoscale Flow Cytometer (Apogee FlowSystems) gating for mid-intensity light angle light scatter and markers of interest. Anti-PD-L1 (atezolizumab, Genentech) antibodies are conjugated to fluorophores (Alexa-647, PE phycoerythrin and Alexa-488; Life Technologies) and titrated prior to use. Nanoscale flow cytometer calibration is performed using a standard reference bead mix as previously published. Flow cytometry is performed by technicians blinded to the identity of the samples. Wilcoxon signed-rank test will be used to assess changes in sPD-L1 and evPD-L1 levels over time across the different timepoints of interest.

### Endpoints

The primary endpoint of this trial is to assess the safety of TPE in patients with melanoma receiving PD-(L)1 immunotherapy. Adverse events will be collected according to CTCAE V.5.0, and the protocol dictates that if 3 of the first 10 patients experiences grade 4 or greater adverse events, the study will be halted. The secondary endpoints of this trial are (1) the kinetics of sPD-L1 and evPD-L1 removal in patients with melanoma undergoing PD-(L)1 immunotherapy, (2) overall response rate as a proportion of patients with a tumour response of PR (partial response) or CR (complete response) at least 4 weeks apart, and (3) progression-free and overall survival.

### Statistical methods

For this correlative analysis, we will determine the effects of plasma exchange on immune cell function, observe the kinetics of EVs after plasma exchange in patients with melanoma, and associate the kinetics with clinical outcome data (RR - response rate, OS - overall survival, and PFS - progression-free survival). Associations of categorical data will be assessed using Fisher exact tests. Associations of continuous data with binary data will be assessed using standard Wilcoxon rank-sum tests. Assessment of the change in continuous data over time will be done using the Wilcoxon signed-rank test. Kaplan-Meier methods and the log-rank test will be used for time-to-event data. This translational study is considered exploratory and hypothesis generating due to the small proposed sample size for this study.

### Data collection schedule

The principal investigator and study statistician will review the study at least monthly to identify accrual, adverse events and any endpoint problems that might be developing. The trial is monitored continually by the study team who are notified of every grade 4 or 5 event in real time.

### Patient and public involvement

No patient was involved.

## Ethics and dissemination

We hypothesise that TPE will reliably and safely reduce the levels of sPD-L1 and evPD-L1 in our study patients. We hypothesise that this will alleviate the immunosuppressive microenvironment caused by extracellular PD-L1 as well as minimise any competition for PD-(L)1 inhibitors prior to study patients receiving their dose of PD-(L)1 inhibitor therapy. We hope to see improved response to these agents through this intervention. If successful, we will explore this further in other neoplasms in phase II trials. While TPE does carry risks as an invasive procedure (including hypotension, infection and bleeding) and relies on adequate vascular access, it is not restricted to any particular tumour type and is widely available.

This trial has been approved by the Mayo Clinic IRB. Data will be maintained on a secure database with deidentified patient information. Data will be shared on publication in a peer-reviewed journal without the aid of professional writers. If successful, this trial will lay the ground for phase II studies that will include cancer treated with PD-(L)1 inhibitors which may benefit from TPE such as renal, bladder and lung cancers.

Any modifications to the protocol which may impact on the conduct of the study and potential benefit of the patient or may affect patient safety, including changes of study objectives, study design, patient population, sample sizes, study procedures or significant administrative aspects will require a formal amendment to the protocol. Such amendment will be approved by the Mayo Clinic IRB prior to implementation and health authorities will be notified in accordance with local regulations.

### Biological specimens

Laboratory specimens (blood draws) will be processed for plasma and peripheral blood mononuclear cells (PBMCs). They will be stored in study identity-labelled tubes at −80C until analysis by flow cytometry, ELISA, or other means for future studies.

## Supplementary Material

Reviewer comments

Author's manuscript
