# Supplementary material for: Rescuing Cancer Immunity by Plasma Exchange in Metastatic Melanoma (ReCIPE-M1): protocol for a single-institution, open-label safety trial of plasma exchange to clear sPD-L1 for immunotherapy
Source: BMJ Open. 2022 May 11;12(5):e050112. doi: 10.1136/bmjopen-2021-050112 (PMC9109028; doi:10.1136/bmjopen-2021-050112)
Supplement: Author's manuscript [file bmjopen-2021-050112.draft_revisions.pdf]

BMJ Open is committed to open peer review. As part of this commitment we make the peer review history of every article we publish publicly available.

When an article is published we post the peer reviewers' comments and the authors' responses online. We also post the versions of the paper that were used during peer review. These are the versions that the peer review comments apply to.

The versions of the paper that follow are the versions that were submitted during the peer review process. They are not the versions of record or the final published versions. They should not be cited or distributed as the published version of this manuscript.

BMJ Open is an open access journal and the full, final, typeset and author-corrected version of record of the manuscript is available on our site with no access controls, subscription charges or pay-per-view fees (http://bmjopen.bmj.com).

If you have any questions on BMJ Open's open peer review process please email info.bmjopen@bmj.com

Rescuing Cancer Immunity by Plasma Exchange in Metastatic Melanoma (ReCIPE-M1): An open-label safety trial

Journal:	*BMJ Open*
Manuscript ID	bmjopen-2021-050112
Article Type:	Protocol
Date Submitted by the Author:	11-Feb-2021
Complete List of Authors:	Davidson, Tara M.; Mayo Clinic, Department of Internal Medicine<br>Lucien, Fabrice; Mayo Clinic, Department of Urology<br>Dong, Haidong; Mayo Clinic, Department of Urology<br>Winters, Jeffrey L.; Mayo Clinic, Department of Pathology and Laboratory Medicine<br>Park, Sean S.; Mayo Clinic, Radiation Oncology<br>Orme, Jacob; Mayo Clinic, Division of Medical Oncology<br>Svetomir, Markovic; Mayo Clinic, Division of Medical Oncology
Keywords:	ONCOLOGY, IMMUNOLOGY, Radiobiology < RADIOLOGY & IMAGING

SCHOLARONE™
Manuscripts

Clinical trial design: BMJ Open

Title: Rescuing Cancer Immunity by Plasma Exchange in Metastatic Melanoma (ReCIPE-M1): An open-label safety trial

Authors: Tara M. Davidson[1]; Fabrice Lucien[2]; Svetomir Markovic[3]; Haidong Dong[2]; Jeffrey L. Winters[4]; Sean S. Park[5,*]; Jacob J. Orme[3,*]

[1]Department of Internal Medicine, Mayo Clinic, Rochester, MN

[2]Department of Urology, Mayo Clinic, Rochester, MN

[3]Division of Medical Oncology, Mayo Clinic, Rochester, MN

[4]Department of Pathology and Laboratory Medicine, Mayo Clinic, Rochester, MN

[5]Department of Radiation Oncology, Mayo Clinic, Rochester, MN

*These authors contributed equally

Running title: TPE for melanoma immunotherapy

Keywords: PD-L1, PD-1, soluble PD-L1, sPD-L1

Financial support: Support: NCI CA259236, Lawrence W. and Marilyn W. Matteson Award

COI: Intellectual property has been filed addressing discoveries disclosed in this manuscript. The authors report no other relevant conflicts of interest.

For peer review only - http://bmjopen.bmj.com/site/about/guidelines.xhtml

1 Corresponding Author:

2 Jacob Orme, M.D. Ph.D.

3 200 1st St NW

4 Rochester, MN 55902

5 Phone: 507-284-2511

6 Email: orme.jacob@mayo.edu

7 **Word count: 1,972**

8 **Total figures and tables: 2 figures, 1 table**

For peer review only

For peer review only - http://bmjopen.bmj.com/site/about/guidelines.xhtml

ABSTRACT

Background: Patients with metastatic melanoma rely on PD-(L)1 immunotherapy, but only one third of patients experience treatment response and all initial responders eventually develop resistance. Tumor-derived extracellular vesicles expressing PD-L1 (evPD-L1) and soluble PD-L1 (sPD-L1) in peripheral blood of patients with melanoma limit PD-(L)1 immunotherapy and correlate with poor survival. Therapeutic plasma exchange (TPE) removes immunosuppressive evPD-L1 and sPD-L1. We hypothesize that TPE may rescue and restore anti-melanoma immunity.

Methods: In this two-arm study (NCT04581382), 60 patients with metastatic melanoma progressing on checkpoint inhibition will be accrued. All patients will receive standard of care radiotherapy on days 1-5 and ongoing checkpoint inhibition on day 8 and every 2-3 weeks per standard of care. Patients with baseline sPD-L1 level ≥1.7 ng/ml and adequate clinical capacity will be enrolled in the TPE Intervention Arm and receive TPE on days 5-7 in addition to standard of care radiotherapy and immunotherapy. Other patients will remain in the Standard of Care Arm.

The primary endpoint of the study is to evaluate safety. Secondary endpoints include kinetics of sPD-L1 and evPD-L1 and clinical response by RECIST criteria.

Discussion: This trial will assess the safety and feasibility of TPE in improving outcomes for PD-(L)1 inhibitor immunotherapy in melanoma. If successful, it will lay the ground for Phase II studies that will include cancer treated with PD-(L)1 inhibitors which may benefit from TPE such as renal, bladder, and lung cancers.

For peer review only

Ethics and Dissemination: This trial has been approved by the Mayo Clinic IRB. Data will be maintained on a secure database with de-identified patient information. Data will be shared on publication in a peer-reviewed journal without the aid of professional writers.

ARTICLE SUMMARY

Strengths and limitations of this study:

- First clinical trial to evaluate the safety of TPE and kinetics of immunomodulatory molecules removed by TPE in vivo in melanoma
- Highly validated biomarker studies (sPD-L1 and evPD-L1) known to correlate to outcomes in melanoma
- No randomization to intervention versus control arm in this early Phase I study

For peer review only

For peer review only - http://bmjopen.bmj.com/site/about/guidelines.xhtml

1. INTRODUCTION

The first immunotherapy with interferon was approved by the Food and Drug Administration (FDA) in 1986.[1] Since then, leveraging the body's own immune system to target and kill malignant cells has become the fastest-growing therapeutic approach to cancer worldwide.[2] While early immunotherapies focused on providing immunostimulatory cytokines (*e.g.,* pegilodecakin), more recent approaches have leveraged vaccination (*e.g.,* MAGE-A3), transplant of primed anti-tumor CD8+ T cells (*i.e.,* CAR-T therapy), and inhibiting immune checkpoint signals (*e.g.,* Programmed Death 1 [PD-1] and Programmed Death Ligand 1 [PD-L1] inhibitors like pembrolizumab, nivolumab, and atezolizumab).[3]

The latter approach with PD-(L)1 inhibitors has shown spectacular results in many indications, leading to first-line approvals of checkpoint inhibitor immunotherapy in renal cell carcinoma, non-small cell lung cancer, melanoma, and others.[4-6] Unfortunately, even in these highly promising settings less than half of patients have objective response to PD-(L)1 inhibitor therapy alone.[7] For instance, only about one in three melanomas respond to PD-(L)1 inhibitor pembrolizumab.[6]

Intensive research is focused on identifying causes for intrinsic resistance to PD-(L)1 inhibitors. Some proposed primary resistance mechanisms include insufficient tumor immunogenicity, irreversible T cell exhaustion, MHC dysfunction, resistance to IFN-γ signaling, and an immunosuppressive tumor microenvironment (TME).[8] One known component of the TME is soluble factors, which predict the ability of the immune system to combat cancers. High serum soluble PD-L1 (sPD-L1), for instance, predicts poor treatment response and outcomes in melanoma.[9,10] This also applies to melanomas

For peer review only - http://bmjopen.bmj.com/site/about/guidelines.xhtml

treated with PD-(L)1 immunotherapy.[11] Serum sPD-L1 is produced by ADAM10/ADAM17-dependent and independent mechanisms and has been shown to outcompete PD-(L)1 inhibitors, cause CD8+ T cells apoptosis, and dampen anti-tumor immunity (Figure 1).[12,13] Through a similar mechanism, extracellular vesicle (EV)-associated PD-L1 (evPD-L1) downregulates anti-tumor immunity.[14]

Clearly, novel therapeutic approaches are needed to overcome the immunosuppressive systemic and TME. We recently explored the potential of therapeutic plasma exchange (TPE) in removing sPD-L1 and evPD-L1.[15] In brief, TPE is a procedure for passing blood through an apheresis machine to separate plasma from cellular components and is commonly used clinically for removal of deleterious antibodies.[16] Plasma containing the offending substance is replaced in this procedure with up to 30% crystalloid in the form of normal saline and either donor plasma or 5% human albumin derived from plasma donation. In our previous report, each session of TPE reduced sPD-L1 and evPD-L1 by 70.8% and 73.1%, respectively.[15] This represents the first clinical intervention to remove either of these substances from circulation in vivo.

Despite these important discoveries, several crucial questions remain unanswered. First, while TPE is commonly prescribed in the outpatient setting, it has not been studied specifically in cancer treated with immunotherapy. Second, the kinetics of sPD-L1 and evPD-L1 in patients with melanoma have not previously been determined. Pilot studies to establish the safety profile and durability of TPE-mediated changes in melanoma are warranted.

For peer review only

For peer review only - http://bmjopen.bmj.com/site/about/guidelines.xhtml

2. METHODS

2.1. Overview of study design

This feasibility trial (NCT04581382) will assess (1) the safety of TPE through adverse events monitoring at 1 and 6 months post-therapy (primary outcome), (2) the kinetics of evPD-L1, sPD-L1, and related immunosuppressive metabolites immediately and 2-3 weeks after TPE, and (3) the relative response of patients to combined therapy through response rate, overall survival, and progression-free survival.

2.2. Recruitment, screening procedures and treatment arms

Patients with metastatic melanoma progressing on standard of care on any PD-(L)1 inhibitor are eligible for this study (Table 1, Figure 2). Potentially eligible patients will be approached by the clinical trial coordinator and provide informed consent. 60 patients will be enrolled with a goal of accrued 20 patients to the TPE Intervention Arm. Inclusion criteria include age 18 or older, histologically confirmed melanoma, measurable disease per RECIST criteria, an ECOG status of three or better. Exclusion criteria include pregnancy or nursing. Also excluded are those who consume biotin supplementation as it interferes with accurate measurement of sPD-L1.

Enrolled patients with sPD-L1 level at or above 1.7ng/ml will be enrolled in the Intervention Arm where clinical availability of the procedure allows. Other patients will be enrolled in the Standard of Care Arm. All data will be collected in a deidentified fashion in a password-protected database.

For peer review only - http://bmjopen.bmj.com/site/about/guidelines.xhtml

2.3. Study interventions

All patients will receive standard of care treatment with any PD-(L)1 inhibitor. This standard treatment comprises radiotherapy on days 1-5 and PD-(L)1 inhibitor treatment, on day 8 and every 2-3 weeks thereafter as clinically indicated. Patients in the Intervention Arm, in addition to standard of care, will undergo serial blood draws on day 5 prior to first TPE, day 7 following last TPE and in 2-3 weeks on the day of subsequent dose of immunotherapy. We will assess the kinetics of sPD-L1 and evPD-L1 at these timepoints.

TPE will be performed using centrifugation-based cell separators, either the Fenwal Amicus (Fresenius KABI USA LLC, Lake Zurich IL, USA) or the Spectra Optia (Terumo BCT Inc, Lakewood CO, USA). For each patient, a single plasma volume was exchanged using either peripheral IVs (preferred) or central lines for vascular access if a central line is present. Due to the possibility of sPD-L1 or PD-L1-positive EVs present in donor plasma, donor plasma (*i.e.,* fresh frozen plasma, FFP) will not be used as a replacement fluid. Rather, 5% normal serum human albumin with up to 500 ml of normal saline will be used as the replacement. The volume used is determined by the device based upon patient gender, height, weight, and hematocrit. Anticoagulation will consist of either 500 ml of acid citrate dextrose solution A (ACD-A) or 500 ml of ACD-A with 5000 units of unfractionated heparin. Anticoagulant to blood ratios will be 1:13 when ACD-A is used and 1:26 when ACD-A/Heparin is used. Patients will not receive routine electrolyte replacement but 10 ml of 10% calcium gluconate will be administered by

For peer review only - http://bmjopen.bmj.com/site/about/guidelines.xhtml

slow IV push for signs and symptoms of hypocalcemia related to the ACD-A anticoagulant.

Toxicity assessment using CTCAE version 5.0 will be assessed within 7 days prior to registration as baseline in addition to daily during TPE and 2 weeks post first dose of immunotherapy if receiving nivolumab or 3 weeks post first dose of immunotherapy if receiving pembrolizumab as well as 3 months post-TPE. Safety will also be assessed during any clinically indicated follow-up until progressive disease, death or up to 2 years after registration.

Imaging will be obtained as standard of care and is not stipulated by the study. CT, PET, and/or MRI scans can be used as clinically indicated. Imaging to assess tumor response will be based on RECIST/PERCIST criteria (v1.1) until progression, death or up to 2 years after registration. At least one metastatic lesion will not be radiated to assess response per RECIST and/or PERCIST criteria for those in the Intervention Arm. All oligo-progressive lesions will be radiated for patients in the Standard of Care Arm while on standard-of-care immunotherapy. As patients who progress on immunotherapy are enrolled in this trial, partial or complete response of > 20% will be considered as a positive signal to proceed to a phase II trial. Study materials and procedures were approved by the Mayo Clinic IRB.

2.4 Measuring sPD-L1 and evPD-L1 levels

For measurements of sPD-L1, ELISA will be performed as previously published.[17] Both secreted splice variant and shed sPD-L1 are reliably detected by this ELISA. In brief, paired mouse IgG2 monoclonal antibody clones H1A and B11 against extracellular

For peer review only - http://bmjopen.bmj.com/site/about/guidelines.xhtml

human PD-L1 will be utilized in a capture-detection plate assay using a standard biotinylated antibody and streptavidin-HRP detection method. This assay is specific for sPD-L1 and does not exhibit cross reactivity to other B7-H homologues, nor to evPD-L1. Concentrations are determined by optical density measurements along a known standard curve of recombinant human PD-L1. ELISAs are performed by technicians blinded to the identity of the samples.

For measurements of evPD-L1, flow cytometry will be used as previously published.[18] In brief, plasma samples are centrifuged twice at 2000g to deplete platelets. Resultant platelet-free plasma is analyzed using an A60-Micro Plus Nanoscale Flow Cytometer (Apogee FlowSystems) gating for mid-intensity light angle light scatter and markers of interest. Anti-PD-L1 (Genentech: atezolizumab), antibodies are conjugated to fluorophores (Life Technologies: Alexa-647, PE phycoerythrin, and Alexa-488) and titrated prior to use. Nanoscale flow cytometer calibration is performed using a standard reference bead mix as previously published. Flow cytometry is performed by technicians blinded to the identity of the samples.

2.4. Endpoints

The primary endpoint of this trial is to assess the safety of TPE in patients with melanoma receiving PD-(L)1 immunotherapy. The secondary endpoints of this trial are (i) the kinetics of sPD-L1 and evPD-L1 removal in patients with melanoma receiving PD-(L)1 immunotherapy, (ii) overall response rate as a proportion of patients with a tumor response of PR or CR at least 4 weeks apart, and (iii) progression-free and overall survival.

For peer review only

2.5. Data collection schedule

The principal investigator and study statistician will review the study at least monthly to identify accrual, adverse events, and any endpoint problems that might be developing. The trial is monitored continually by the study team who are notified of every grade 4 or 5 event in real time.

2.6. Patient and Public Involvement

No patient involved.

3. DISCUSSION

Programmed death-ligand 1 (PD-L1, B7-H1, CD274) is primarily expressed by dendritic cells (DCs), monocytes, and other cells in response to inflammatory cytokines.[3,19-22] PD-L1 signals through receptor programmed death 1 (PD-1, CD279) on the surface of activated CD8+ T cells to negatively regulate immunity. [3,23,24] Many tumors express PD-(L)1 resulting in lymphocyte exhaustion and immune invasion. Since the first clinical trial for Nivolumab in 2006, the field has focused on more consistently preventing PD-1/PD-L1 signaling in cancer.

PD-L1 is classically thought to be a membrane-bound ligand. Recently, however, three additional functional forms of *extracellular* PD-L1 have been identified. Soluble PD-L1 can be produced by transcribed PD-L1 splice variants, of which four variants have been identified in melanoma.[11,25] Metalloproteases ADAM10 and ADAM17 can also cleave the PD-L1 ectodomain from tumor cells and extracellular vesicles (EVs) to form sPD-L1.[13] Lastly, tumor cells release extracellular vesicles bearing intact PD-L1 (evPD-L1).[14]

For peer review only

The role of sPD-1 and evPD-L1 are not yet fully understood. However, sPD-L1 levels are significantly higher in patients with metastatic melanoma at baseline compared to healthy volunteers and elevated levels predict inferior outcomes.[11,13,26] This implies that the same inflammatory/immune response that is known to play a role in PD-L1 expression also increases the rate of extracellular PD-L1 production. In one study, PD-L1 cell surface expression paralleled sPD-L1 secretion in response to cytokines in melanoma cells.[11] Some cancer cells secrete a majority of their PD-L1 on exosomes, even with only minimal amounts of cellular PD-L1 found.[14,25] The leading hypothesis is that the tumor microenvironment, including non-neoplastic cells, may also be contributing sPD-L1 and evPD-L1 to the tumor microenvironment.

While the particular roles of these extracellular PD-L1 forms continue to be elucidated, the results of sPD-L1 and evPD-L1 signaling are clear from both in vitro and in vivo studies: sPD-L1 and evPD-L1 outcompete PD-(L)1 inhibitors, initiate apoptosis in CD8+ T cells, and cause systemic immunosuppression by limiting the ability of healthy peripheral blood mononuclear cells to kill tumor cells. [13,25,27] A majority of studies in multiple malignancies find a negative relationship between high sPD-L1 and outcomes.[9,10,17,28-36] In melanoma, Zhou et al showed that high pretreatment levels of sPD-L1 were associated with increased likelihood of progressive disease in patients with melanoma treated by CTLA-4 or PD-1 blockade.[11] For these reasons, patients with metastatic melanoma form our initial study population.

Radiation increases the production of extracellular PD-L1 through inflammatory cytokines and other mechanisms.[37,38] We hypothesize that TPE will reliably and safely

For peer review only - http://bmjopen.bmj.com/site/about/guidelines.xhtml

reduce the levels of sPD-L1 and evPD-L1 in our study patients. We hypothesize that this will alleviate the immunosuppressive microenvironment caused by extracellular PD-L1 as well as minimize any competition for PD-(L)1 inhibitors prior to these study patients receiving their dose of PD-(L)1 inhibitor therapy. We hope to see improved response to these agents through this intervention. If successful, we will explore this further in other neoplasms in Phase II trials. While TPE does carry risks as an invasive procedure and relies on adequate vascular access, it is not restricted to any particular tumor type and is widely available.

REFERENCES

1. Golomb HM, Jacobs A, Fefer A, et al. Alpha-2 interferon therapy of hairy-cell leukemia: a multicenter study of 64 patients. *J Clin Oncol.* 1986;4(6):900-905.
2. Dighe S. Immune Checkpoint Inhibitors (ICI) Market by Type (CTLA-4 Inhibitor, PD-1 Inhibitor, and PD-L1 Inhibitor) and Application (Lung Cancer, Bladder Cancer, Melanoma, Hodgkin Lymphoma, and Others). *https://wwwalliedmarketresearchcom/immune-check-point-inhibitors-market (2018).*
3. Dong H, Strome SE, Salomao DR, et al. Tumor-associated B7-H1 promotes T-cell apoptosis: a potential mechanism of immune evasion. *Nat Med.* 2002;8(8):793-800.
4. Hellmann MD, Paz-Ares L, Bernabe Caro R, et al. Nivolumab plus Ipilimumab in Advanced Non-Small-Cell Lung Cancer. *N Engl J Med.* 2019;381(21):2020-2031.
5. Rini BI, Plimack ER, Stus V, et al. Pembrolizumab plus Axitinib versus Sunitinib for Advanced Renal-Cell Carcinoma. *N Engl J Med.* 2019;380(12):1116-1127.
6. Robert C, Schachter J, Long GV, et al. Pembrolizumab versus Ipilimumab in Advanced Melanoma. *N Engl J Med.* 2015;372(26):2521-2532.
7. Haslam A, Prasad V. Estimation of the Percentage of US Patients With Cancer Who Are Eligible for and Respond to Checkpoint Inhibitor Immunotherapy Drugs. *JAMA Netw Open.* 2019;2(5):e192535.
8. Lei Q, Wang D, Sun K, Wang L, Zhang Y. Resistance Mechanisms of Anti-PD1/PDL1 Therapy in Solid Tumors. *Front Cell Dev Biol.* 2020;8:672.
9. Dronca RS, Harrington S, Jegapragasan M, et al. Association of soluble PD-L1 (sPD-L1) with decreased survival in metastatic melanoma. *Journal of Clinical Oncology.* 2017;35(7_suppl):4-4.
10. Dronca RS, Mansfield AS, Liu X, et al. Bim and soluble PD-L1 (sPD-L1) as predictive biomarkers of response to anti-PD-1 therapy in patients with melanoma and lung carcinoma. *Journal of Clinical Oncology.* 2017;35(15_suppl):11534-11534.

For peer review only - http://bmjopen.bmj.com/site/about/guidelines.xhtml

11. Zhou J, Mahoney KM, Giobbie-Hurder A, et al. Soluble PD-L1 as a Biomarker in Malignant Melanoma Treated with Checkpoint Blockade. *Cancer Immunol Res.* 2017;5(6):480-492.
12. Mahoney KM, Shukla SA, Patsoukis N, et al. A secreted PD-L1 splice variant that covalently dimerizes and mediates immunosuppression. *Cancer Immunol Immunother.* 2019;68(3):421-432.
13. Orme JJ, Jazieh KA, Xie T, et al. ADAM10 and ADAM17 cleave PD-L1 to mediate PD-(L)1 inhibitor resistance. *Oncoimmunology.* 2020;9(1):1744980.
14. Chen G, Huang AC, Zhang W, et al. Exosomal PD-L1 contributes to immunosuppression and is associated with anti-PD-1 response. *Nature.* 2018;560(7718):382-386.
15. Orme JJ, Enninga EAL, Lucien-Matteoni F, et al. Therapeutic plasma exchange clears circulating soluble PD-L1 and PD-L1-positive extracellular vesicles. *J Immunother Cancer.* 2020;8(2).
16. Padmanabhan A, Connelly-Smith L, Aqui N, et al. Guidelines on the Use of Therapeutic Apheresis in Clinical Practice - Evidence-Based Approach from the Writing Committee of the American Society for Apheresis: The Eighth Special Issue. *J Clin Apher.* 2019;34(3):171-354.
17. Frigola X, Inman BA, Lohse CM, et al. Identification of a Soluble Form of B7-H1 That Retains Immunosuppressive Activity and Is Associated with Aggressive Renal Cell Carcinoma. *Clinical Cancer Research.* 2011;17(7):1915-1923.
18. Gomes J, Lucien F, Cooper TT, et al. Analytical Considerations in Nanoscale Flow Cytometry of Extracellular Vesicles to Achieve Data Linearity. *Thromb Haemost.* 2018;118(9):1612-1624.
19. Dong H, Zhu G, Tamada K, Chen L. B7-H1, a third member of the B7 family, co-stimulates T-cell proliferation and interleukin-10 secretion. *Nat Med.* 1999;5(12):1365-1369.
20. Gibbons Johnson RM, Dong H. Functional Expression of Programmed Death-Ligand 1 (B7-H1) by Immune Cells and Tumor Cells. *Front Immunol.* 2017;8:961.
21. Keir ME, Butte MJ, Freeman GJ, Sharpe AH. PD-1 and its ligands in tolerance and immunity. *Annu Rev Immunol.* 2008;26:677-704.
22. Yamazaki T, Akiba H, Iwai H, et al. Expression of Programmed Death 1 Ligands by Murine T Cells and APC. *The Journal of Immunology.* 2002;169(10):5538-5545.
23. Chemnitz JM, Parry RV, Nichols KE, June CH, Riley JL. SHP-1 and SHP-2 Associate with Immunoreceptor Tyrosine-Based Switch Motif of Programmed Death 1 upon Primary Human T Cell Stimulation, but Only Receptor Ligation Prevents T Cell Activation. *The Journal of Immunology.* 2004;173(2):945-954.
24. Riley JL. PD-1 signaling in primary T cells. *Immunol Rev.* 2009;229(1):114-125.
25. Poggio M, Hu T, Pai CC, et al. Suppression of Exosomal PD-L1 Induces Systemic Anti-tumor Immunity and Memory. *Cell.* 2019;177(2):414-427 e413.
26. Chen Y, Wang Q, Shi B, et al. Development of a sandwich ELISA for evaluating soluble PD-L1 (CD274) in human sera of different ages as well as supernatants of PD-L1+ cell lines. *Cytokine.* 2011;56(2):231-238.
27. Parry RV, Chemnitz JM, Frauwirth KA, et al. CTLA-4 and PD-1 receptors inhibit T-cell activation by distinct mechanisms. *Mol Cell Biol.* 2005;25(21):9543-9553.

For peer review only - http://bmjopen.bmj.com/site/about/guidelines.xhtml

28. Costantini A, Julie C, Dumenil C, et al. Predictive role of plasmatic biomarkers in advanced non-small cell lung cancer treated by nivolumab. *Oncoimmunology.* 2018;7(8):e1452581.
29. Finkelmeier F, Canli Ö, Tal A, et al. High levels of the soluble programmed death-ligand (sPD-L1) identify hepatocellular carcinoma patients with a poor prognosis. *Eur J Cancer.* 2016;59:152-159.
30. Ha H, Nam AR, Bang JH, et al. Soluble programmed death-ligand 1 (sPDL1) and neutrophil-to-lymphocyte ratio (NLR) predicts survival in advanced biliary tract cancer patients treated with palliative chemotherapy. *Oncotarget.* 2016;7(47):76604-76612.
31. Okuma Y, Hosomi Y, Nakahara Y, Watanabe K, Sagawa Y, Homma S. High plasma levels of soluble programmed cell death ligand 1 are prognostic for reduced survival in advanced lung cancer. *Lung Cancer.* 2017;104:1-6.
32. Sideras K, de Man RA, Harrington SM, et al. Circulating levels of PD-L1 and Galectin-9 are associated with patient survival in surgically treated Hepatocellular Carcinoma independent of their intra-tumoral expression levels. *Scientific Reports.* 2019;9(1):10677.
33. Wang H, Wang L, Liu WJ, et al. High post-treatment serum levels of soluble programmed cell death ligand 1 predict early relapse and poor prognosis in extranodal NK/T cell lymphoma patients. *Oncotarget.* 2016;7(22):33035-33045.
34. Wang L, Wang H, Chen H, et al. Serum levels of soluble programmed death ligand 1 predict treatment response and progression free survival in multiple myeloma. *Oncotarget.* 2015;6(38):41228-41236.
35. Zhang J, Gao J, Li Y, et al. Circulating PD-L1 in NSCLC patients and the correlation between the level of PD-L1 expression and the clinical characteristics. *Thorac Cancer.* 2015;6(4):534-538.
36. Zheng Z, Bu Z, Liu X, et al. Level of circulating PD-L1 expression in patients with advanced gastric cancer and its clinical implications. *Chin J Cancer Res.* 2014;26(1):104-111.
37. Ding XC, Wang LL, Zhu YF, et al. The Change of Soluble Programmed Cell Death-Ligand 1 in Glioma Patients Receiving Radiotherapy and Its Impact on Clinical Outcomes. *Front Immunol.* 2020;11:580335.
38. Kim HJ, Park S, Kim KJ, Seong J. Clinical significance of soluble programmed cell death ligand-1 (sPD-L1) in hepatocellular carcinoma patients treated with radiotherapy. *Radiother Oncol.* 2018;129(1):130-135.

For peer review only

ACKNOWLEDGMENTS

ReCIPE-M1 is supported by grants from the Lawrence W. and Marilyn W. Matteson Fund at Mayo Clinic and R21 CA259236-01 from the National Cancer Institute. Some images were created with BioRender.

For peer review only - http://bmjopen.bmj.com/site/about/guidelines.xhtml

DECLARATION OF INTEREST STATEMENT

Intellectual property has been filed addressing discoveries disclosed in this manuscript.

The authors report no other relevant conflicts of interest.

For peer review only

For peer review only - http://bmjopen.bmj.com/site/about/guidelines.xhtml

TABLES

Table 1.

INCLUSION CRITERIA		ARM ASSIGNMENTS	
**Inclusion Criteria**	**Exclusion Criteria**		
≥18 years<br>Histologic melanoma<br>Measurable disease<br>ECOG ≤ 3<br>Ability to consent	Biotin supplement use<br>Pregnant or nursing	**Intervention Arm**	sPD-L1 ≥1.7 ng/ml *AND* available clinical capacity
		**Standard of Care Arm**	sPD-L1 <1.7 ng/ml *OR* unavailable clinical capacity

Table 1. Study inclusion and exclusion criteria. Eligible patients for the study will be consenting adults with histologically confirmed melanoma receiving PD-(L)1 immunotherapy who are referred for stereotactic body radiation therapy (SBRT). Patients who are receiving biotin supplements, are pregnant, or are nursing are excluded. Patients with elevated sPD-L1 on registration will enter the Intervention Arm where clinical capacity is sufficient. Patients with low sPD-L1 or who enter the trial when clinical capacity is not available will enter the Standard of Care Arm.

For peer review only - http://bmjopen.bmj.com/site/about/guidelines.xhtml

FIGURE LEGENDS

Figure 1. Extracellular PD-L1 Outcompetes Immune Checkpoint Inhibitors to Block Anti-tumor Immunity. While PD-(L)1 inhibitors prevent tumor cell downregulation of T cells, tumor cells secrete evPD-L1 and sPD-L1 (either cleaved by ADAM10/ADAM17 or as splice variants) that outcompete PD-(L)1 inhibitors and downregulate systemic anti-cancer immunity.

Figure 2. Study design in two arms. In brief, eligible patients undergo a registration blood draw and vascular access evaluation to determine appropriate study arm. Patients with elevated baseline sPD-L1 (≥1.7 ng/ml) will enter the Intervention Arm if there is adequate clinical capacity. Other patients will enter the Standard of Care Arm.

For peer review only

For peer review only - http://bmjopen.bmj.com/site/about/guidelines.xhtml

AUTHOR STATEMENT

TD wrote the manuscript. JJO developed hypotheses and concepts, performed initial studies, generated figures and helped write the manuscript. HD, SSP and JLW provided mentorship, equipment, facilities, edits and funding. FL and SM provided additional logistical input, planning and edits. Sponsors and funders have had and will have NO role in study design; collection, management, analysis, or interpretation of data; writing of any report; nor the decision to submit any report for publication. Data will be shared on publication in a peer-reviewed journal without the aid of professional writers.

BIOLOGICAL SPECIMENS

Laboratory specimens (blood draws) will be processed for plasma and peripheral blood mononuclear cells (PBMCs). They will be stored in study identity-labeled tubes at -80C until analysis by flow cytometry, ELISA, or other means for future studies.

DATA STATEMENT

Deidentified patient information will be made available in a public data repository on publication.

For peer review only - http://bmjopen.bmj.com/site/about/guidelines.xhtml

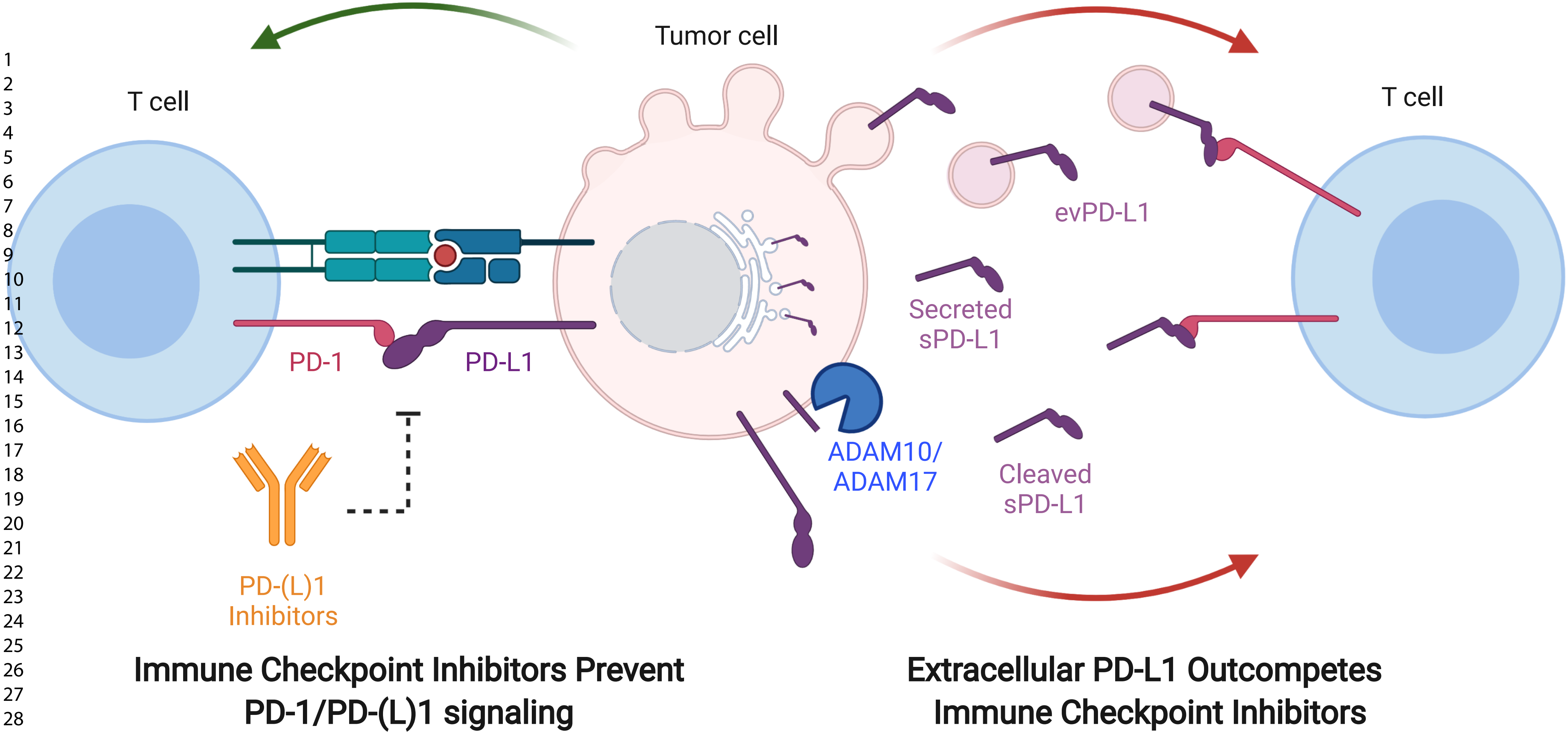
Tumor cell
T cell
T cell
PD-1
PD-L1
PD-(L)1
Inhibitors
evPD-L1
Secreted
sPD-L1
ADAM10/
ADAM17
Cleaved
sPD-L1
Immune Checkpoint Inhibitors Prevent
PD-1/PD-(L)1 signaling
Extracellular PD-L1 Outcompetes
Immune Checkpoint Inhibitors

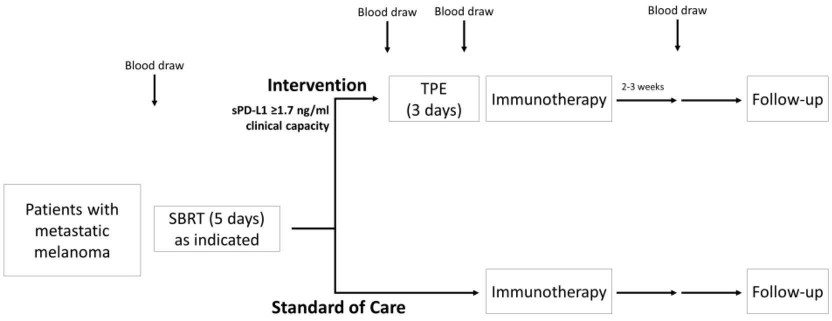

Figure 2. Study design in two arms. In brief, eligible patients undergo a registration blood draw and vascular access evaluation to determine appropriate study arm. Patients with elevated baseline sPD-L1 (≥1.7 ng/ml) will enter the Intervention Arm if there is adequate clinical capacity. Other patients will enter the Standard of Care Arm.

1092x1016mm (96 x 96 DPI)

SPIRIT 2013 REGISTRATION DATA SET

Data category	Information
Primary registry and trial identifying number	ClinicalTrials.gov<br>NCT04581382
Date of registration in primary registry	October 9, 2020
Secondary identifying numbers	MC200703 v2
Source(s) of monetary or material support	NCI CA259236 (R21)<br>Lawrence W. and Marilyn W. Matteson Award
Primary sponsor	Mayo Clinic
Secondary sponsor(s)	
Contact for public queries	JJO, orme.jacob@mayo.edu
Contact for scientific queries	JJO, orme.jacob@mayo.edu
Public title	Rescuing Cancer Immunity by Plasma Exchange in Metastatic Melanoma (ReCIPE-M1): An open-label safety trial
Scientific title	*Rescuing Cancer Immunity by Plasma Exchange in Metastatic Melanoma (ReCIPE-M1): An open-label safety trial*
Countries of recruitment	USA
Health condition(s) or problem(s) studied	Metastatic melanoma
Intervention(s)	Intervention Arm: SBRT (stereotactic body radiation therapy), TPE (therapeutic plasma exchange), ICI (immunotherapy)

Data category	Information
	Standard of Care Arm: SBRT (stereotactic body radiation therapy), ICI (immunotherapy)
Key inclusion and exclusion criteria	Ages eligible for study: ≥18 years<br>Sexes eligible for study: both<br>Accepts healthy volunteers: no
	Inclusion criteria: adult patient (≥ 18 years), metastatic melanoma, measurable disease per RECIST criteria, ECOG 3 or better
	Exclusion criteria: pregnancy or nursing, consumption of biotin supplements
Study type	Interventional
	Allocation: selection by presentation criteria
	Primary purpose: treatment
	Phase I
Date of first enrolment	December 2020
Target sample size	60
Recruitment status	Recruiting
Primary outcome(s)	Safety (adverse events at 1 and 6 months)
Key secondary outcomes	kinetics of evPD-L1, sPD-L1, and related immunosuppressive metabolites immediately and 2-3 weeks after TPE<br><br>relative response of patients to combined therapy through response rate<br><br>overall survival<br><br>progression-free survival.

1

2

2

Mayo Clinic: Department of Radiation Oncology

MC200703: Radiation Therapy, Plasma Exchange, and Immunotherapy in Melanoma

Study Chairs: Jacob Orme, M.D., Ph.D.
Mayo Clinic
Department of Radiation Oncology
200 First Street SW
Rochester MN 55905

Study Co-chairs: Sean Park, M.D., Ph.D., MS
Jeffrey Winters, M.D.
Haidong Dong, M.D., Ph.D.
Fabrice Lucien, Ph.D.
Svetomir Markovic, M.D., Ph.D.
Matthew Block, M.D., Ph.D.
Robert McWilliams, M.D.
Lisa Kottschade, APRN, C.N.P.

Statistician: Nathan Foster, MS √

Study Contributor(s): Dawn M. Deno, BS, CCRP √

√ Study contributor(s) not responsible for patient care

Document History

Version 1	4/2/2020
Version 2	10/14/2020
Version 3	2/8/2021

For peer review only

Protocol Resources

Questions:	Contact Name:
Patient eligibility*, test schedule, treatment delays/interruptions/adjustments, dose modifications, adverse events, forms completion and submission, adverse events, forms completion and submission	Study Coordinator – Sarah Aug<br>Phone: 507-266-2348<br>E-mail: aug.sarah@mayo.edu
Protocol document, consent form	Dawn M. Deno, BS, CCRP<br>Phone: 507-293-7964<br>E-mail: deno.dawn@mayo.edu
Regulatory issues	Justine Buroker, MBA, CCRP<br>Phone: 507-293-0624<br>E-mail: buroker.justine@mayo.edu
Statistician	Nathan Foster, MS<br>Phone: 507-284-5051<br>E-mail: foster.nathan@mayo.edu

For peer review only

Table of Contents

Protocol Resources 2
Table of Contents 3
Study Schema 4
List of Abbreviations 5
1. Background 6
2. Goals 8
3. Patient Eligibility 9
4. Test Schedule 11
5. Grouping Factors: 13
6. Registration Procedures 14
7. Protocol Treatment 15
8. Dosage Modification Based on Adverse Events 17
9. Ancillary Treatment/Supportive Care 18
10. Adverse Event (AE) Monitoring and Reporting 19
11. Treatment Evaluation/Measurement of Effect 25
12. Descriptive Factors 33
13. Treatment/Follow–up Decision at Evaluation of Patient 34
14. Body Fluid Biospecimens 35
15. Drug Information 37
16. Statistical Considerations and Methodology 39
17. Pathology Considerations/Tissue Biospecimens 43
18. Records and Data Collection Procedures 44
19. Budget 46
20. References 47
Appendix I ECOG Performance Status 49
Appendix II - Immune-Related Adverse Events Questionnaire 50

For peer review only

Study Schema

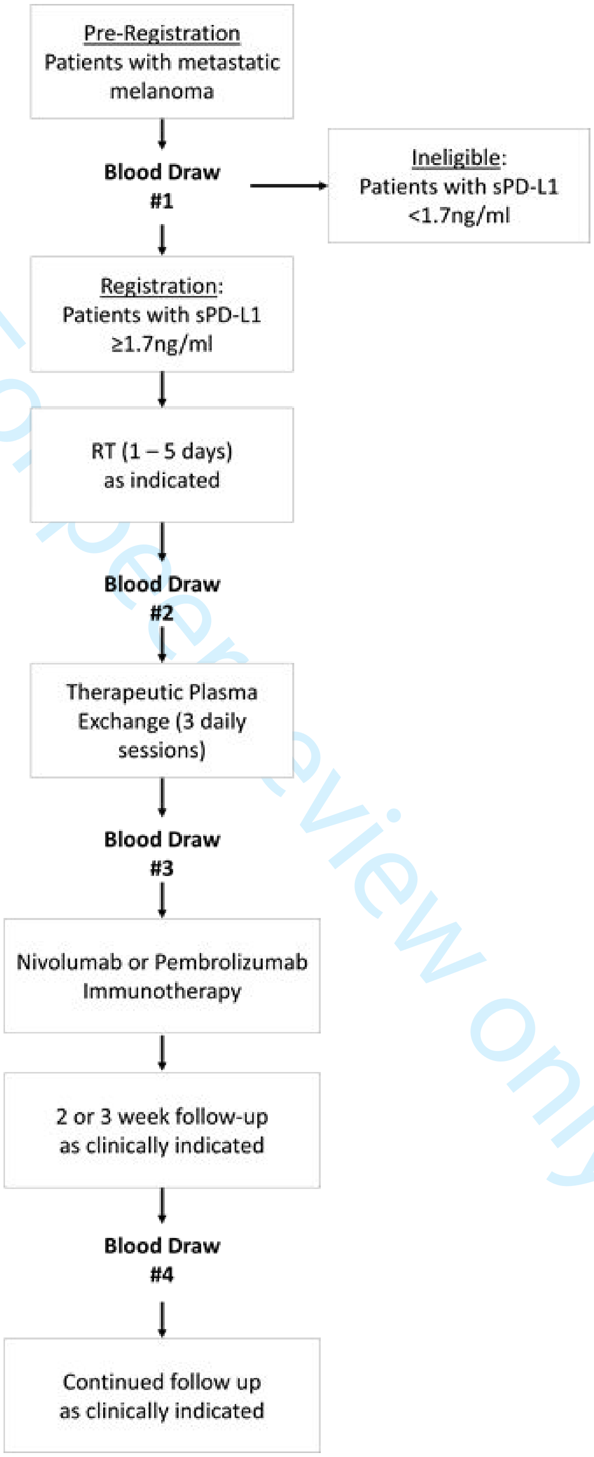

For peer review only

List of Abbreviations

AE	Adverse Event/Adverse Experience
CFR	Code of Federal Regulations
CRF	Case Report Form
DSMB	Data and Safety Monitoring Board
FDA	Food and Drug Administration
GCP	Good Clinical Practice
HIPAA	Health Insurance Portability and Accountability Act
PBT	Proton beam therapy
PI	Principal Investigator
PHI	Protected Health Information
SAE	Serious Adverse Event/Serious Adverse Experience
IRB	Institutional Review Board
ReRT	Re-irradiation
OS	Overall survival
PFS	Progression-free survival
LRC	Locoregional control
RTOG	Radiation Therapy Oncology Group
ECOG	Eastern Cooperative Oncology Group
SFO	Single Field Optimization
MFO	Multi Field Optimization
IFSO	Individual Field Simultaneous Optimization
IMPT	Intensity Modulated Proton Therapy
SPB	Scanning Proton Beam
GTV	Gross Tumor Volume
CTV	Clinical Tumor Volume
OTV	Optimization Target Volume
STV	Scanning Target Volume
PRV	Planning Risk Volume
OAR	Organs at Risk
RU	Range Uncertainty
PET	Positron Emission Tomography
QoL	Quality of Life
EDC	Electronic Data Collection
LET	Linear Energy Transfer
SOC	System Organ Class
RECIST	Response Evaluation Criteria in Solid Tumors
ULN	Upper limit of normal
ORR	Objective Response Rate

For peer review only

1. Background

1.1 Immunotherapy resistance

Almost 3 million patients will receive anti-PD-1 or anti-PD-L1 (henceforth "PD-(L)1 inhibitor") immune checkpoint inhibitors (ICI) to treat their cancer [1], and yet merely 1 in 8 will respond to the treatment [2]. Furthermore, despite initial response to ICI, a majority of the patients succumb to their cancer due to the development of ICI resistance [3]. Recently, we and others have discovered extracellular forms of PD-L1 that mediate PD-(L)1 inhibitor resistance (Fig 1). First, tumors transcribe and secrete soluble PD-L1 (sPD-L1) splice variants [4], [5], and enzymes ADAM10 and ADAM17 also shed sPD-L1 ectodomain directly from the tumor cell surface [6], [7]. Second, tumors generate extracellular vesicles (EVs) bearing surface PD-L1 (evPD-L1) [8], [9]. These extracellular forms of PD-L1 outcompete PD-(L)1 inhibitors, engage and kill CD8+ T cells, and suppress anti-tumor immunity (Fig 1). These forms of PD-L1 at high levels in patient plasma predict poor outcomes in many malignancies [4], [10]–[15].

Thus, *there is a critical need to develop methods to inhibit or remove extracellular PD-L1*. In the absence of such discoveries, most patients who do not respond to PD-(L)1 inhibitors will continue to experience poor survival.

Fig 1 – Extracellular forms of PD-L1 (sPD-L1 and evPD-L1) cause resistance to PD-(L)1 inhibitor therapy in cancer by outcompeting PD-(L)1 inhibitors, killing CD8+ T cells, and limiting anti-tumor immunity.

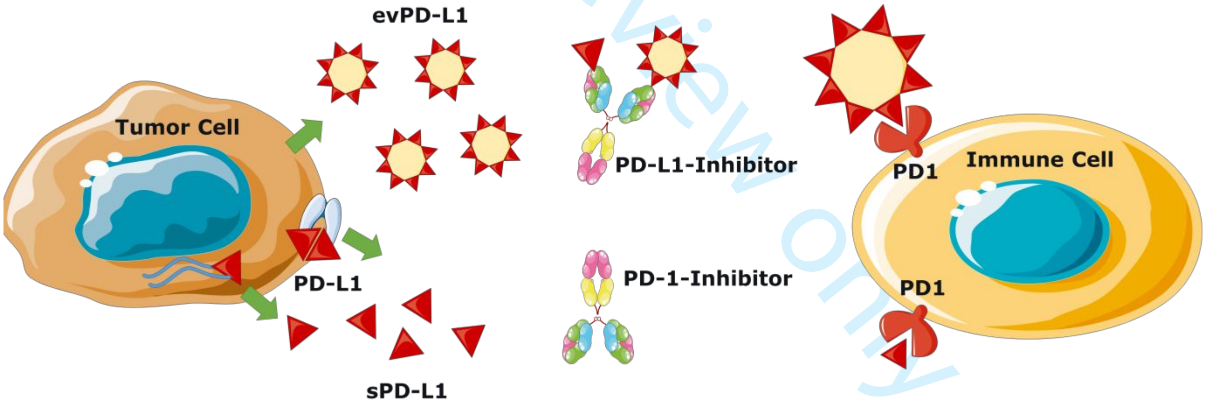

1.2 Therapeutic plasma exchange clears evPD-L1 and sPD-L1

Therapeutic plasma exchange (TPE) is a procedure in which blood is removed from the body and processed to eliminate non-diffusing substances confined to the plasma (Fig 2A). TPE is commonly used clinically to remove pathogenic antibodies in patients with severe autoimmune disease. In our pilot study of 24 patients, TPE significantly depleted sPD-L1 (Fig 2B) and evPD-L1 (Fig 4C) by 70.8% and 73.1%, respectively [16]. This depletion is compounded over three consecutive TPE sessions.

For peer review only

Fig 2 – Therapeutic Plasma Exchange (TPE) removes sPD-L1 and evPD-L1 from circulation. (A) In TPE, blood is removed and centrifuged to remove substances, such as antibodies, from systemic circulation. (B,C) TPE removed an average of 70.8% of sPD-L1 and 73.1% of evPD-L1 from the blood in one session.

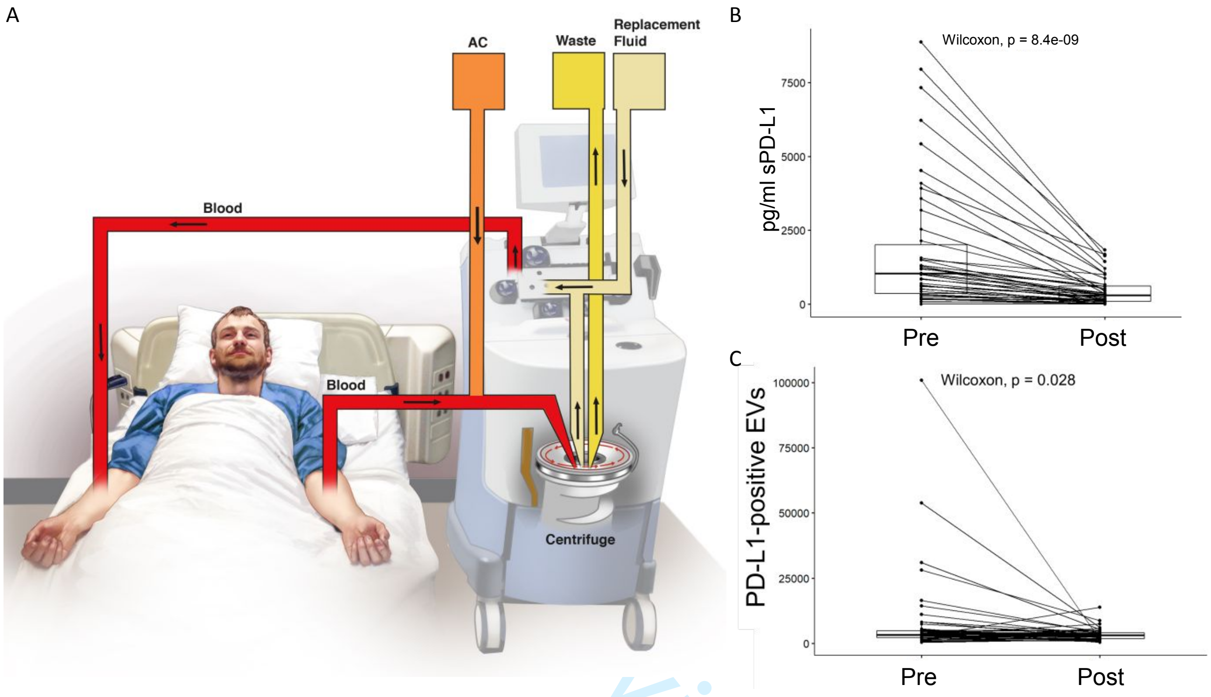

1.3 Defining the kinetics of sPD-L1 and evPD-L1 in melanoma

The *rationale* for this study is that its successful completion will define the kinetic quality of sPD-L1 and evPD-L1 depletion and regeneration in melanoma, without which a full Phase I clinical trial of TPE PD-(L)1 inhibitor rescue cannot be performed. Patients with advanced melanoma who progress on first-line ICI will be enrolled in this study (n=36). Patients with high sPD-L1 levels (> 1.7 ng/ml), ECOG 0-1, life expectancy > 6 months are eligible. After standard-of-care radiation therapy, eligible patients will undergo 3 days of TPE treatment, after which baseline sPD-L1 and evPD-L1 will be measured before standard-of-care immunotherapy is administered. Just prior to the next indicated immunotherapy 2-3 weeks later, sPD-L1 and evPD-L1 will be measured again and compared for regeneration.

2. Goals

2.1. Primary Goal

2.1.1. To determine the kinetics of sPD-L1 removal and regeneration by plasma exchange in patients with melanoma.

2.2. Secondary Goals

2.2.1. To observe response at 3 months after plasma exchange plus immunotherapy.

2.2.2. To observe ongoing response at approximate 3 month intervals after plasma exchange plus immunotherapy.

2.3. Correlative Research

2.3.1. To determine the effects of plasma exchange on immune cell function.

2.3.2. To observe the kinetics of extracellular vesicles (EVs) after plasma exchange in patients with melanoma.

For peer review only

3. Patient Eligibility

3.1. Inclusion Criteria

3.1.1. Age ≥18 years.

3.1.2. Histological confirmation of melanoma. Patients may have completed biopsy outside of Mayo Clinic, but there must be an internal review done to confirm diagnosis prior to confirming eligibility.

3.1.3. Measurable or non-measurable disease as defined in Section 11.0 ~~or~~ .Patient must meet one of these criteria.

3.1.4. ECOG Performance Status (PS) ≤ 3 (Appendix I).

3.1.5. sPD-L1 levels >1.7ng/ml by ELISA [17]

3.1.6. Feasible vascular access as determined by study apheresis technician (either by peripheral intravenous access *or* by placement of a temporary central line)

3.1.7. Negative pregnancy test done ≤7 days prior to radiation therapy, for women of childbearing potential only.

3.1.8. Provide written informed consent.

3.1.9. Willing to return to enrolling institution for follow-up (during the Active Monitoring Phase of the study).

3.1.10. Willing to provide blood samples for correlative research purposes (see Section 6).

3.2. Exclusion Criteria

3.2.1. Any of the following:

3.2.1.1. Persons taking a biotin supplement

3.2.1.2. sPD-L1 level <1.7ng/ml by ELISA [17]

For peer review only

3.2.1.3. Pregnant or nursing women

3.2.1.4. Men or women of childbearing potential who are unwilling to employ adequate contraception

3.2.1.5 ~~Patients that are to receive an invasive procedure(s) within 48 hours prior to receiving TPE; this includes placement of central line.~~

For peer review only

4. Test Schedule

Tests and procedures	Screening	Radiation Therapy	Therapeutic Plasma Exchange[R, 4]		Observation/Follow up until 2 years post registration	
	≤_7_ days prior to registration	*Following Last Fraction of RT*	*Daily during plasma exchange (TPE)*	*Following last plasma exchange (TPE)*	*- Pre-2nd IO[10]*	*At every clinically indicated follow up[2]*
Toxicity Assessments; CTCAE v5.0[S]	X[6]		X[5]	X[5]	X[6]	X[6]
Pregnancy test[1,S]	X					
Vascular Assessment	X[9]					
CBC for Apheresis machine set up[S]	X[8]					
Research blood specimens (see Section 14.0)[R]	X[8]	X[7, 3]		X[7, 3]	X	
Routine Imaging[S]						X
Temporary central line placement (in patients without adequate venous access)[R]	X[11]					
Temporary central line removal (in patients with line)[R]				X		

Radiation Treatment = 1 to 5 fractions, 20 to 60 Gy total, clinician's choice
IO = Immunotherapy, clinician's choice pembrolizumab or nivolumab

1. For women of childbearing potential only. Must be done *≤7 days* prior to beginning Radiation Treatment as a standard of care procedure.

2. Follow up assessments will be completed as per clinician's discretion, which generally takes place every 3-6 months. Local oncologist's standard of care assessments are acceptable.

3. Blood to becollected in Therapeutic Apheresis Treatment Unit – Charlton 8A.

4. The plasma exchange treatments to begin following completion of RT (same day) or the

For peer review only

following day and will be done in 3 consecutive days.

5. Standard of care AE assessments during exchange will be completed daily by Study Apheresis RN, while doing plasma exchange treatment

6. To be assessed by Medical Oncology using irAE toxicity assessment

7. Following the last fraction of RT (Pre-Initial TPE) and Post-Final TPE 8. Must have results available within 2 weeks of starting TPE for machine set up.

9. Vein access for TPE must be determined by study apheresis technician

10. 2 weeks post first IO if receiving **nivolumab**, 3 weeks post first IO if receiving **pembrolizumab**

11. Eligible, enrolled patients determined to require central line placement will undergo Mahurkar line placement by interventional radiology 48-72 hours prior to first TPE procedure. Central line will be removed at apharesis unit at completion of final plasma exchange.

R Research funded (see Section 19.0)

S Standard of care

4.1. Observation/Follow-up

	**Observation/Follow-up[1]**			
	As indicated until PD	At PD	Death	New Primary
Event Monitoring	X	X	X	At each occurrence

1. If a patient is still alive 2 years after registration, no further follow-up is required. Follow up will include: Survival status, progression status, and toxicity assessment.

For peer review only

5. Grouping Factors:

5.1. High versus low baseline evPD-L1 (cutoffs >1 million or 0.05%)

For peer review only

6. Registration Procedures

6.1. Registration

Patient will be registered to the study when they have consented, met eligibility criteria and have been logged into the Research Participant Tracking System (Ptrax).

6.1.1. Verify the following procedures are completed prior to Registration to the study.

6.1.2. IRB approval at the registering institution

6.1.3. Existence of a signed informed consent

6.1.4. Patient eligibility complete

Note: Radiation Therapy on this protocol must be performed at Mayo Clinic Rochester under the supervision of a radiation oncologist.

For peer review only

7. Protocol Treatment

7.1. Radiation Therapy (RT) if indicated

Given per treating radiation oncologist discretion on days D1 through D5. The prescription dose is expected to be up to 60 Gy delivered in up to 5 fractions but not stipulated by the study. Treatment will be delivered daily except on weekends and holidays at the discretion of the treating radiation oncologist.

7.2. Line placement if indicated

In patients without adequate peripheral iv access as determined by apheresis unit nursing staff, a temporary Mahurkar central line will be placed by interventional radiology to enable therapeutic plasma exchange. This will be performed on D2 or D3, at least 48 hours prior to first therapeutic plasma exchange session. Line will be removed by trained apheresis unit staff after final therapeutic plasma exchange session.

~~7.2.~~7.3. Therapeutic plasma exchange (TPE)

Three sessions performed on consecutive days, D5 through D7 or D4 through D6 before immunotherapy on D7. This procedure is *not* performed prior to subsequent immunotherapy in future cycles.

~~7.3.~~7.4. Concurrent immunotherapy (IO)

According to clinician preference, either pembrolizumab or nivolumab will be given on D7 after therapeutic plasma exchange. This immunotherapy will be continued per clinician judgment according at 3-week (for pembrolizumab) or 2-week (for nivolumab) intervals as clinically indicated until progression and/or adverse events.

Agent	Dose	Days administered	Pre-medication*
Pembrolizumab (Keytruda®)	200mg IV	D7	None
Nivolumab (Opdivo®)	240mg IV	D7	None

~~7.4.~~7.5. Follow up protocol

~~7.4.1.~~7.5.1. Follow-up imaging

For peer review only

Standard of care CT, PET, and/or MRI scans will be used as clinically indicated and ordered for follow up imaging.

7.5. 7.6. Response assessment

7.5.1. 7.6.1. Post-treatment tumor recurrence will be monitored with follow-up imaging to assess tumor response based on RECIST criteria until progression or death (or up to 2 years)

7.6. 7.7. Outcomes

Patient-centric outcomes measured will include time to progression and time to death if applicable. These will be determined by chart review.

7.7. 7.8. Acute and late toxicity monitoring

Toxicity will be measured by standard-of-care irAE forms. No known side-effect of plasma exchange constitutes an adverse event that is not anticipated by irAE monitoring.

7.8. 7.9. Dose Modification Based on Adverse Event

Dose modification for immunotherapies in future cycles will be at the discretion of the treating physician.

7.9. 7.10. Return to consenting institution if applicable,

For this protocol, the patient must return to the consenting institution for evaluation at least every 182 days during treatment. Local treating physician evaluations, if done according to standard of care, are also acceptable after blood draws have been completed.

For peer review only

8. Dosage Modification Based on Adverse Events

8.1. Initial treatment with TPE carries low but possible risk of hypotension, infection, or excessive bleeding. Should any patient experience an adverse event related to plasma exchange, TPE will be discontinued.

8.2. Dose modification of pembrolizumab or nivolumab will be performed according to clinician preference over the course of the study. These changes are made at the discretion of the treating physician.

For peer review only

9. Ancillary Treatment/Supportive Care

9.1. Antiemetics

Antiemetics may be used at the discretion of the attending physician.

9.2. Premedication for chemotherapy will be given per institutional protocols.

9.3. No biotin-containing compounds will be administered or prescribed.

For peer review only

10. Adverse Event (AE) Monitoring and Reporting

10.1. Definitions

10.1.1. Adverse Event: Any untoward medical occurrence associated with the use of a drug in humans, whether or not considered drug related.

10.1.2. Serious Adverse Event: Adverse events are classified as serious or non-serious. Serious problems/events can be well defined and include:

10.1.2.1. Death

10.1.2.2. Life threatening adverse experience

10.1.2.3. Hospitalization

10.1.2.4. Inpatient, new, or prolonged; disability/incapacity

10.1.2.5. Persistent or significant birth defect/anomaly

10.1.2.6. and/or per protocol may be problems/events that in the option of the sponsor investigator may have adversely affected the rights, safety, or welfare of the subjects or others, or substantially comprised the research data. All adverse events that do not meet any of the criteria for serious, should be regarded as non-serious adverse events.

Unanticipated Problems Involving Risks to Subjects or Others (UPIRTSO)- Any unanticipated problem or adverse event that meets the following three criteria:

- Serious: Serious problems or events that results in significant harm, (which may be physical, psychological, financial, social, economic, or legal) or increased risk for the subject or others (including individuals who are not research subjects). These include: (1) death; (2) life threatening adverse experience; (3) hospitalization - inpatient, new, or prolonged; (4) disability/incapacity - persistent or significant; (5) birth defect/anomaly; (6) breach of confidentiality and (7) other problems, events, or new information (i.e. publications, DSMB reports, interim findings, product labeling change) that in the opinion of the local investigator may adversely affect the rights, safety, or welfare of the subjects or others, or substantially compromise the research data,

  AND

- Unanticipated: (i.e. unexpected) problems or events are those that are not already described as potential risks in the protocol, consent document, not listed in the Investigator's Brochure, or not part of an underlying disease. A problem or event is "unanticipated" when it was unforeseeable at the time of its occurrence. A problem or event is

For peer review only - http://bmjopen.bmj.com/site/about/guidelines.xhtml

"unanticipated" when it occurs at an increased frequency or at an increased severity than expected, AND

- Related: A problem or event is "related" if it is possibly related to the research procedures.
- Preexisting Condition- A preexisting condition is one that is present at the start of the study. A preexisting condition should be recorded as an adverse event if the frequency, intensity, or the character of the condition worsens during the study period. At screening, any clinically significant abnormality should be recorded as a preexisting condition. At the end of the study, any new clinically significant findings/abnormalities that meet the definition of an adverse event must also be recorded and documented as an adverse event.

10.1.3. Suspected Adverse Reaction: Any adverse event for which there is a reasonable possibility that the drug or procedure caused the adverse event.

10.2. Recording Adverse Events/Treatment being studied

CTCAE term (AE description) and grade: The descriptions and grading scales found in the revised NCI Common Terminology Criteria for Adverse Events (CTCAE) version 5.0 will be utilized for AE reporting. All appropriate treatment areas should have access to a copy of the CTCAE version 5.0. A copy of the CTCAE version 5.0 can be downloaded from the CTEP web site: (http://ctep.cancer.gov/protocolDevelopment/electronic_applications/ctc.htm)

10.2.1. Adverse event monitoring and reporting is a routine part of every clinical trial. First, identify and grade the severity of the event using the CTCAE version 5.0. Next, determine whether the event is expected or unexpected and if the adverse event is related to the medical treatment or procedure. With this information, determine whether the event must be reported as an expedited report (see Section 10.25).

Expedited and routine reports are to be completed within the timeframes and via the mechanisms specified in Sections 10.4 and 10.6. All expected AE reports must also be sent to the local Institutional Review Board (IRB) according to local IRB's policies and procedures.

10.2.1.1.1.1. When assessing whether an adverse event (AE) is related to a medical agent(s) medical or procedure, the following attribution categories are utilized:

For peer review only

Definite - The AE is clearly related to the agent(s)/procedure.

Probable - The AE is likely related to the agent(s)/procedure.

Possible - The AE may be related to the agent(s)/procedure.

Unlikely - The AE is doubtfully related to the agent(s)/procedure.

Unrelated - The AE is clearly NOT related to the agent(s)/procedure.

10.2.2. Exceptions to Expedited Reporting For Immunotherapy Related Toxicities

An expedited report may not be required for specific Grade 1, 2 and 3 serious Adverse Events. Any protocol specific reporting procedures MUST BE SPECIFIED BELOW and will supersede the standard Expedited Adverse Event Reporting Requirements**: Hospitalizations for reasons deemed to be disease related will not be reported.**

**CTCAE System Organ Class (SOC)**	**Adverse event/ Symptoms**	**CTCAE Grade at which the event will not be reported in an expedited manner[1]**
General disorders and administrations site conditions	Fatigue	*≤Grade 3*
	Malaise	*≤Grade 3*
Skin and subcutaneous/ Tissue Disorders	Alopecia	*≤Grade 4*

[1] These exceptions only apply if the adverse event does not result in hospitalization. If the adverse event results in hospitalization, then the standard expedited adverse events reporting requirements must be followed.
Specific protocol exceptions to expedited reporting should be reported expeditiously by investigators **ONLY** if they exceed the expected grade of the event.

[1] These exceptions only apply if the adverse event does not result in hospitalization. If the adverse event results in hospitalization, then the standard expedited adverse events reporting requirements must be followed.
Specific protocol exceptions to expedited reporting should be reported expeditiously by investigators **ONLY** if they exceed the expected grade of the event.

For peer review only

10.3. Other Required Reporting

10.3.1. Unanticipated Problems Involving Risks to Subjects or Others (UPIRTSOS)

Unanticipated Problems Involving Risks to Subjects or Others (UPIRTSOS) in general, include any incident, experience, or outcome that meets **all** of the following criteria:

1. Unexpected (in terms of nature, severity, or frequency) given (a) the research procedures that are described in the protocol-related documents, such as the IRB-approved research protocol and informed consent document; and (b) the characteristics of the subject population being studied;
2. Related or possibly related to participation in the research (in this guidance document, possibly related means there is a reasonable possibility that the incident, experience, or outcome may have been caused by the procedures involved in the research); and
3. Suggests that the research places subjects or others at a greater risk of harm (including physical, psychological, economic, or social harm) than was previously known or recognized.

Some unanticipated problems involve social or economic harm instead of the physical or psychological harm associated with adverse events. In other cases, unanticipated problems place subjects or others at increased *risk* of harm, but no harm occurs.

Note: If there is no language in the protocol indicating that pregnancy is not considered an adverse experience for this trial, and if the consent form does not indicate that subjects should not get pregnant/impregnate others, then any pregnancy in a subject/patient or a male patient's partner (spontaneously reported) which occurs during the study or within

10.3.2. Death

Note: A death on study requires both routine and expedited reporting regardless of causality, unless as noted below. Attribution to treatment or other cause must be provided.

Any death occurring within 30 days of the last dose, regardless of attribution to an agent/intervention under an IND/IDE requires expedited reporting within 24-hours.

For peer review only

Any death occurring greater than 30 days with an attribution of possible, probable, or definite to an agent/intervention under an IND/IDE requires expedited reporting within 24-hours.

Reportable categories of Death

- Death attributable to a CTCAE term.
- Death Neonatal: A disorder characterized by cessation of life during the first 28 days of life.
- Death NOS: A cessation of life that cannot be attributed to a CTCAE term associated with Grade 5.
- Sudden death NOS: A sudden (defined as instant or within one hour of the onset of symptoms) or an unobserved cessation of life that cannot be attributed to a CTCAE term associated with Grade 5.
- Death due to progressive disease should be reported as **Grade 5 "Neoplasms benign, malignant and unspecified (including cysts and polyps) – Other (Progressive Disease)"** under the system organ class (SOC) of the same name. Evidence that the death was a manifestation of underlying disease (e.g., radiological changes suggesting tumor growth or progression: clinical deterioration associated with a disease process) should be submitted.

10.4. Required Routine Reporting

10.4.1. Toxicity Evaluations

Pretreatment symptoms/conditions to be graded at baseline and adverse events to be graded at each evaluation.

Grading is per CTCAE v5.0 **unless** alternate grading is indicated in the table below: (this table outlines the AE's you want to have assessed at each study visit the patient has)

CTCAE System/Organ/Class (SOC)	Adverse event/Symptoms	Baseline	Each evaluation
Gastrointestinal	*Diarrhea*	x	x
Dermatologic	*Rashes*	x	x

For peer review only

CTCAE System/Organ/Class (SOC)	Adverse event/Symptoms	Baseline	Each evaluation
Respiratory	*Pneumonitis*	x	x
Adrenal	*Hypophysitis*	x	x
Rheumatologic	*Myalgias, arthralgias*	x	x
Liver	*Hepatitis*	x	x

10.5. Monitoring and Auditing

The investigator will permit study-related monitoring, audits, and inspections by the IRB, the sponsor, and government regulatory agencies, of all study related documents (e.g. source documents, regulatory documents, data collection instruments, study data etc.). The investigator will ensure the capability for inspections of applicable study-related facilities (e.g. pharmacy, diagnostic laboratory, etc.). Participation as an investigator in this study implies acceptance of potential inspection by government regulatory authorities and applicable compliance offices

10.5.1. Medical Monitoring

It is the responsibility of the Principal Investigator to oversee the safety of the study at his/her site. This safety monitoring will include careful assessment and appropriate reporting of adverse events as noted above, as well as the construction and implementation of a site data and safety-monitoring plan (see section 10.5 “Monitoring and Auditing”). Medical monitoring will include a regular assessment of the number and type of serious adverse events. “Any serious adverse events will be followed up by the sentinel event reporting procedure”

10.5.2. Internal Data and Safety Monitoring Board

The trial will be reviewed by the Cancer Center Auditing area on a bi-annual or yearly basis dependent on random study selection to assess accrual, adverse events, and any endpoint problems. Any safety issues requiring protocol changes will be communicated through protocol amendments.

For peer review only

11. Treatment Evaluation/Measurement of Effect

Response and progression will be evaluated in this study using the new international criteria proposed by the revised Response Evaluation Criteria in Solid Tumors (RECIST) guidelines (version 1.1) [18]. Changes in the largest diameter (unidimensional measurement) of the tumor lesions and the short axis measurements in the case of lymph nodes are used in the RECIST guideline.

11.1. Schedule of Evaluations:

11.1.1. For the purposes of this study, patients should be reevaluated every 3-6 months according to clinical judgment.

11.2. Definitions of Measurable and Non-Measurable Disease

11.2.1. Measurable Disease

11.2.1.1. A non-nodal lesion is considered measurable if its longest diameter can be accurately measured as ≥2.0 cm with chest x-ray, or as ≥1.0 cm with CT scan, CT component of a PET/CT, or MRI.

11.2.1.2. A superficial non-nodal lesion is measurable if its longest diameter is ≥ 1.0 cm in diameter as assessed using calipers (e.g. skin nodules) or imaging. In the case of skin lesions, documentation by color photography, including a ruler to estimate the size of the lesion, is recommended.

11.2.1.3. A malignant lymph node is considered measurable if its short axis is >1.5 cm when assessed by CT scan (CT scan slice thickness recommended to be no greater than 5 mm).

NOTE: Tumor lesions in a previously irradiated area are not considered measurable disease.

11.2.2. Non-Measurable Disease

11.2.2.1. All other lesions (or sites of disease) are considered non-measurable disease, including pathological nodes (those with a short axis ≥1.0 to <1.5 cm). Bone lesions, leptomeningeal disease, ascites, pleural/pericardial effusions, lymphangitis cutis/pulmonis, inflammatory breast disease, and abdominal masses (not followed by CT or MRI), are considered as non-measurable as well.

For peer review only

Note: 'Cystic lesions' thought to represent cystic metastases can be considered as measurable lesions, if they meet the definition of measurability described above. However, if non-cystic lesions are present in the same patient, these are preferred for selection as target lesions. In addition, lymph nodes that have a short axis <1.0 cm are considered non-pathological (i.e., normal) and should not be recorded or followed.

11.3. Guidelines for Evaluation of Measurable Disease

11.3.1. Measurement Methods:

- All measurements should be recorded in metric notation (i.e., decimal fractions of centimeters) using a ruler or calipers.
- The same method of assessment and the same technique must be used to characterize each identified and reported lesion at baseline and during follow-up. For patients having only lesions measuring at least 1 cm to less than 2 cm must use CT imaging for both pre- and post-treatment tumor assessments.
- Imaging-based evaluation is preferred to evaluation by clinical examination when both methods have been used at the same evaluation to assess the antitumor effect of a treatment.

11.3.2. Acceptable Modalities for Measurable Disease:

- Conventional CT and MRI: This guideline has defined measurability of lesions on CT scan based on the assumption that CT slice thickness is 5 mm or less. If CT scans have slice thickness greater than 5 mm, the minimum size for a measurable lesion should be twice the slice thickness.

- As with CT, if an MRI is performed, the technical specifications of the scanning sequences used should be optimized for the evaluation of the type and site of disease. The lesions should be measured on the same pulse sequence. Ideally, the same type of scanner should be used and the image acquisition protocol should be followed as closely as possible to prior scans. Body scans should be performed with breath-hold scanning techniques, if possible.

- PET-CT: If the site can document that the CT performed as part of a PET-CT is of identical diagnostic quality to a diagnostic CT (with IV and oral contrast), then the CT portion of the PET-CT can be used for RECIST measurements and can be used interchangeably with conventional CT in accurately measuring cancer lesions over time.

For peer review only

- Chest X-ray: Lesions on chest x-ray are acceptable as measurable lesions when they are clearly defined and surrounded by aerated lung. However, CT scans are preferable.

- Physical Examination: For superficial non-nodal lesions, physical examination is acceptable, but imaging is preferable, if both can be done. In the case of skin lesions, documentation by color photography, including a ruler to estimate the size of the lesion, is recommended.

- FDG-PET: FDG-PET scanning is allowed to complement CT scanning in assessment of progressive disease [PD] and particularly possible 'new' disease. A 'positive' FDG-PET scanned lesion is defined as one which is FDG avid with an update greater than twice that of the surrounding tissue on the attenuation corrected image; otherwise, an FDG-PET scanned lesion is considered 'negative.' New lesions on the basis of FDG-PET imaging can be identified according to the following algorithm:

  a. Negative FDG-PET at baseline with a positive FDG-PET at follow-up is a sign of PD based on a new lesion.

  b. No FDG-PET at baseline and a positive FDG-PET at follow-up:

     i. If the positive FDG-PET at follow-up corresponds to a new site of disease confirmed by CT, this is PD.

     ii. If the positive FDG-PET at follow-up is not confirmed as a new site of disease on CT at the same evaluation, additional follow-up CT scans (i.e., additional follow-up scans at least 4 weeks later) are needed to determine if there is truly progression occurring at that site. In this situation, the date of PD will be the date of the initial abnormal PDG-PET scan.

     iii If the positive FDG-PET at follow-up corresponds to a pre-existing site of disease on CT that is not progressing on the basis of the anatomic images, it is not classified as PD.

11.3.3. Measurement at Follow-up Evaluation:

- In the case of stable disease (SD), follow-up measurements must have met the SD criteria at least once after study entry at a minimum interval of 6 weeks.

- The cytological confirmation of the neoplastic origin of any effusion that appears or worsens during treatment when the measurable tumor has

met criteria for response or stable disease is mandatory to differentiate between response or stable disease (an effusion may be a side effect of the treatment) and progressive disease.

- Cytologic and histologic techniques can be used to differentiate between PR and CR in rare cases (e.g., residual lesions in tumor types such as germ cell tumors, where known residual benign tumors can remain.)

11.4. Measurement of Effect

11.4.1. Target Lesions and Target Lymph Nodes

- Measurable lesions (as defined in Section 11.2.1) up to a maximum of 5 lesions, representative of all involved organs, should be identified as "Target Lesions" and recorded and measured at baseline. These lesions can be non-nodal or nodal (as defined in 11.2.1), where no more than 2 lesions are from the same organ and no more than 2 malignant nodal lesions are selected.

  Note: If fewer than 5 target lesions and target lymph nodes are identified (as there often will be), there is no reason to perform additional studies beyond those specified in the protocol to discover new lesions.

- Target lesions and target lymph nodes should be selected on the basis of their size, be representative of all involved sites of disease, but in addition should be those that lend themselves to reproducible repeated measurements. It may be the case that, on occasion, the largest lesion (or malignant lymph node) does not lend itself to reproducible measurements in which circumstance the next largest lesion (or malignant lymph node) which can be measured reproducibly should be selected.

- Baseline Sum of Dimensions (BSD): A sum of the longest diameter for all target lesions plus the sum of the short axis of all the target lymph nodes will be calculated and reported as the baseline sum of dimensions (BSD). The BSD will be used as reference to further characterize any objective tumor response in the measurable dimension of the disease.

- Post-Baseline Sum of the Dimensions (PBSD): A sum of the longest diameter for all target lesions plus the sum of the short axis of all the target lymph nodes will be calculated and reported as the post-baseline sum of dimensions (PBSD). If the radiologist is able to provide an actual measure for the target lesion (or target lymph node), that should

For peer review only

be recorded, even if it is below 0.5 cm. If the target lesion (or target lymph node) is believed to be present and is faintly seen but too small to measure, a default value of 0.5 cm should be assigned. If it is the opinion of the radiologist that the target lesion or target lymph node has likely disappeared, the measurement should be recorded as 0 cm.

- The minimum sum of the dimensions (MSD) is the minimum of the BSD and the PBSD.

11.4.2. Non-Target Lesions & Non-Target Lymph Nodes

Non-measurable sites of disease (Section 11.2.2) are classified as non-target lesions or non-target lymph nodes and should also be recorded at baseline. These lesions and lymph nodes should be followed in accord with the above.

11.4.3. Response Criteria

11.4.3.1. All target lesions and target lymph nodes followed by CT/MRI/PET-CT/Chest X-ray/physical examination must be measured on re-evaluation at evaluation times specified in Section 11.1. Specifically, a change in objective status to either a PR or CR cannot be done without re-measuring target lesions and target lymph nodes.

Note: Non-target lesions and non-target lymph nodes should be evaluated at each assessment, especially in the case of first response or confirmation of response. In selected circumstances, certain non-target organs may be evaluated less frequently. For example, bone scans may need to be repeated only when complete response is identified in target disease or when progression in bone is suspected.

11.4.3.2. Evaluation of Target Lesions

Complete Response (CR):
All of the following must be true:

a. Disappearance of all target lesions.

b. Each target lymph node must have reduction in short axis to <1.0 cm.

Partial Response (PR):
At least a 30% decrease in PBSD (sum of the longest diameter for all target lesions plus the sum of the

For peer review only

short axis of all the target lymph nodes at current evaluation) taking as reference the BSD (*see* Section 11.4.1).

Progression (PD):

At least one of the following must be true:

a. At least one new malignant lesion, which also includes any lymph node that was normal at baseline (< 1.0 cm short axis) and increased to ≥ 1.0 cm short axis during follow-up.

b. At least a 20% increase in PBSD (sum of the longest diameter for all target lesions plus the sum of the short axis of all the target lymph nodes at current evaluation) taking as reference the MSD (Section 11.4.1). In addition, the PBSD must also demonstrate an absolute increase of at least 0.5 cm from the MSD.

c. See Section 11.32 for details in regards to the requirements for PD via FDG-PET imaging.

Stable Disease (SD):

Neither sufficient shrinkage to qualify for PR, nor sufficient increase to qualify for PD taking as reference the MSD.

11.4.3.3. Evaluation of Non-Target Lesions & Non-Target Lymph Nodes

Complete Response (CR):

All of the following must be true:

a. Disappearance of all non-target lesions.

b. Each non-target lymph node must have a reduction in short axis to <1.0 cm.

Non-CR/Non-PD: Persistence of one or more non-target lesions or non-target lymph nodes.

Progression (PD): At least one of the following must be true:

a. At least one new malignant lesion, which also includes any lymph node that was normal at baseline (< 1.0 cm short axis) and increased to ≥ 1.0 cm short axis during follow-up.

b. Unequivocal progression of existing non-target lesions and non-target lymph nodes. (NOTE: Unequivocal progression should not normally trump target lesion and target lymph node status. It must be representative of overall disease status change.)

c. See Section 11.3.2 for details in regards to the requirements for PD via FDG-PET imaging.

11.4.4. Overall Objective Status

The overall objective status for an evaluation is determined by combining the patient's status on target lesions, target lymph nodes, non-target lesions, non-target lymph nodes, and new disease as defined in the following table:

For Patients with Measurable Disease

Target Lesions & Target Lymph Nodes	Non-Target Lesions & Non-Target Lymph Nodes	New Sites of Disease	Overall Objective Status
CR	CR	No	CR
CR	Non-CR/Non-PD	No	PR
PR	CR<br>Non-CR/Non-PD	No	PR
CR/PR	Not All Evaluated*	No	PR**
SD	CR<br>Non-CR/Non-PD<br>Not All Evaluated*	No	SD
Not all Evaluated	CR<br>Non-CR/Non-PD<br>Not All Evaluated*	No	Not Evaluated (NE)
PD	Unequivocal PD<br>CR<br>Non-CR/Non-PD<br>Not All Evaluated*	Yes or No	PD

For peer review only

**Target Lesions & Target Lymph Nodes**	**Non-Target Lesions & Non-Target Lymph Nodes**	**New Sites of Disease**	**Overall Objective Status**
CR/PR/SD/PD/Not all Evaluated	Unequivocal PD	Yes or No	PD
CR/PR/SD/PD/Not all Evaluated	CR<br>Non-CR/Non-PD<br>Not All Evaluated*	Yes	PD

*See Section 11.4.3.1

** NOTE: This study uses the protocol RECIST v1.1 template dated 2/16/2011. For data collection and analysis purposes the objective status changed from SD to PR in the NCCTG protocol RECIST v1.1 template as of 2/16/2011 and to match RECIST v1.1 requirements.

11.4.5. Symptomatic Deterioration

Patients with global deterioration of health status requiring discontinuation of treatment without objective evidence of disease progression at that time, and not either related to study treatment or other medical conditions, should be reported as PD due to "symptomatic deterioration." Every effort should be made to document the objective progression even after discontinuation of treatment due to symptomatic deterioration. A patient is classified as having PD due to "symptomatic deterioration" if any of the following occur that are not either related to study treatment or other medical conditions:

- Weight loss >10% of body weight.
- Worsening of tumor-related symptoms.
- Decline in performance status of >1 level on ECOG scale.

For peer review only

12. Descriptive Factors

12.1. Prior treatment: 1 vs 2 vs 3+

12.2. Disease stage

12.3. Patient age

12.4. Other medical problems

12.5. Baseline sPD-L1

12.6. Baseline evPD-L1

For peer review only

13. Treatment/Follow–up Decision at Evaluation of Patient

13.1. A patient is deemed *ineligible* if after registration, it is determined that at the time of registration, the patient did not satisfy each and every eligibility criteria for study entry. The patient will go off study.

13.1.1. If the patient received CT, MR, and/or PET for radiotherapy planning, all data up until the point of confirmation of ineligibility must be submitted.

13.1.2. If the patient never received plasma exchange, on-study material must be submitted. No additional follow up is necessary.

13.2. Those patients who will not receive any radiation treatment or who will receive radiation treatment elsewhere will go off study.

13.3. Patients who are CR, PR, REGR, or SD will continue to obtain imaging at each clinically indicated follow up for up to *2 years from registration*.

13.4. Patients who develop PD or withdraw from further follow up assessments will be followed for survival only.

13.5. Inevaluable patients: If a patient fails to complete therapy for reasons other than toxicity, the patient will be regarded as inevaluable and will be replaced.

For peer review only

14. Body Fluid Biospecimens

**Correlative Study (Section 4.0 for more information)**	**Mandatory or Optional**	**Blood or Body Fluid being Collected**	**Type of Collection Tube (color of tube top)**	**Volume to collect per tube (# of tubes to be collected)**	**Visit 1** ***(Screening)***	**Visit 2** ***(Pre-Initial TPE)***	**Visit 3** ***(Post-Final TPE***	**Visit 4** ***(Pre-2nd IO)***	**Process at site? (Yes or No)**	**Temperature Conditions for Storage /Shipping**
Pharmacokinetics, Circulating EVs, PBMCs	Mandatory	Serum	Yellow	6mL (6)	X	X	X	X	No	Ambient
Waste	Mandatory	Plasma exchange waste product	Waste bag	Up to 800cc (1)		X			No	Ambient

For peer review only

14.1. Collection and Processing

Specimens will be collected at the following time points:

- #1 Pre-SBRT (or one week prior to immunotherapy)
- #2 Pre-Initial TPE (for selected patients with sPD-L1 levels above cutoff)
- #3 Post-Final TPE
- #4 2-3 week follow-up at standard clinical appointment prior to next round of immunotherapy

14.1.1. 6-6ML tubes, 36 ml will be collected in citrate yellow-toped tubes at each timepoint. The tubes should be gently inverted 7 to 10 times to thoroughly mix the samples. These samples will be processed within 7 days of collection for sPD-L1, extracellular vesicles, and other metabolites using ELISA and flow cytometry per lab protocols. All other samples must be labeled for processing within one week. Citrate tubes will be provided for this project.

For peer review only

15. 0 Drug Information

15.1. **Pembrolizumab (Keytruda®)** – Standard of care treatment allowed in this study.

15.1.1. Approved for treatment of advanced melanoma (unresectable or metastatic)

15.1.2. Dose 200mg IV every 3 weeks until disease progression or unacceptable toxicity.

15.1.3. Class: Immune checkpoint inhibitor (ICI), monoclonal programmed death receptor-1 (PD-1) blocking human monoclonal antibody.

15.1.4. FDA approval for recurrent/metastatic cervical cancer, advanced endometrial carcinoma, recurrent locally advanced/metastatic esophageal or gastric cancer, unresectable/recurrent/metastatic head and neck squamous cell cancer, advanced hepatocellular carcinoma, relapsed/refractory classical Hodgkin lymphoma, recurrent/metastatic Merkel cell carcinoma, stage III or IV non-small cell lung cancer, relapsed/refractory mediastinal large B-cell lymphoma, advanced renal cell carcinoma, metastatic small cell lung cancer, high-risk locally advanced/metastatic urothelial carcinoma, and unresectable/metastatic microsatellite instability-high cancers of any kind [19].

15.1.5. Adverse events related to immune-related treatments (irAE) will be collected at each follow-up visit. Serious irAE include pneumonitis, colitis, hepatitis, nephritis/renal dysfunction, hypothyroidism/hyperthyroidism.

15.1.6. Pharmacokinetics: administered intravenously, clearance difference not considered to be clinically important.

15.2. **Nivolumab (Opdivo®)**– Standard of care treatment allowed in this study.

15.2.1. Approved for treatment of advanced melanoma (unresectable or metastatic)

15.2.2. Dose 240mg IV every 2 weeks until disease progression or unacceptable toxicity.

15.2.3. Class: Immune checkpoint inhibitor (ICI), monoclonal programmed death receptor-1 (PD-1) blocking human monoclonal antibody.

15.2.4. FDA approval for MSI-high colorectal cancer, recurrent/metastatic head and neck SCC, hepatocellular carcinoma, classical Hodgkin lymphoma,

For peer review only

unresectable/metastatic melanoma, advanced renal cell carcinoma, metastatic small cell lung cancer, and locally advanced/metastatic urothelial carcinoma [20].

15.2.5. Adverse events related to immune-related treatments (irAE) will be collected at each follow-up visit. Serious irAE include pneumonitis, colitis, hepatitis, nephritis/renal dysfunction, hypothyroidism/hyperthyroidism.

15.2.6. Pharmacokinetics: administered intravenously, clearance unchanged by dose. 30-minute infusion comparable to 60-minute infusion. Clearance increased by 20% in the presence of anti-nivolumab antibodies.

For peer review only

16.0 Statistical Considerations and Methodology

16.1 Overview and Study Design:

This is a feasibility trial that seeks to assess feasibility and kinetics of the plasma exchange approach in these patients. In addition, we plan to assess response rate, overall survival, progression-free survival, adverse events, and correlative analysis.

16.1.1 Primary Endpoint

The primary endpoint of this trial is to assess the feasibility and kinetics for this treatment approach. Feasibility will be assessed by being able to complete the study accrual in a reasonable time period, where if we cannot enroll all 20 patients within 2 years, we'll assume this approach isn't feasible. Kinetics of sPD-L1 production in melanoma will be asssessed in an exploratory fashion by assessing the change in the sPD-L1 levels between different time points of interest. Since this feasibility study is small and exploratory, no power calculations are needed. Additional comparator substances or potentially pathogenic tumor substances will also be measured for comparison.

16.1.2 Sample Size

Eighteen eligible patients will be accrued onto this feasibilty study. We anticipate accruing an additional 2 patients to account for ineligibility, cancellation, major treatment violation, or other reasons. Therefore, maximum accrual is expected to be 20 registered patients. To accrue 20 patients that exhibit sPD-L1 above our cutoff (usually ~35% of patients), we expect to pre-register around 60 patients total.

16.1.3 Accrual Time and Study Duration

The anticipated registration rate is approximately 2 patients per month. Therefore, the accrual period for this study is expected to be approximately 10 months. The final analysis can begin approximately 24 months after the trial begins, i.e. as soon as the last patient has been followed for at least 12 months plus time for data entry and clean-up.

16.2 Data and Safety Monitoring

The principal investigator(s) and the study statistician will review the study at least monthly to identify accrual, adverse event, and any endpoint problems that might be developing. The trial is monitored continually by the study team who are notified of every grade 4 and 5 event in real time. The Mayo Clinic Cancer Center (MCCC) Data Safety Monitoring Board (DSMB) is responsible for reviewing accrual and safety data for this trial at least twice a year, based on reports provided by the MCCC Statistical Office. Any safety

For peer review only

issues requiring protocol changes are communicated through protocol amendments.

Adverse Event Stopping Rule: Based on previous experience with this disease, we expect approximately 10% of patients to experience Grade 4+ adverse events. If at any time, 3 of the first 10 registered patients (or 30% or more beyond 10 registered patients) have experienced any Grade 4 or 5 adverse event (at least possibly related to the study treatment), accrual to the study will be suspended to allow for a full review of the data. Each grade 5 event will be reviewed on a case by case basis in a real time fashion to determine whether study accrual should be suspended. After consideration by the study team [ie, Study Chair(s), Statistician, Operations Office, etc] and consultation with representatives at the primary Internal Review Board (IRB) affiliated with the Operations Office, a decision will be made as to whether and how the study will proceed.

16.3. Analysis Plan

16.3.1. Primary Endpoint

For feasibility, we'll just keep track of the accrual and accrual rate. We're hoping for fast accrual, where we can register up to 2 patients per month. Feasibility will be assessed by being able to complete the study accrual in a reasonable time period, where if we can't register all 20 patients within 2 years, we'll assume this approach isn't feasible.

We'll also determine the kinetics of sPD-L1 removal and regeneration by plasma exchange in patients with melanoma. Graphical methods and descriptive statistics will be used to explore this endpoint. The Wilcoxon Signed-Rank test will be used to assess the change in the sPD-L1 levels over time across the different timepoints of interest (pre vs. post initial plasma exchange therapy; post plasma vs. 2 to 3-week follow-up; pre vs. 2 to 3-week follow-up timepoint). The hypothesis is that the sPD-L1 levels will decrease with the use of plasma exchange treatment. The hope is that this decrease will be maintained throughout the first 2 to 3 weeks of follow-up and beyond. Other potentially pathogenic tumor factors will also be measured and compared for correlated studies.

16.3.2. Definition and Analyses of Secondary Endpoints

16.3.2.1. Overall Response rate is the proportion of patients with a tumor response (PR or CR at least 4 weeks apart). All patients meeting the eligibility criteria who have signed a consent form and have begun treatment will be evaluable for the response rate.

For peer review only

16.3.2.2. Progression-free survival (PFS) is defined as the time from registration to the first of either disease progression or death from any cause. Patients who receive the study drug, but then never return for an evaluation will be censored on their last follow-up date. PFS will be estimated using the method of Kaplan-Meier.

16.3.2.3. Overall survival (OS) is defined as the time from registration to death from any cause. OS will be estimated using the method of Kaplan-Meier.

16.3.3. Adverse Events

All patients that have intitiated treatment will be considered evaluable for sadverse event (AE) analyses. The maximum grade for each type of AE will be recorded for each patient, and frequency tables will be reviewed to determine AE patterns.

16.3.4. Analysis of Translational Component

For this correlative analysis, we will determine the effects of plasma exchange on immune cell function, observe the kinetics of extracellular vesicles (EVs) after plasma exchange in patients with melanoma, and associate the kinetics with clinical outcome data (RR, OS, PFS). Associations of categorical data will be assessed using Fisher exact tests. Associations of continuous data with binary data will be assessed using standard Wilcoxon Rank-Sum tests. Assessment of the change in continuous data over time will be done using the Wilcoxon Signed-Rank test. Kaplan-Meier methods and the log-rank test will be used for time-to-event data. This translational study is considered exploratory and hypothesis generating due to the small proposed sample size for this study.

16.4. Inclusion of Women and Minorities

16.4.1. Study availability

This study will be available to all eligible patients, regardless of gender, race or ethnic origin.

16.4.2. Differential effects by race or ethnicity

There is no information currently available regarding differential effects of this regimen in subsets defined by gender, race or ethnicity, and there is no reason to expect such differences to exist. Therefore, although the planned analysis will, as always, look for differences in treatment effect based on racial groupings, the sample size is not increased in order to provide additional power for subset analyses.

16.4.3. Study population

For peer review only

Based on prior studies involving similar disease sites, we expect about 20% of patients will be classified as minorities by race and 5% will be women. Expected sizes (per study design) of racial by gender subsets are shown in the following table:

Accrual Estimates by Gender/Ethnicity/Race

Accrual Targets			
	Sex/Gender		
**Ethnic Category**	**Females**	**Males**	**Total**
Hispanic or Latino	2	2	4
Not Hispanic or Latino	8	8	16
**Ethnic Category: Total of all subjects**	**10**	**10**	**20**
**Racial Category**			
American Indian or Alaskan Native	0	0	0
Asian	1	1	2
Black or African American	1	1	2
Native Hawaiian or other Pacific Islander	0	0	0
White	8	8	16
**Racial Category: Total of all subjects**	**10**	**10**	**20**

Ethnic Categories: **Hispanic or Latino –** a person of Cuban, Mexican, Puerto Rican, South or Central American, or other Spanish culture or origin, regardless of race. The term "Spanish origin" can also be used in addition to "Hispanic or Latino."

Not Hispanic or Latino

Racial Categories: **American Indian or Alaskan Native –** a person having origins in any of the original peoples of North, Central, or South America, and who maintains tribal affiliations or community attachment.

Asian – a person having origins in any of the original peoples of the Far East, Southeast Asia, or the Indian subcontinent including, for example, Cambodia, China, India, Japan, Korea, Malaysia, Pakistan, the Philippine Islands, Thailand, and Vietnam. (Note: Individuals from the Philippine Islands have been recorded as Pacific Islanders in previous data collection strategies.)

Black or African American – a person having origins in any of the black racial groups of Africa. Terms such as "Haitian" or "Negro" can be used in addition to "Black or African American."

Native Hawaiian or other Pacific Islander – a person having origins in any of the original peoples of Hawaii, Guam, Samoa, or other Pacific Islands.

White – a person having origins in any of the original peoples of Europe, the Middle East, or North Africa.

For peer review only

17. Pathology Considerations/Tissue Biospecimens

No tissue Biospecimens.

For peer review only

18. Records and Data Collection Procedures

18.1 Data Handling and Record Keeping

18.1.1 Confidentiality

Information about study subjects will be kept confidential and managed according to the requirements of the Health Insurance Portability and Accountability Act of 1996 (HIPAA). Those regulations require a signed subject authorization informing the subject of the following:

- What protected health information (PHI) will be collected from subjects in this study
- Who will have access to that information and why
- Who will use or disclose that information
- The rights of a research subject to revoke their authorization for use of their PHI.

In the event that a subject revokes authorization to collect or use PHI, the investigator, by regulation, retains the ability to use all information collected prior to the revocation of subject authorization. For subjects that have revoked authorization to collect or use PHI, attempts should be made to obtain permission to collect at least vital status (long term survival status that the subject is alive) at the end of their scheduled study period.

18.1.2 Source Documents

Source data is all information, original records of clinical findings, observations, or other activities in a clinical trial necessary for the reconstruction and evaluation of the trial. Source data are contained in source documents. Examples of these original documents, and data records include: hospital records, clinical and office charts, laboratory notes, memoranda, subjects' diaries or evaluation checklists, pharmacy dispensing records, recorded data from automated instruments, copies or transcriptions certified after verification as being accurate and complete, microfiches, photographic negatives, microfilm or magnetic media, x-rays, subject files, and records kept at the pharmacy, at the laboratories, and at medico-technical departments involved in the clinical trial. Source documents are kept in a secure location that is locked and requires approved access.

18.1.3 Case Report Forms

The study case report form (CRF) is the primary data collection instrument for the study. All data requested on the CRF must be recorded. All missing data must be explained. All data will be entered into electronic case report forms (eCRF's)

For peer review only

through the Medidata Rave system. Case report forms will be automatically rolled out based on a predetermined and visit-based schedule to improve study staff workflow and data quality. Data will be exported nightly to a secure FTP for analysis and reporting.

18.1.4 Records Retention

The investigator will maintain records and essential documents related to the conduct of the study. These will include subject case histories and regulatory documents.

The investigator will retain the specified records and reports as outlined in the Mayo Clinic Research Policy Manual–"Retention of and Access to Research Data Policy" http://mayocontent.mayo.edu/research-policy/MSS_669717

For peer review only

19. Budget

19.1. Costs charged to patient: routine clinical care including SBRT, pembrolizumab or nivolumab depending on physician preference.

19.2. Tests to be research funded: Therapeutic Plasma Exchange (TPE), sPD-L1 ELISA, EV nanoflow, any other tests of potentially pathogenic or comparator substances in blood.

For peer review only

20. References

[1] M. Aitken, M. Kleinrock, A. Simorellis, and D. Nass, "Global Oncology Trends 2018," London, 2018.

[2] A. Haslam and V. Prasad, "Estimation of the Percentage of US Patients With Cancer Who Are Eligible for and Respond to Checkpoint Inhibitor Immunotherapy Drugs," *JAMA Netw. open*, vol. 2, no. 5, p. e192535, May 2019.

[3] J. S. O'Donnell, G. V Long, R. A. Scolyer, M. W. L. Teng, and M. J. Smyth, "Resistance to PD1/PDL1 checkpoint inhibition.," *Cancer Treat. Rev.*, vol. 52, pp. 71–81, Jan. 2017.

[4] J. Zhou *et al.*, "Soluble PD-L1 as a Biomarker in Malignant Melanoma Treated with Checkpoint Blockade.," *Cancer Immunol. Res.*, vol. 5, no. 6, pp. 480–492, 2017.

[5] K. M. Mahoney *et al.*, "A secreted PD-L1 splice variant that covalently dimerizes and mediates immunosuppression," *Cancer Immunol. Immunother.*, pp. 1–12, Dec. 2018.

[6] J. et al Orme, "ADAM10 and ADAM17 cleave PD-L1 to mediate PD-(L)1 inhibitor resistance.," *Oncoimmunology*, vol. In press, p. DOI 10.1080/2162402X.2020.1744980, 2020.

[7] Y. Romero, R. Wise, and A. Zolkiewska, "Proteolytic processing of PD-L1 by ADAM proteases in breast cancer cells," *Cancer Immunol. Immunother.*, vol. 69, no. 1, pp. 43–55, Jan. 2020.

[8] G. Chen *et al.*, "Exosomal PD-L1 contributes to immunosuppression and is associated with anti-PD-1 response," *Nature*, vol. 560, no. 7718, pp. 382–386, Aug. 2018.

[9] M. Poggio *et al.*, "Suppression of Exosomal PD-L1 Induces Systemic Anti-tumor Immunity and Memory," *Cell*, vol. 177, no. 2, pp. 414-427.e13, Apr. 2019.

[10] Y. Li *et al.*, "Serum sPD-1 and sPD-L1 as Biomarkers for Evaluating the Efficacy of Neoadjuvant Chemotherapy in Triple-Negative Breast Cancer Patients.," *Clin. Breast Cancer*, vol. 19, no. 5, pp. 326-332.e1, Oct. 2019.

[11] H. Ha *et al.*, "Soluble programmed death-ligand 1 (sPDL1) and neutrophil-to-lymphocyte ratio (NLR) predicts survival in advanced biliary tract cancer patients treated with palliative chemotherapy.," *Oncotarget*, vol. 7, no. 47, pp. 76604–76612, Nov. 2016.

[12] Y. Fan *et al.*, "Exosomal PD-L1 Retains Immunosuppressive Activity and is Associated with Gastric Cancer Prognosis.," *Ann. Surg. Oncol.*, vol. 26, no. 11, pp. 3745–3755, Oct. 2019.

[13] K. Ando *et al.*, "Plasma Levels of Soluble PD-L1 Correlate With Tumor Regression in Patients With Lung and Gastric Cancer Treated With Immune Checkpoint Inhibitors.," *Anticancer Res.*, vol. 39, no. 9, pp. 5195–5201, Sep. 2019.

[14] A. Costantini *et al.*, "Predictive role of plasmatic biomarkers in advanced non-small cell lung cancer treated by nivolumab.," *Oncoimmunology*, vol. 7, no. 8, p. e1452581, 2018.

[15] Y. Okuma *et al.*, "Soluble Programmed Cell Death Ligand 1 as a Novel Biomarker for Nivolumab Therapy for Non-Small-cell Lung Cancer.," *Clin. Lung Cancer*, vol. 19, no. 5, pp. 410-417.e1, 2018.

[16] J. et al Orme, "Plasma exchange clears soluble PD-L1 and extracellular vesicles in vivo," *Manuscr. Prep.*, 2020.

For peer review only

[17] X. Frigola *et al.*, "Identification of a soluble form of B7-H1 that retains immunosuppressive activity and is associated with aggressive renal cell carcinoma.," *Clin Cancer Res. Clin Cancer Res. April*, vol. 1, no. 177, pp. 1915–1923, 2011.

[18] E. A. Eisenhauer *et al.*, "New response evaluation criteria in solid tumours: Revised RECIST guideline (version 1.1)," *Eur. J. Cancer*, vol. 45, no. 2, pp. 228–247, Jan. 2009.

[19] "Keytruda: Highlights of Presciribing Information," 2017.

[20] "Nivolumab: Highlights of Prescribing Information," 2018.

For peer review only

Appendix I ECOG Performance Status

ECOG PERFORMANCE STATUS*

Grade	ECOG
0	Fully active, able to carry on all pre-disease performance without restriction
1	Restricted in physically strenuous activity but ambulatory and able to carry out work of a light or sedentary nature, e.g., light house work, office work
2	Ambulatory and capable of all selfcare but unable to carry out any work activities. Up and about more than 50% of waking hours
3	Capable of only limited selfcare, confined to bed or chair more than 50% of waking hours.
4	Completely disabled. Cannot carry on any selfcare. Totally confined to bed or chair.
5	Dead

*As published in Am. J. Clin. Oncol.:
Oken, M.M., Creech, R.H., Tormey, D.C., Horton, J., Davis, T.E., McFadden, E.T., Carbone, P.P.: Toxicity And Response Criteria Of The Eastern Cooperative Oncology Group. Am J Clin Oncol 5:649-655, 1982.

The ECOG Performance Status is in the public domain therefore available for public use. To duplicate the scale, please cite the reference above and credit the Eastern Cooperative Oncology Group, Robert Comis M.D., Group Chair.

From http://www.ecog.org/general/perf_stat.html

Adverse Event Form

STUDY NAME

Site Name:____________________________

Pt_ID:____________________________

This form is cumulative and captures adverse events of a single participant throughout the study.

Severity	Study Intervention Relationship	Action Taken Regarding Study Intervention	Outcome of AE	Expected	Serious Adverse Event (SAE)
1 = Mild<br>2 = Moderate<br>3 = Severe<br>4 = Life-Threatening	0 = Not related<br>1 = Unlikely related<br>2 = Possibly related<br>3 = Probably related<br>4 = Definitely related	0 = None<br>1 = Dose modification<br>2 = Medical Intervention<br>3 = Hospitalization<br>4 = Intervention discontinued<br>5 = Other	1 = Resolved<br>2 = Recovered with minor sequelae<br>3 = Recovered with major sequelae<br>4 = Ongoing/Continuing treatment<br>5 = Condition worsening<br>6 = Death<br>7 = Unknown	1 = Yes<br>2 = No	1 = Yes<br>2 = No<br>(if yes, complete SAE form)

At end of study only: Check this box if participant had no adverse events ☐ None

Adverse Event	Start Date	Stop Date	Severity	Relationship	Action Taken	Outcome of AE	Expected?	SAE?

Page __ of __

Adverse Event Form Version 2.0

BMJ Open

Rescuing Cancer Immunity by Plasma Exchange in Metastatic Melanoma (ReCIPE-M1): Protocol for a single-institution, open-label safety trial of plasma exchange to clear sPD-L1 for immunotherapy

Journal:	*BMJ Open*
Manuscript ID	bmjopen-2021-050112.R1
Article Type:	Protocol
Date Submitted by the Author:	27-Dec-2021

Complete List of Authors:	Davidson, Tara M.; Mayo Clinic, Department of Internal Medicine<br>Foster, Nathan; Mayo Clinic, Department of Quantitative Health Sciences<br>Lucien, Fabrice; Mayo Clinic, Department of Urology<br>Svetomir, Markovic; Mayo Clinic, Division of Medical Oncology<br>Dong, Haidong; Mayo Clinic, Department of Urology<br>Winters, Jeffrey L.; Mayo Clinic, Department of Pathology and Laboratory Medicine<br>Park, Sean S.; Mayo Clinic, Radiation Oncology<br>Orme, Jacob; Mayo Clinic, Division of Medical Oncology

**Primary Subject Heading**:	Oncology
Secondary Subject Heading:	Oncology, Urology
Keywords:	ONCOLOGY, IMMUNOLOGY, Radiobiology < RADIOLOGY & IMAGING, Prostate disease < UROLOGY

SCHOLARONE™
Manuscripts

Clinical trial design: BMJ Open

Title: Rescuing Cancer Immunity by Plasma Exchange in Metastatic Melanoma (ReCIPE-M1): Protocol for a single-institution, open-label safety trial of plasma exchange to clear sPD-L1 for immunotherapy

Authors: Tara M. Davidson[1]; Nathan Foster[2]; Fabrice Lucien[3]; Svetomir Markovic[4]; Haidong Dong[3]; Jeffrey L. Winters[5]; Sean S. Park[6,*]; Jacob J. Orme[4,*]

[1]Department of Internal Medicine, Mayo Clinic, Rochester, MN

[2]Department of Quantitative Health Sciences, Mayo Clinic, Rochester, MN

[3]Department of Urology, Mayo Clinic, Rochester, MN

[4]Division of Medical Oncology, Mayo Clinic, Rochester, MN

[5]Department of Pathology and Laboratory Medicine, Mayo Clinic, Rochester, MN

[6]Department of Radiation Oncology, Mayo Clinic, Rochester, MN

*These authors contributed equally

For peer review only

Running title: TPE for melanoma immunotherapy

Keywords: PD-L1, PD-1, soluble PD-L1, sPD-L1

Financial support: Support: NCI CA259236, Lawrence W. and Marilyn W. Matteson Award

For peer review only - http://bmjopen.bmj.com/site/about/guidelines.xhtml

COI: Intellectual property has been filed addressing discoveries disclosed in this manuscript. The authors report no other relevant conflicts of interest.

Corresponding Author:

Jacob Orme, M.D. Ph.D.

200 1st St NW

Rochester, MN 55902

Phone: 507-284-2511

Email: orme.jacob@mayo.edu

Word count: 2,117

Total figures and tables: 2 figures, 1 table

For peer review only

For peer review only - http://bmjopen.bmj.com/site/about/guidelines.xhtml

ABSTRACT

Background: Patients with metastatic melanoma rely on PD-(L)1 immunotherapy, but only one third of patients experience treatment response and all initial responders eventually develop resistance. Tumor-derived extracellular vesicles expressing PD-L1 (evPD-L1) and soluble PD-L1 (sPD-L1) in peripheral blood of patients with melanoma limit PD-(L)1 immunotherapy and correlate with poor survival. Therapeutic plasma exchange (TPE) removes immunosuppressive evPD-L1 and sPD-L1. We hypothesize that TPE may rescue and restore anti-melanoma immunity.

Methods: In this two-arm study (NCT04581382), 60 patients with metastatic melanoma progressing on checkpoint inhibition will be accrued. All patients will receive radiotherapy on days 1-5 (at least one measurable lesion will not be irradiated) and ongoing checkpoint inhibition on day 8 and every 2-3 weeks per standard of care. Patients with baseline sPD-L1 level ≥1.7 ng/ml and adequate clinical capacity will be enrolled in the TPE Intervention Arm and receive TPE on days 5-7 in addition to standard of care radiotherapy and immunotherapy. Other patients will remain in the Standard of Care Arm.

The primary endpoint of the study is to evaluate safety. Secondary endpoints include kinetics of sPD-L1 and evPD-L1 and clinical response by RECIST criteria.

Ethics and Dissemination: This trial has been approved by the Mayo Clinic IRB. It will assess the safety and feasibility of TPE in improving outcomes for PD-(L)1 inhibitor immunotherapy in melanoma. Data will be maintained on a secure database with de-identified patient information. Data will be shared on publication in a peer-reviewed

For peer review only

journal without the aid of professional writers. If successful, this trial will lay the ground for Phase II studies that will include cancer treated with PD-(L)1 inhibitors which may benefit from TPE such as renal, bladder, and lung cancers.

ARTICLE SUMMARY

Strengths and limitations of this study:

- Screens 60 patients with metastatic melanoma progressing despite checkpoint inhibition
- Evaluates the safety of TPE and kinetics of immunomodulatory molecules removed by TPE in vivo in melanoma
- Involves no randomization to intervention versus control arm in this early study

For peer review only

1. INTRODUCTION

The first immunotherapy with interferon was approved by the Food and Drug Administration (FDA) in 1986.[1] Since then, leveraging the body's own immune system to target and kill malignant cells has become the fastest-growing therapeutic approach to cancer worldwide.[2] While early immunotherapies focused on providing immunostimulatory cytokines (*e.g.,* pegilodecakin), more recent approaches have leveraged vaccination (*e.g.,* MAGE-A3), transplant of primed anti-tumor CD8+ T cells (*i.e.,* CAR-T therapy), and inhibiting immune checkpoint signals (*e.g.,* Programmed Death 1 [PD-1] and Programmed Death Ligand 1 [PD-L1] inhibitors like pembrolizumab, nivolumab, and atezolizumab).[3]

The latter approach with PD-(L)1 inhibitors has shown spectacular results in many indications, leading to first-line approvals of checkpoint inhibitor immunotherapy in renal cell carcinoma, non-small cell lung cancer, melanoma, and others.[4-6] Unfortunately, even in these highly promising settings less than half of patients have objective response to PD-(L)1 inhibitor therapy alone.[7] For instance, only about one in three melanomas respond to PD-(L)1 inhibitor pembrolizumab.[6] Intensive research is focused on identifying causes for intrinsic resistance to PD-(L)1 inhibitors.

PD-L1 is classically thought to be a membrane-bound ligand. Recently, however, three additional functional forms of *extracellular* PD-L1 have been identified. Soluble PD-L1 can be produced by transcribed PD-L1 splice variants, of which four variants have been identified in melanoma.[8,9] Metalloproteases ADAM10 and ADAM17 can also cleave the PD-L1 ectodomain from tumor cells and extracellular vesicles (EVs) to form sPD-L1.[10] Lastly, tumor cells release extracellular vesicles bearing intact PD-L1 (evPD-L1).[11]

For peer review only - http://bmjopen.bmj.com/site/about/guidelines.xhtml

sPD-L1 levels are significantly higher in patients with metastatic melanoma at baseline compared to healthy volunteers and elevated levels predict inferior outcomes.[8,10,12] This implies that the same inflammatory/immune response that is known to play a role in PD-L1 expression also increases the rate of extracellular PD-L1 production. In one study, PD-L1 cell surface expression paralleled sPD-L1 secretion in response to cytokines in melanoma cells.[8] Some cancer cells secrete a majority of their PD-L1 on exosomes, even with only minimal amounts of cellular PD-L1 found.[9,11] The leading hypothesis is that the tumor microenvironment, including non-neoplastic cells, may also be contributing to the production of sPD-L1 and evPD-L1. And although radiotherapy has been proposed as a method to induce anti-tumor immunity, studies have also shown that radiation increases the production of extracellular PD-L1 through inflammatory cytokines and other mechanisms.[13,14]

While the roles of these extracellular PD-L1 forms continue to be elucidated, the results of sPD-L1 and evPD-L1 signaling are clear from both in vitro and in vivo studies: sPD-L1 and evPD-L1 outcompete PD-(L)1 inhibitors, initiate apoptosis in CD8+ T cells, and cause systemic immunosuppression by limiting the ability of healthy peripheral blood mononuclear cells to kill tumor cells (Figure 1A). [9,10,15] Studies in multiple malignancies find a negative relationship between high sPD-L1 and outcomes.[16,17,18, 19-27] In melanoma, Zhou et al showed that high pretreatment levels of sPD-L1 were associated with increased likelihood of progressive disease in patients with melanoma treated by CTLA-4 or PD-1 blockade.[8]

We recently explored the potential of therapeutic plasma exchange (TPE) in removing sPD-L1 and evPD-L1.[28] In brief, TPE is a procedure for passing blood through an

For peer review only - http://bmjopen.bmj.com/site/about/guidelines.xhtml

apheresis machine to separate plasma from cellular components and is commonly used clinically for removal of deleterious antibodies.[29] This has been described as a method to “clean” or “flush out” the blood. Plasma containing the offending substance is replaced in this procedure with up to 30% crystalloid in the form of normal saline and either donor plasma or 5% human albumin derived from plasma donation. In our previous report, each session of TPE reduced sPD-L1 and evPD-L1 by 70.8% and 73.1%, respectively.[28] This represents the first clinical intervention to remove either of these substances from circulation in vivo. It is hypothesized that the removal of these extracellular forms of PD-L1 may re-sensitize tumors to immune checkpoint inhibitor treatment (Figure 1B).

Despite these important discoveries, several crucial questions remain unanswered. First, while TPE is commonly prescribed in the outpatient setting, it has not been studied specifically in cancer treated with immunotherapy. Second, the kinetics of sPD-L1 and evPD-L1 in patients with melanoma have not previously been determined. Pilot studies to establish the safety profile and durability of TPE-mediated changes in melanoma are warranted.

2. METHODS

2.1. Overview of study design

This feasibility trial (NCT04581382) will assess (1) the safety of TPE through adverse events monitoring at 1 and 6 months post-therapy (primary outcome), (2) the kinetics of evPD-L1, sPD-L1, and related immunosuppressive metabolites immediately and 2-3

For peer review only

weeks after TPE, and (3) the relative response of patients to combined therapy through response rate, overall survival, and progression-free survival.

2.2. Recruitment, screening procedures and treatment arms

Patients with metastatic melanoma progressing on standard of care on any PD-(L)1 inhibitor are eligible for this study (Table 1, Figure 2). Potentially eligible patients will be approached by the clinical trial coordinator and provide informed consent prior to a baseline screening blood draw. 60 patients will be screened from from December 2020 through October 2023 with an accrual goal of 20 patients to the TPE Intervention Arm. Inclusion criteria comprise age (18 or older), histologically confirmed melanoma, measurable disease per RECIST criteria, ECOG status of three or better, sPD-L1 levels 1.7ng/ml or greater, and feasible vascular access. Exclusion criteria include pregnancy or nursing. Also excluded are those who consume biotin supplementation as it interferes with accurate measurement of sPD-L1.

Enrolled patients with sPD-L1 level at or above 1.7ng/ml will be enrolled in the Intervention Arm where clinical availability of the procedure allows. Other patients will be enrolled in the Standard of Care Arm. All data will be collected in a deidentified fashion in a password-protected database.

2.3. Study interventions

All patients will receive treatment with any PD-(L)1 inhibitor per clinician preference, including but not limited to the continuation of the existing checkpoint inhibitor, escalation to ipilimumab and nivolumab, or switching to an alternative checkpoint inhibitor. Other concomitant therapies will be allowed on the trial. Additional treatment

For peer review only

For peer review only - http://bmjopen.bmj.com/site/about/guidelines.xhtml

comprises radiotherapy on days 1-5 and PD-(L)1 inhibitor treatment on day 8 and every 2-3 weeks thereafter as clinically indicated. Preferred targets for radiotherapy will be symptomatic lesions or lesions that can cause pain, fracture, or other symptoms in the near future. Patients in the Intervention Arm, in addition to the above, will undergo serial blood draws prior to the first TPE session, after the last TPE session and just prior to the next dose of immunotherapy. We will assess the kinetics of sPD-L1 and evPD-L1 at these timepoints.

TPE will be performed using centrifugation-based cell separators, either the Fenwal Amicus (Fresenius KABI USA LLC, Lake Zurich IL, USA) or the Spectra Optia (Terumo BCT Inc, Lakewood CO, USA). For each patient, a single plasma volume was exchanged using either peripheral IVs (preferred) or central lines for vascular access if a central line is present. Due to the possibility of sPD-L1 or PD-L1-positive EVs present in donor plasma, donor plasma (*i.e.,* fresh frozen plasma, FFP) will not be used as a replacement fluid. Rather, 5% normal serum human albumin with up to 500 ml of normal saline will be used as the replacement. The volume used is determined by the device based upon patient gender, height, weight, and hematocrit. Anticoagulation will consist of either 500 ml of acid citrate dextrose solution A (ACD-A) or 500 ml of ACD-A with 5000 units of unfractionated heparin. Anticoagulant to blood ratios will be 1:13 when ACD-A is used and 1:26 when ACD-A/Heparin is used. Patients will not receive routine electrolyte replacement but 10 ml of 10% calcium gluconate will be administered by slow IV push for signs and symptoms of hypocalcemia related to the ACD-A anticoagulant.

For peer review only - http://bmjopen.bmj.com/site/about/guidelines.xhtml

Toxicity assessment using CTCAE version 5.0 will be assessed within 7 days prior to registration as baseline in addition to daily during TPE and 2 weeks post first dose of immunotherapy if receiving nivolumab or 3 weeks post first dose of immunotherapy if receiving pembrolizumab as well as 3 months post-TPE. Safety will also be assessed during any clinically indicated follow-up until progressive disease, death or up to 2 years after registration.

Imaging will be obtained as standard of care and is not stipulated by the study. CT, PET, and/or MRI scans can be used as clinically indicated. Imaging to assess tumor response will be based on RECIST/PERCIST criteria (v1.1) until progression, death or up to 2 years after registration. At least one metastatic lesion will not be radiated to assess response per RECIST and/or PERCIST criteria for those in the Intervention Arm. All oligo-progressive lesions will be radiated for patients in the Standard of Care Arm while on standard-of-care immunotherapy. As patients who progress on immunotherapy are enrolled in this trial, partial or complete response of > 20% will be considered as a positive signal to proceed to a phase II trial. Study materials and procedures were approved by the Mayo Clinic IRB (approval #19-008055).

2.4 Measuring sPD-L1 and evPD-L1 levels

For measurements of sPD-L1, ELISA will be performed as previously published.[18] Both secreted splice variant and shed sPD-L1 are reliably detected by this ELISA. In brief, paired mouse IgG2 monoclonal antibody clones H1A and B11 against extracellular human PD-L1 will be utilized in a capture-detection plate assay using a standard biotinylated antibody and streptavidin-HRP detection method. This assay is specific for sPD-L1 and does not exhibit cross reactivity to other B7-H homologues, nor to evPD-L1.

For peer review only - http://bmjopen.bmj.com/site/about/guidelines.xhtml

Concentrations are determined by optical density measurements along a known standard curve of recombinant human PD-L1. ELISAs are performed by technicians blinded to the identity of the samples.

For measurements of evPD-L1, flow cytometry will be used as previously published.[30] In brief, plasma samples are centrifuged twice at 2000g to deplete platelets. Resultant platelet-free plasma is analyzed using an A60-Micro Plus Nanoscale Flow Cytometer (Apogee FlowSystems) gating for mid-intensity light angle light scatter and markers of interest. Anti-PD-L1 (Genentech: atezolizumab), antibodies are conjugated to fluorophores (Life Technologies: Alexa-647, PE phycoerythrin, and Alexa-488) and titrated prior to use. Nanoscale flow cytometer calibration is performed using a standard reference bead mix as previously published. Flow cytometry is performed by technicians blinded to the identity of the samples. Wilcoxon Signed-Rank test will be used to assess changes in sPD-L1 and evPD-L1 levels over time across the different timepoints of interest.

2.4. Endpoints

The primary endpoint of this trial is to assess the safety of TPE in patients with melanoma receiving PD-(L)1 immunotherapy. Adverse events will be collected according to CTCAE 5.0, and the protocol dictates that if three of the first ten patients experiences grade 4 or greater adverse events the study will be halted. The secondary endpoints of this trial are (i) the kinetics of sPD-L1 and evPD-L1 removal in patients with melanoma receiving PD-(L)1 immunotherapy, (ii) overall response rate as a proportion of patients with a tumor response of PR or CR at least 4 weeks apart, and (iii) progression-free and overall survival.

For peer review only - http://bmjopen.bmj.com/site/about/guidelines.xhtml

2.5. Data collection schedule

The principal investigator and study statistician will review the study at least monthly to identify accrual, adverse events, and any endpoint problems that might be developing. The trial is monitored continually by the study team who are notified of every grade 4 or 5 event in real time.

2.6. Patient and Public Involvement

No patient involved.

3. ETHICS AND DISSEMINATION

We hypothesize that TPE will reliably and safely reduce the levels of sPD-L1 and evPD-L1 in our study patients. We hypothesize that this will alleviate the immunosuppressive microenvironment caused by extracellular PD-L1 as well as minimize any competition for PD-(L)1 inhibitors prior to these study patients receiving their dose of PD-(L)1 inhibitor therapy. We hope to see improved response to these agents through this intervention. If successful, we will explore this further in other neoplasms in Phase II trials. While TPE does carry risks as an invasive procedure (including hypotension, infection and bleeding) and relies on adequate vascular access, it is not restricted to any particular tumor type and is widely available.

This trial has been approved by the Mayo Clinic IRB. Data will be maintained on a secure database with de-identified patient information. Data will be shared on publication in a peer-reviewed journal without the aid of professional writers. If successful, this trial will lay the ground for Phase II studies that will include cancer

For peer review only - http://bmjopen.bmj.com/site/about/guidelines.xhtml

treated with PD-(L)1 inhibitors which may benefit from TPE such as renal, bladder, and lung cancers.

Any modifications to the protocol which may impact on the conduct of the study, potential benefit of the patient or may affect patient safety, including changes of study objectives, study design, patient population, sample sizes, study procedures, or significant administrative aspects will require a formal amendment to the protocol. Such amendment will be approved by the Mayo Clinic IRB prior to implementation and notified to the health authorities in accordance with local regulations.

REFERENCES

1. Golomb HM, Jacobs A, Fefer A, et al. Alpha-2 interferon therapy of hairy-cell leukemia: a multicenter study of 64 patients. *J Clin Oncol.* 1986;4(6):900-905.
2. Dighe S. Immune Checkpoint Inhibitors (ICI) Market by Type (CTLA-4 Inhibitor, PD-1 Inhibitor, and PD-L1 Inhibitor) and Application (Lung Cancer, Bladder Cancer, Melanoma, Hodgkin Lymphoma, and Others). *https://wwwalliedmarketresearchcom/immune-check-point-inhibitors-market (2018).*
3. Dong H, Strome SE, Salomao DR, et al. Tumor-associated B7-H1 promotes T-cell apoptosis: a potential mechanism of immune evasion. *Nat Med.* 2002;8(8):793-800.
4. Hellmann MD, Paz-Ares L, Bernabe Caro R, et al. Nivolumab plus Ipilimumab in Advanced Non-Small-Cell Lung Cancer. *N Engl J Med.* 2019;381(21):2020-2031.
5. Rini BI, Plimack ER, Stus V, et al. Pembrolizumab plus Axitinib versus Sunitinib for Advanced Renal-Cell Carcinoma. *N Engl J Med.* 2019;380(12):1116-1127.
6. Robert C, Schachter J, Long GV, et al. Pembrolizumab versus Ipilimumab in Advanced Melanoma. *N Engl J Med.* 2015;372(26):2521-2532.
7. Haslam A, Prasad V. Estimation of the Percentage of US Patients With Cancer Who Are Eligible for and Respond to Checkpoint Inhibitor Immunotherapy Drugs. *JAMA Netw Open.* 2019;2(5):e192535.
8. Zhou J, Mahoney KM, Giobbie-Hurder A, et al. Soluble PD-L1 as a Biomarker in Malignant Melanoma Treated with Checkpoint Blockade. *Cancer Immunol Res.* 2017;5(6):480-492.
9. Poggio M, Hu T, Pai CC, et al. Suppression of Exosomal PD-L1 Induces Systemic Anti-tumor Immunity and Memory. *Cell.* 2019;177(2):414-427 e413.

For peer review only - http://bmjopen.bmj.com/site/about/guidelines.xhtml

10. Orme JJ, Jazieh KA, Xie T, et al. ADAM10 and ADAM17 cleave PD-L1 to mediate PD-(L)1 inhibitor resistance. *Oncoimmunology.* 2020;9(1):1744980.
11. Chen G, Huang AC, Zhang W, et al. Exosomal PD-L1 contributes to immunosuppression and is associated with anti-PD-1 response. *Nature.* 2018;560(7718):382-386.
12. Chen Y, Wang Q, Shi B, et al. Development of a sandwich ELISA for evaluating soluble PD-L1 (CD274) in human sera of different ages as well as supernatants of PD-L1+ cell lines. *Cytokine.* 2011;56(2):231-238
13. Ding XC, Wang LL, Zhu YF, et al. The Change of Soluble Programmed Cell Death-Ligand 1 in Glioma Patients Receiving Radiotherapy and Its Impact on Clinical Outcomes. *Front Immunol.* 2020;11:580335.
14. Kim HJ, Park S, Kim KJ, Seong J. Clinical significance of soluble programmed cell death ligand-1 (sPD-L1) in hepatocellular carcinoma patients treated with radiotherapy. *Radiother Oncol.* 2018;129(1):130-135.

15. Parry RV, Chemnitz JM, Frauwirth KA, et al. CTLA-4 and PD-1 receptors inhibit T-cell activation by distinct mechanisms. *Mol Cell Biol.* 2005;25(21):9543-9553.

16. Dronca RS, Harrington S, Jegapragasan M, et al. Association of soluble PD-L1 (sPD-L1) with decreased survival in metastatic melanoma. *Journal of Clinical Oncology.* 2017;35(7_suppl):4-4.
17. Dronca RS, Mansfield AS, Liu X, et al. Bim and soluble PD-L1 (sPD-L1) as predictive biomarkers of response to anti-PD-1 therapy in patients with melanoma and lung carcinoma. *Journal of Clinical Oncology.* 2017;35(15_suppl):11534-11534.
18. Frigola X, Inman BA, Lohse CM, et al. Identification of a Soluble Form of B7-H1 That Retains Immunosuppressive Activity and Is Associated with Aggressive Renal Cell Carcinoma. *Clinical Cancer Research.* 2011;17(7):1915-1923.
19. Costantini A, Julie C, Dumenil C, et al. Predictive role of plasmatic biomarkers in advanced non-small cell lung cancer treated by nivolumab. *Oncoimmunology.* 2018;7(8):e1452581.
20. Finkelmeier F, Canli Ö, Tal A, et al. High levels of the soluble programmed death-ligand (sPD-L1) identify hepatocellular carcinoma patients with a poor prognosis. *Eur J Cancer.* 2016;59:152-159.
21. Ha H, Nam AR, Bang JH, et al. Soluble programmed death-ligand 1 (sPDL1) and neutrophil-to-lymphocyte ratio (NLR) predicts survival in advanced biliary tract cancer patients treated with palliative chemotherapy. *Oncotarget.* 2016;7(47):76604-76612.
22. Okuma Y, Hosomi Y, Nakahara Y, Watanabe K, Sagawa Y, Homma S. High plasma levels of soluble programmed cell death ligand 1 are prognostic for reduced survival in advanced lung cancer. *Lung Cancer.* 2017;104:1-6.
23. Sideras K, de Man RA, Harrington SM, et al. Circulating levels of PD-L1 and Galectin-9 are associated with patient survival in surgically treated Hepatocellular Carcinoma independent of their intra-tumoral expression levels. *Scientific Reports.* 2019;9(1):10677.
24. Wang H, Wang L, Liu WJ, et al. High post-treatment serum levels of soluble programmed cell death ligand 1 predict early relapse and poor prognosis in extranodal NK/T cell lymphoma patients. *Oncotarget.* 2016;7(22):33035-33045.

For peer review only - http://bmjopen.bmj.com/site/about/guidelines.xhtml

25. Wang L, Wang H, Chen H, et al. Serum levels of soluble programmed death ligand 1 predict treatment response and progression free survival in multiple myeloma. *Oncotarget.* 2015;6(38):41228-41236.
26. Zhang J, Gao J, Li Y, et al. Circulating PD-L1 in NSCLC patients and the correlation between the level of PD-L1 expression and the clinical characteristics. *Thorac Cancer.* 2015;6(4):534-538.
27. Zheng Z, Bu Z, Liu X, et al. Level of circulating PD-L1 expression in patients with advanced gastric cancer and its clinical implications. *Chin J Cancer Res.* 2014;26(1):104-111.
28. Orme JJ, Enninga EAL, Lucien-Matteoni F, et al. Therapeutic plasma exchange clears circulating soluble PD-L1 and PD-L1-positive extracellular vesicles. *J Immunother Cancer.* 2020;8(2).
29. Padmanabhan A, Connelly-Smith L, Aqui N, et al. Guidelines on the Use of Therapeutic Apheresis in Clinical Practice - Evidence-Based Approach from the Writing Committee of the American Society for Apheresis: The Eighth Special Issue. *J Clin Apher.* 2019;34(3):171-354.
30. Gomes J, Lucien F, Cooper TT, et al. Analytical Considerations in Nanoscale Flow Cytometry of Extracellular Vesicles to Achieve Data Linearity. *Thromb Haemost.* 2018;118(9):1612-1624

For peer review only

ACKNOWLEDGMENTS

ReCIPE-M1 is supported by grants from the Lawrence W. and Marilyn W. Matteson Fund at Mayo Clinic and R21 CA259236-01 from the National Cancer Institute. Some images were created with BioRender.

DECLARATION OF INTEREST STATEMENT

Intellectual property has been filed addressing discoveries disclosed in this manuscript.

The authors report no other relevant conflicts of interest.

For peer review only - http://bmjopen.bmj.com/site/about/guidelines.xhtml

TABLES

Table 1.

INCLUSION CRITERIA		ARM ASSIGNMENTS	
**Inclusion Criteria**	**Exclusion Criteria**		
≥18 years<br>Histologic melanoma progressing despite ICI<br>Measurable disease<br>ECOG ≤ 3<br>Ability to consent	Biotin supplement use<br>Pregnant or nursing	**Intervention Arm**	sPD-L1 ≥1.7 ng/ml *AND* available clinical capacity
		**Standard of Care Arm**	sPD-L1 <1.7 ng/ml *OR* unavailable clinical capacity

Table 1. Study inclusion and exclusion criteria. Eligible patients for the study will be consenting adults with histologically confirmed melanoma receiving PD-(L)1 immunotherapy who are referred for stereotactic body radiation therapy (SBRT). Patients who are receiving biotin supplements, are pregnant, or are nursing are excluded. Patients with elevated sPD-L1 on registration will enter the Intervention Arm where clinical capacity is sufficient. Patients with low sPD-L1 or who enter the trial when clinical capacity is not available will enter the Standard of Care Arm.

For peer review only - http://bmjopen.bmj.com/site/about/guidelines.xhtml

FIGURE LEGENDS

Figure 1. Extracellular PD-L1 Outcompetes Immune Checkpoint Inhibitors to Block Anti-tumor Immunity. (A) While PD-(L)1 inhibitors prevent tumor cell downregulation of T cells, tumor cells secrete evPD-L1 and sPD-L1 (either cleaved by ADAM10/ADAM17 or as splice variants) that outcompete PD-(L)1 inhibitors and downregulate systemic anti-cancer immunity. **(B)** Therapeutic plasma exchange clears extracellular PD-L1 and may re-sensitize tumors to immune checkpoint inhibitor therapy.

Figure 2. Study design in two arms. In brief, eligible patients undergo a registration blood draw and vascular access evaluation to determine appropriate study arm. Patients with elevated baseline sPD-L1 (≥1.7 ng/ml) will enter the Intervention Arm if there is adequate clinical capacity. Other patients will enter the Standard of Care Arm.

For peer review only

For peer review only - http://bmjopen.bmj.com/site/about/guidelines.xhtml

AUTHOR STATEMENT

TD wrote the manuscript. JJO developed hypotheses and concepts, performed initial studies, generated figures and helped write the manuscript. NF performed statistical design. HD, SSP and JLW provided mentorship, equipment, facilities, edits and funding. FL and SM provided additional logistical input, planning and edits. Sponsors and funders have had and will have NO role in study design; collection, management, analysis, or interpretation of data; writing of any report; nor the decision to submit any report for publication. Data will be shared on publication in a peer-reviewed journal without the aid of professional writers. One or more figures was produced using BioRender®.

BIOLOGICAL SPECIMENS

Laboratory specimens (blood draws) will be processed for plasma and peripheral blood mononuclear cells (PBMCs). They will be stored in study identity-labeled tubes at -80C until analysis by flow cytometry, ELISA, or other means for future studies.

DATA SHARING STATEMENT

Deidentified patient information will be made available in a public data repository on publication.

ETHICAL APPROVAL STATEMENT

Study materials and procedures were approved by the Mayo Clinic IRB (approval #19-008055).

For peer review only - http://bmjopen.bmj.com/site/about/guidelines.xhtml

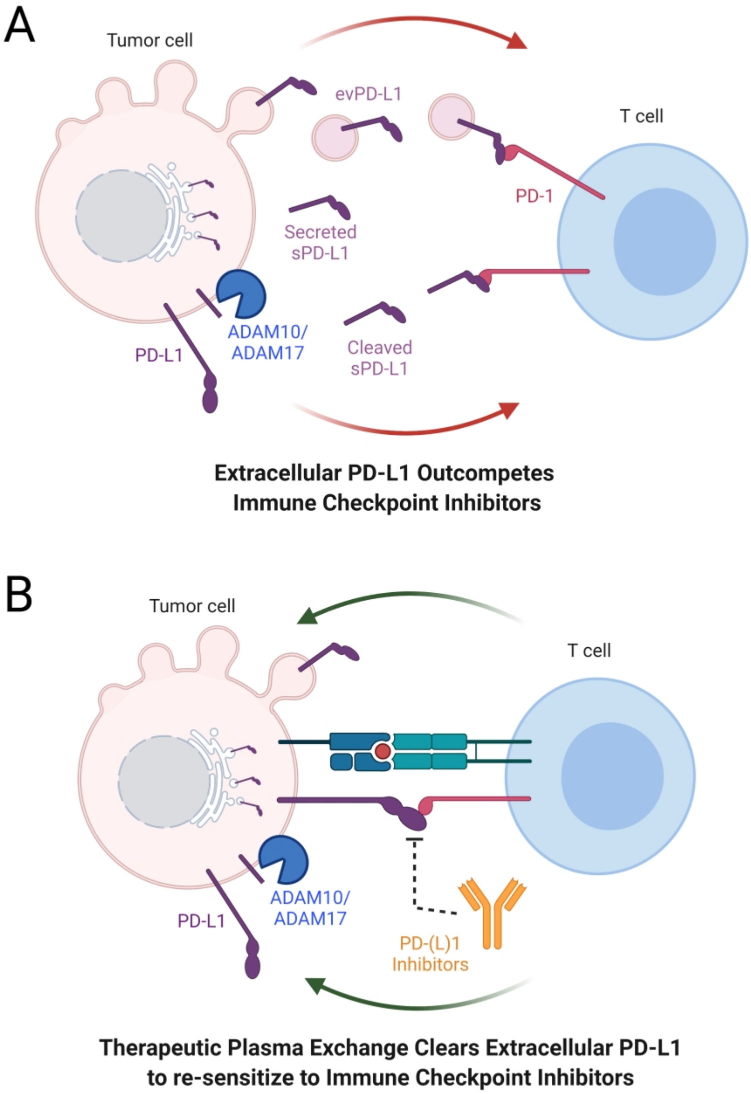

Figure 1. Extracellular PD-L1 Outcompetes Immune Checkpoint Inhibitors to Block Anti-tumor Immunity. (A) While PD-(L)1 inhibitors prevent tumor cell downregulation of T cells, tumor cells secrete evPD-L1 and sPD-L1 (either cleaved by ADAM10/ADAM17 or as splice variants) that outcompete PD-(L)1 inhibitors and downregulate systemic anti-cancer immunity. (B) Therapeutic plasma exchange clears extracellular PD-L1 and may re-sensitize tumors to immune checkpoint inhibitor therapy.

254x354mm (300 x 300 DPI)

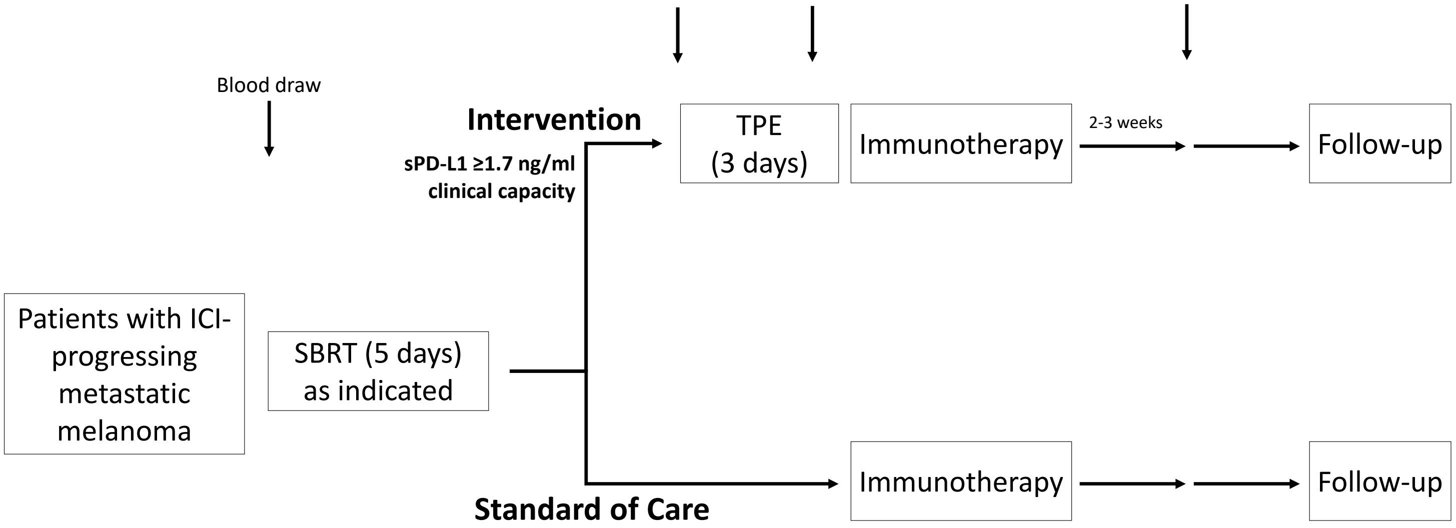
Blood draw
Blood draw
Blood draw
Blood draw
Intervention
sPD-L1 ≥1.7 ng/ml
clinical capacity
TPE
(3 days)
Immunotherapy
2-3 weeks
Follow-up
Patients with ICI-progressing metastatic melanoma
SBRT (5 days) as indicated
Immunotherapy
Follow-up
Standard of Care

1 SPIRIT 2013 REGISTRATION DATA SET

Data category	Information
Primary registry and trial identifying number	ClinicalTrials.gov NCT04581382
Date of registration in primary registry	October 9, 2020
Secondary identifying numbers	MC200703 v2
Protocol version	Version 2 2/2021
Source(s) of monetary or material support	NCI CA259236 (R21)<br>Lawrence W. and Marilyn W. Matteson Award
Primary sponsor	Mayo Clinic
Secondary sponsor(s)	
Contact for public queries	JJO, orme.jacob@mayo.edu
Contact for scientific queries	JJO, orme.jacob@mayo.edu
Public title	Rescuing Cancer Immunity by Plasma Exchange in Metastatic Melanoma (ReCIPE-M1): An open-label safety trial
Scientific title	*Rescuing Cancer Immunity by Plasma Exchange in Metastatic Melanoma (ReCIPE-M1): An open-label safety trial*
Roles and responsibilities	Study Chair: Jacob Orme, M.D., Ph.D<br>Study Co-Chairs: Sean Park, M.D., Ph.D., MS, Jeffrey Winters, M.D., Haidong Dong, M.D., Ph.D., Fabrice Lucien, Ph.D.; Svetomir Markovic, M.D., Ph.D., Matthew Block, M.D., Ph.D., Robert McWilliams, M.D., Lisa Kottschade, APRN, C.N.P.<br>Statistician: Nathan Foster, MS

Data category	Information
	Protocol manuscript author: Tara Davidson, MD<br><br>TD wrote the manuscript. JJO developed hypotheses and concepts, performed initial studies, generated figures, and helped write the manuscript. SSP and JLW provided mentorship, equipment, facilities, edits, and funding. FL and SM provided additional logistical input, planning, and edits.<br><br>Approved by the Mayo Clinic Institutional Review Board (IRB). Mayo Clinic independent data monitoring committee (IDMC) manages data in secured database
Countries of recruitment	USA
Health condition(s) or problem(s) studied	Metastatic melanoma
Intervention(s)<br><br>Pg 8 Line 14-17	Intervention Arm: SBRT (stereotactic body radiation therapy), TPE (therapeutic plasma exchange), ICI (immunotherapy)
	Standard of Care Arm: SBRT (stereotactic body radiation therapy), ICI (immunotherapy)
Key inclusion and exclusion criteria<br><br>Pg 8 Line 4-13	Ages eligible for study: ≥18 years<br>Sexes eligible for study: both<br>Accepts healthy volunteers: no
	Inclusion criteria: adult patient (≥ 18 years), metastatic melanoma, measurable disease per RECIST criteria, ECOG 3 or better
	Exclusion criteria: pregnancy or nursing, consumption of biotin supplements
Study type<br><br>Pg 7 Line 20	Interventional
	Allocation: selection by presentation criteria
	Primary purpose: treatment
	Phase I

Data category	Information
Date of first enrolment	December 2020
Target sample size<br>Pg 8 Line 7-8	60
Recruitment status<br>Pg 8 Line 7-8	Recruiting<br>Listing the trial as a treatment option for eligible patients. 60 patients will be screened from December 2020 through October 2023 with an accrual goal of 20 patients to the TPE Intervention Arm. The principal investigator and study statistician will review the study at least monthly to identify accrual problems.
Primary outcome(s)<br>Pg 7 Line 20-21	Safety (adverse events at 1 and 6 months)
Key secondary outcomes<br>Pg 7 Line 20-22 and Pg 8 Line 1-2	Kinetics of evPD-L1, sPD-L1, and related immunosuppressive metabolites immediately and 2-3 weeks after TPE<br>Relative response of patients to combined therapy through response rate<br>Overall survival<br>Progression-free survival.
Data collection methods and management	Data will be maintained on a secure database with de-identified patient information. Data will be shared on publication in a peer-reviewed journal without the aid of professional writers.
Statistical methods<br>Pg 11 Line 12-14	Wilcoxon Signed-Rank test will be used to assess changes in sPD-L1 and evPD-L1 levels over time across the different timepoints of interest.

Data category	Information
Ethics and Dissemination<br>Pg 12 Line 10-22, Pg 13 Line 1-9	Any modifications to the protocol which may impact on the conduct of the study, potential benefit of the patient or may affect patient safety, including changes of study objectives, study design, patient population, sample sizes, study procedures, or significant administrative aspects will require a formal amendment to the protocol. Such amendment will be approved by the Mayo Clinic IRB prior to implementation and notified to the health authorities in accordance with local regulations.<br><br>Administrative changes of the protocol are minor corrections and/or clarifications that have no effect on the way the study is to be conducted. These administrative changes will be documented in a memorandum. The Ethics Committee/IRB may be notified of administrative changes at the discretion of the study chair and co-chairs.
Ancillary and post-trial care	Patients that are enrolled into the study are covered by indemnity for negligent harm through the standard NHS [*National Health Service*] Indemnity arrangements.

For peer review only - http://bmjopen.bmj.com/site/about/guidelines.xhtml

Data category	Information
Amendments to the protocol	Protocol amended 3/2021 to allow central access for patients without patent peripheral blood access.
Model Informed Consent	See attached

For peer review only - http://bmjopen.bmj.com/site/about/guidelines.xhtml

Mayo Clinic: Department of Radiation Oncology

MC200703: Radiation Therapy, Plasma Exchange, and Immunotherapy in Melanoma

Study Chairs: Jacob Orme, M.D., Ph.D.
Mayo Clinic
Department of Radiation Oncology
200 First Street SW
Rochester MN 55905

Study Co-chairs: Sean Park, M.D., Ph.D., MS
Jeffrey Winters, M.D.
Haidong Dong, M.D., Ph.D.
Fabrice Lucien, Ph.D.
Svetomir Markovic, M.D., Ph.D.
Matthew Block, M.D., Ph.D.
Robert McWilliams, M.D.
Lisa Kottschade, APRN, C.N.P.

Statistician: Nathan Foster, MS √

Study Contributor(s): Dawn M. Deno, BS, CCRP √

√ Study contributor(s) not responsible for patient care

Document History

Version 1	4/2/2020
Version 2	10/14/2020
Version 3	2/8/2021

For peer review only

Protocol Resources

Questions:	Contact Name:
Patient eligibility*, test schedule, treatment delays/interruptions/adjustments, dose modifications, adverse events, forms completion and submission, adverse events, forms completion and submission	Study Coordinator – Sarah Aug<br>Phone: 507-266-2348<br>E-mail: aug.sarah@mayo.edu
Protocol document, consent form	Dawn M. Deno, BS, CCRP<br>Phone: 507-293-7964<br>E-mail: deno.dawn@mayo.edu
Regulatory issues	Justine Buroker, MBA, CCRP<br>Phone: 507-293-0624<br>E-mail: buroker.justine@mayo.edu
Statistician	Nathan Foster, MS<br>Phone: 507-284-5051<br>E-mail: foster.nathan@mayo.edu

For peer review only

Table of Contents

Protocol Resources 2
Table of Contents 3
Study Schema 4
List of Abbreviations 5
1. Background 6
2. Goals 8
3. Patient Eligibility 9
4. Test Schedule 11
5. Grouping Factors: 13
6. Registration Procedures 14
7. Protocol Treatment 15
8. Dosage Modification Based on Adverse Events 17
9. Ancillary Treatment/Supportive Care 18
10. Adverse Event (AE) Monitoring and Reporting 19
11. Treatment Evaluation/Measurement of Effect 25
12. Descriptive Factors 33
13. Treatment/Follow–up Decision at Evaluation of Patient 34
14. Body Fluid Biospecimens 35
15. Drug Information 37
16. Statistical Considerations and Methodology 39
17. Pathology Considerations/Tissue Biospecimens 43
18. Records and Data Collection Procedures 44
19. Budget 46
20. References 47
Appendix I ECOG Performance Status 49
Appendix II - Immune-Related Adverse Events Questionnaire 50

For peer review only

Study Schema

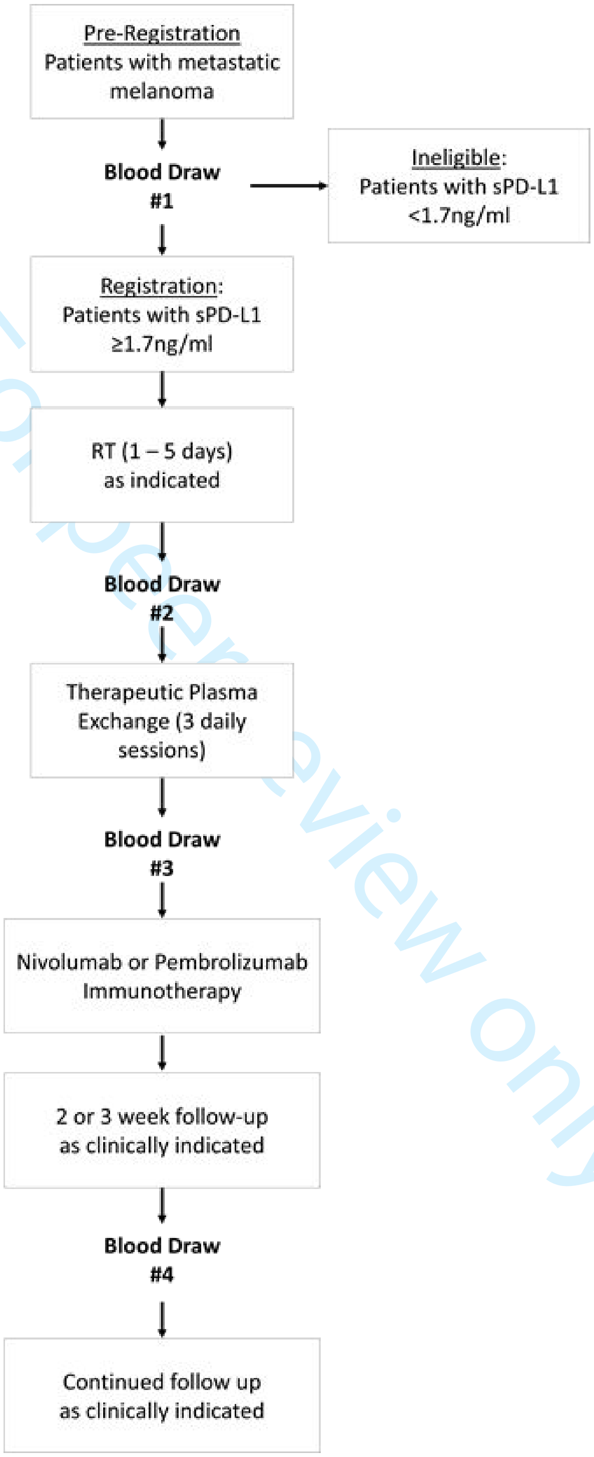

For peer review only

List of Abbreviations

AE	Adverse Event/Adverse Experience
CFR	Code of Federal Regulations
CRF	Case Report Form
DSMB	Data and Safety Monitoring Board
FDA	Food and Drug Administration
GCP	Good Clinical Practice
HIPAA	Health Insurance Portability and Accountability Act
PBT	Proton beam therapy
PI	Principal Investigator
PHI	Protected Health Information
SAE	Serious Adverse Event/Serious Adverse Experience
IRB	Institutional Review Board
ReRT	Re-irradiation
OS	Overall survival
PFS	Progression-free survival
LRC	Locoregional control
RTOG	Radiation Therapy Oncology Group
ECOG	Eastern Cooperative Oncology Group
SFO	Single Field Optimization
MFO	Multi Field Optimization
IFSO	Individual Field Simultaneous Optimization
IMPT	Intensity Modulated Proton Therapy
SPB	Scanning Proton Beam
GTV	Gross Tumor Volume
CTV	Clinical Tumor Volume
OTV	Optimization Target Volume
STV	Scanning Target Volume
PRV	Planning Risk Volume
OAR	Organs at Risk
RU	Range Uncertainty
PET	Positron Emission Tomography
QoL	Quality of Life
EDC	Electronic Data Collection
LET	Linear Energy Transfer
SOC	System Organ Class
RECIST	Response Evaluation Criteria in Solid Tumors
ULN	Upper limit of normal
ORR	Objective Response Rate

For peer review only

1. Background

1.1 Immunotherapy resistance

Almost 3 million patients will receive anti-PD-1 or anti-PD-L1 (henceforth "PD-(L)1 inhibitor") immune checkpoint inhibitors (ICI) to treat their cancer [1], and yet merely 1 in 8 will respond to the treatment [2]. Furthermore, despite initial response to ICI, a majority of the patients succumb to their cancer due to the development of ICI resistance [3]. Recently, we and others have discovered extracellular forms of PD-L1 that mediate PD-(L)1 inhibitor resistance (Fig 1). First, tumors transcribe and secrete soluble PD-L1 (sPD-L1) splice variants [4], [5], and enzymes ADAM10 and ADAM17 also shed sPD-L1 ectodomain directly from the tumor cell surface [6], [7]. Second, tumors generate extracellular vesicles (EVs) bearing surface PD-L1 (evPD-L1) [8], [9]. These extracellular forms of PD-L1 outcompete PD-(L)1 inhibitors, engage and kill CD8+ T cells, and suppress anti-tumor immunity (Fig 1). These forms of PD-L1 at high levels in patient plasma predict poor outcomes in many malignancies [4], [10]–[15].

Thus, *there is a critical need to develop methods to inhibit or remove extracellular PD-L1*. In the absence of such discoveries, most patients who do not respond to PD-(L)1 inhibitors will continue to experience poor survival.

Fig 1 – Extracellular forms of PD-L1 (sPD-L1 and evPD-L1) cause resistance to PD-(L)1 inhibitor therapy in cancer by outcompeting PD-(L)1 inhibitors, killing CD8+ T cells, and limiting anti-tumor immunity.

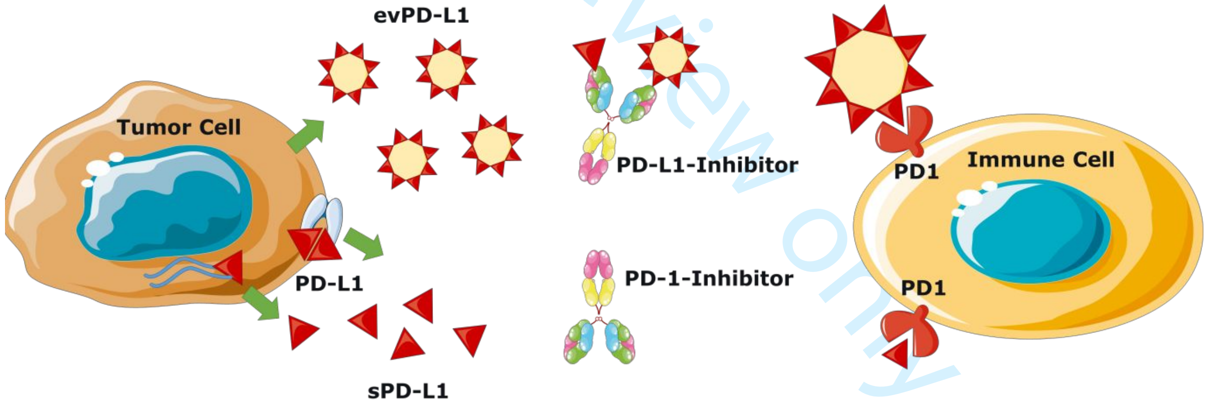

1.2 Therapeutic plasma exchange clears evPD-L1 and sPD-L1

Therapeutic plasma exchange (TPE) is a procedure in which blood is removed from the body and processed to eliminate non-diffusing substances confined to the plasma (Fig 2A). TPE is commonly used clinically to remove pathogenic antibodies in patients with severe autoimmune disease. In our pilot study of 24 patients, TPE significantly depleted sPD-L1 (Fig 2B) and evPD-L1 (Fig 4C) by 70.8% and 73.1%, respectively [16]. This depletion is compounded over three consecutive TPE sessions.

For peer review only - http://bmjopen.bmj.com/site/about/guidelines.xhtml

Fig 2 – Therapeutic Plasma Exchange (TPE) removes sPD-L1 and evPD-L1 from circulation. (A) In TPE, blood is removed and centrifuged to remove substances, such as antibodies, from systemic circulation. (B,C) TPE removed an average of 70.8% of sPD-L1 and 73.1% of evPD-L1 from the blood in one session.

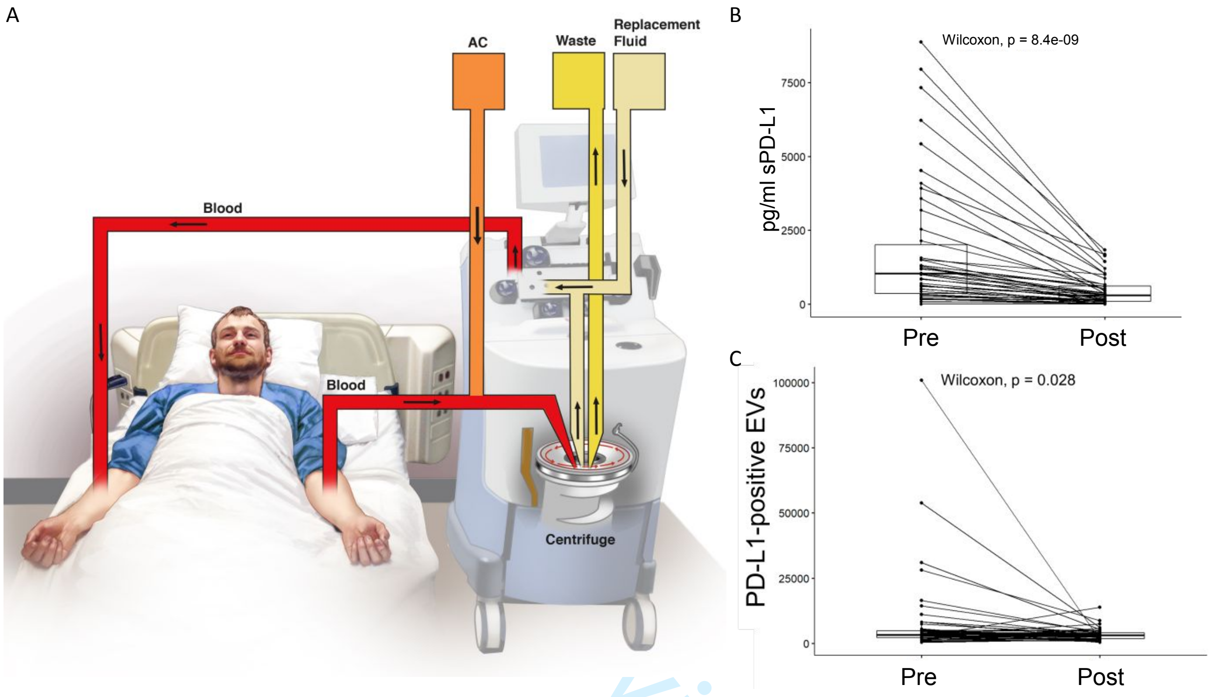

1.3 Defining the kinetics of sPD-L1 and evPD-L1 in melanoma

The *rationale* for this study is that its successful completion will define the kinetic quality of sPD-L1 and evPD-L1 depletion and regeneration in melanoma, without which a full Phase I clinical trial of TPE PD-(L)1 inhibitor rescue cannot be performed. Patients with advanced melanoma who progress on first-line ICI will be enrolled in this study (n=36). Patients with high sPD-L1 levels (> 1.7 ng/ml), ECOG 0-1, life expectancy > 6 months are eligible. After standard-of-care radiation therapy, eligible patients will undergo 3 days of TPE treatment, after which baseline sPD-L1 and evPD-L1 will be measured before standard-of-care immunotherapy is administered. Just prior to the next indicated immunotherapy 2-3 weeks later, sPD-L1 and evPD-L1 will be measured again and compared for regeneration.

2. Goals

2.1. Primary Goal

2.1.1. To determine the kinetics of sPD-L1 removal and regeneration by plasma exchange in patients with melanoma.

2.2. Secondary Goals

2.2.1. To observe response at 3 months after plasma exchange plus immunotherapy.

2.2.2. To observe ongoing response at approximate 3 month intervals after plasma exchange plus immunotherapy.

2.3. Correlative Research

2.3.1. To determine the effects of plasma exchange on immune cell function.

2.3.2. To observe the kinetics of extracellular vesicles (EVs) after plasma exchange in patients with melanoma.

For peer review only

3. Patient Eligibility

3.1. Inclusion Criteria

3.1.1. Age ≥18 years.

3.1.2. Histological confirmation of melanoma. Patients may have completed biopsy outside of Mayo Clinic, but there must be an internal review done to confirm diagnosis prior to confirming eligibility.

3.1.3. Measurable or non-measurable disease as defined in Section 11.0 ~~or~~ .Patient must meet one of these criteria.

3.1.4. ECOG Performance Status (PS) ≤ 3 (Appendix I).

3.1.5. sPD-L1 levels >1.7ng/ml by ELISA [17]

3.1.6. Feasible vascular access as determined by study apheresis technician (either by peripheral intravenous access *or* by placement of a temporary central line)

3.1.7. Negative pregnancy test done ≤7 days prior to radiation therapy, for women of childbearing potential only.

3.1.8. Provide written informed consent.

3.1.9. Willing to return to enrolling institution for follow-up (during the Active Monitoring Phase of the study).

3.1.10. Willing to provide blood samples for correlative research purposes (see Section 6).

3.2. Exclusion Criteria

3.2.1. Any of the following:

3.2.1.1. Persons taking a biotin supplement

3.2.1.2. sPD-L1 level <1.7ng/ml by ELISA [17]

For peer review only

3.2.1.3. Pregnant or nursing women

3.2.1.4. Men or women of childbearing potential who are unwilling to employ adequate contraception

3.2.1.5 ~~Patients that are to receive an invasive procedure(s) within 48 hours prior to receiving TPE; this includes placement of central line.~~

For peer review only

4. Test Schedule

Tests and procedures	Screening	Radiation Therapy	Therapeutic Plasma Exchange[R, 4]		Observation/Follow up until 2 years post registration	
	≤_7_ days prior to registration	*Following Last Fraction of RT*	*Daily during plasma exchange (TPE)*	*Following last plasma exchange (TPE)*	- *Pre-2nd IO[10]*	*At every clinically indicated follow up[2]*
Toxicity Assessments; CTCAE v5.0[S]	X[6]		X[5]	X[5]	X[6]	X[6]
Pregnancy test[1,S]	X					
Vascular Assessment	X[9]					
CBC for Apheresis machine set up[S]	X[8]					
Research blood specimens (see Section 14.0)[R]	X[8]	X[7, 3]		X[7, 3]	X	
Routine Imaging[S]						X
Temporary central line placement (in patients without adequate venous access)[R]	X[11]					
Temporary central line removal (in patients with line)[R]				X		

Radiation Treatment = 1 to 5 fractions, 20 to 60 Gy total, clinician's choice
IO = Immunotherapy, clinician's choice pembrolizumab or nivolumab

1. For women of childbearing potential only. Must be done ≤*7 days* prior to beginning Radiation Treatment as a standard of care procedure.

2. Follow up assessments will be completed as per clinician's discretion, which generally takes place every 3-6 months. Local oncologist's standard of care assessments are acceptable.

3. Blood to becollected in Therapeutic Apheresis Treatment Unit – Charlton 8A.

4. The plasma exchange treatments to begin following completion of RT (same day) or the

For peer review only

following day and will be done in 3 consecutive days.

5. Standard of care AE assessments during exchange will be completed daily by Study Apheresis RN, while doing plasma exchange treatment

6. To be assessed by Medical Oncology using irAE toxicity assessment

7. Following the last fraction of RT (Pre-Initial TPE) and Post-Final TPE 8. Must have results available within 2 weeks of starting TPE for machine set up.

9. Vein access for TPE must be determined by study apheresis technician

10. 2 weeks post first IO if receiving **nivolumab**, 3 weeks post first IO if receiving **pembrolizumab**

11. Eligible, enrolled patients determined to require central line placement will undergo Mahurkar line placement by interventional radiology 48-72 hours prior to first TPE procedure. Central line will be removed at apharesis unit at completion of final plasma exchange.

R Research funded (see Section 19.0)

S Standard of care

4.1. Observation/Follow-up

	**Observation/Follow-up[1]**			
	As indicated until PD	At PD	Death	New Primary
Event Monitoring	X	X	X	At each occurrence

1. If a patient is still alive 2 years after registration, no further follow-up is required. Follow up will include: Survival status, progression status, and toxicity assessment.

For peer review only

5. Grouping Factors:

5.1. High versus low baseline evPD-L1 (cutoffs >1 million or 0.05%)

For peer review only

6. Registration Procedures

6.1. Registration

Patient will be registered to the study when they have consented, met eligibility criteria and have been logged into the Research Participant Tracking System (Ptrax).

6.1.1. Verify the following procedures are completed prior to Registration to the study.

6.1.2. IRB approval at the registering institution

6.1.3. Existence of a signed informed consent

6.1.4. Patient eligibility complete

Note: Radiation Therapy on this protocol must be performed at Mayo Clinic Rochester under the supervision of a radiation oncologist.

For peer review only

7. Protocol Treatment

7.1. Radiation Therapy (RT) if indicated

Given per treating radiation oncologist discretion on days D1 through D5. The prescription dose is expected to be up to 60 Gy delivered in up to 5 fractions but not stipulated by the study. Treatment will be delivered daily except on weekends and holidays at the discretion of the treating radiation oncologist.

7.2. Line placement if indicated

In patients without adequate peripheral iv access as determined by apheresis unit nursing staff, a temporary Mahurkar central line will be placed by interventional radiology to enable therapeutic plasma exchange. This will be performed on D2 or D3, at least 48 hours prior to first therapeutic plasma exchange session. Line will be removed by trained apheresis unit staff after final therapeutic plasma exchange session.

~~7.2.~~7.3. Therapeutic plasma exchange (TPE)

Three sessions performed on consecutive days, D5 through D7 or D4 through D6 before immunotherapy on D7. This procedure is *not* performed prior to subsequent immunotherapy in future cycles.

~~7.3.~~7.4. Concurrent immunotherapy (IO)

According to clinician preference, either pembrolizumab or nivolumab will be given on D7 after therapeutic plasma exchange. This immunotherapy will be continued per clinician judgment according at 3-week (for pembrolizumab) or 2-week (for nivolumab) intervals as clinically indicated until progression and/or adverse events.

Agent	Dose	Days administered	Pre-medication*
Pembrolizumab (Keytruda®)	200mg IV	D7	None
Nivolumab (Opdivo®)	240mg IV	D7	None

~~7.4.~~7.5. Follow up protocol

~~7.4.1.~~7.5.1. Follow-up imaging

For peer review only

Standard of care CT, PET, and/or MRI scans will be used as clinically indicated and ordered for follow up imaging.

7.5.7.6. Response assessment

7.5.1.7.6.1. Post-treatment tumor recurrence will be monitored with follow-up imaging to assess tumor response based on RECIST criteria until progression or death (or up to 2 years)

7.6.7.7. Outcomes

Patient-centric outcomes measured will include time to progression and time to death if applicable. These will be determined by chart review.

7.7.7.8. Acute and late toxicity monitoring

Toxicity will be measured by standard-of-care irAE forms. No known side-effect of plasma exchange constitutes an adverse event that is not anticipated by irAE monitoring.

7.8.7.9. Dose Modification Based on Adverse Event

Dose modification for immunotherapies in future cycles will be at the discretion of the treating physician.

7.9.7.10. Return to consenting institution if applicable,

For this protocol, the patient must return to the consenting institution for evaluation at least every 182 days during treatment. Local treating physician evaluations, if done according to standard of care, are also acceptable after blood draws have been completed.

For peer review only

8. Dosage Modification Based on Adverse Events

8.1. Initial treatment with TPE carries low but possible risk of hypotension, infection, or excessive bleeding. Should any patient experience an adverse event related to plasma exchange, TPE will be discontinued.

8.2. Dose modification of pembrolizumab or nivolumab will be performed according to clinician preference over the course of the study. These changes are made at the discretion of the treating physician.

For peer review only

9. Ancillary Treatment/Supportive Care

9.1. Antiemetics

Antiemetics may be used at the discretion of the attending physician.

9.2. Premedication for chemotherapy will be given per institutional protocols.

9.3. No biotin-containing compounds will be administered or prescribed.

For peer review only

10. Adverse Event (AE) Monitoring and Reporting

10.1. Definitions

10.1.1. Adverse Event: Any untoward medical occurrence associated with the use of a drug in humans, whether or not considered drug related.

10.1.2. Serious Adverse Event: Adverse events are classified as serious or non-serious. Serious problems/events can be well defined and include:

10.1.2.1. Death

10.1.2.2. Life threatening adverse experience

10.1.2.3. Hospitalization

10.1.2.4. Inpatient, new, or prolonged; disability/incapacity

10.1.2.5. Persistent or significant birth defect/anomaly

10.1.2.6. and/or per protocol may be problems/events that in the option of the sponsor investigator may have adversely affected the rights, safety, or welfare of the subjects or others, or substantially comprised the research data. All adverse events that do not meet any of the criteria for serious, should be regarded as non-serious adverse events.

Unanticipated Problems Involving Risks to Subjects or Others (UPIRTSO)- Any unanticipated problem or adverse event that meets the following three criteria:

- Serious: Serious problems or events that results in significant harm, (which may be physical, psychological, financial, social, economic, or legal) or increased risk for the subject or others (including individuals who are not research subjects). These include: (1) death; (2) life threatening adverse experience; (3) hospitalization - inpatient, new, or prolonged; (4) disability/incapacity - persistent or significant; (5) birth defect/anomaly; (6) breach of confidentiality and (7) other problems, events, or new information (i.e. publications, DSMB reports, interim findings, product labeling change) that in the opinion of the local investigator may adversely affect the rights, safety, or welfare of the subjects or others, or substantially compromise the research data,

  AND

- Unanticipated: (i.e. unexpected) problems or events are those that are not already described as potential risks in the protocol, consent document, not listed in the Investigator's Brochure, or not part of an underlying disease. A problem or event is "unanticipated" when it was unforeseeable at the time of its occurrence. A problem or event is

For peer review only

"unanticipated" when it occurs at an increased frequency or at an increased severity than expected, AND

- Related: A problem or event is "related" if it is possibly related to the research procedures.
- Preexisting Condition- A preexisting condition is one that is present at the start of the study. A preexisting condition should be recorded as an adverse event if the frequency, intensity, or the character of the condition worsens during the study period. At screening, any clinically significant abnormality should be recorded as a preexisting condition. At the end of the study, any new clinically significant findings/abnormalities that meet the definition of an adverse event must also be recorded and documented as an adverse event.

10.1.3. Suspected Adverse Reaction: Any adverse event for which there is a reasonable possibility that the drug or procedure caused the adverse event.

10.2. Recording Adverse Events/Treatment being studied

CTCAE term (AE description) and grade: The descriptions and grading scales found in the revised NCI Common Terminology Criteria for Adverse Events (CTCAE) version 5.0 will be utilized for AE reporting. All appropriate treatment areas should have access to a copy of the CTCAE version 5.0. A copy of the CTCAE version 5.0 can be downloaded from the CTEP web site: (http://ctep.cancer.gov/protocolDevelopment/electronic_applications/ctc.htm)

10.2.1. Adverse event monitoring and reporting is a routine part of every clinical trial. First, identify and grade the severity of the event using the CTCAE version 5.0. Next, determine whether the event is expected or unexpected and if the adverse event is related to the medical treatment or procedure. With this information, determine whether the event must be reported as an expedited report (see Section 10.25).

Expedited and routine reports are to be completed within the timeframes and via the mechanisms specified in Sections 10.4 and 10.6. All expected AE reports must also be sent to the local Institutional Review Board (IRB) according to local IRB's policies and procedures.

10.2.1.1.1.1. When assessing whether an adverse event (AE) is related to a medical agent(s) medical or procedure, the following attribution categories are utilized:

For peer review only

Definite - The AE is clearly related to the agent(s)/procedure.

Probable - The AE is likely related to the agent(s)/procedure.

Possible - The AE may be related to the agent(s)/procedure.

Unlikely - The AE is doubtfully related to the agent(s)/procedure.

Unrelated - The AE is clearly NOT related to the agent(s)/procedure.

10.2.2. Exceptions to Expedited Reporting For Immunotherapy Related Toxicities

An expedited report may not be required for specific Grade 1, 2 and 3 serious Adverse Events. Any protocol specific reporting procedures MUST BE SPECIFIED BELOW and will supersede the standard Expedited Adverse Event Reporting Requirements**: Hospitalizations for reasons deemed to be disease related will not be reported.**

**CTCAE System Organ Class (SOC)**	**Adverse event/ Symptoms**	**CTCAE Grade at which the event will not be reported in an expedited manner[1]**
General disorders and administrations site conditions	Fatigue	*≤Grade 3*
	Malaise	*≤Grade 3*
Skin and subcutaneous/ Tissue Disorders	Alopecia	*≤Grade 4*

[1] These exceptions only apply if the adverse event does not result in hospitalization. If the adverse event results in hospitalization, then the standard expedited adverse events reporting requirements must be followed.
Specific protocol exceptions to expedited reporting should be reported expeditiously by investigators **ONLY** if they exceed the expected grade of the event.

[1] These exceptions only apply if the adverse event does not result in hospitalization. If the adverse event results in hospitalization, then the standard expedited adverse events reporting requirements must be followed.
Specific protocol exceptions to expedited reporting should be reported expeditiously by investigators **ONLY** if they exceed the expected grade of the event.

For peer review only

10.3. Other Required Reporting

10.3.1. Unanticipated Problems Involving Risks to Subjects or Others (UPIRTSOS)

Unanticipated Problems Involving Risks to Subjects or Others (UPIRTSOS) in general, include any incident, experience, or outcome that meets **all** of the following criteria:

1. Unexpected (in terms of nature, severity, or frequency) given (a) the research procedures that are described in the protocol-related documents, such as the IRB-approved research protocol and informed consent document; and (b) the characteristics of the subject population being studied;
2. Related or possibly related to participation in the research (in this guidance document, possibly related means there is a reasonable possibility that the incident, experience, or outcome may have been caused by the procedures involved in the research); and
3. Suggests that the research places subjects or others at a greater risk of harm (including physical, psychological, economic, or social harm) than was previously known or recognized.

Some unanticipated problems involve social or economic harm instead of the physical or psychological harm associated with adverse events. In other cases, unanticipated problems place subjects or others at increased *risk* of harm, but no harm occurs.

Note: If there is no language in the protocol indicating that pregnancy is not considered an adverse experience for this trial, and if the consent form does not indicate that subjects should not get pregnant/impregnate others, then any pregnancy in a subject/patient or a male patient's partner (spontaneously reported) which occurs during the study or within

10.3.2. Death

Note: A death on study requires both routine and expedited reporting regardless of causality, unless as noted below. Attribution to treatment or other cause must be provided.

Any death occurring within 30 days of the last dose, regardless of attribution to an agent/intervention under an IND/IDE requires expedited reporting within 24-hours.

For peer review only

Any death occurring greater than 30 days with an attribution of possible, probable, or definite to an agent/intervention under an IND/IDE requires expedited reporting within 24-hours.

Reportable categories of Death

- Death attributable to a CTCAE term.
- Death Neonatal: A disorder characterized by cessation of life during the first 28 days of life.
- Death NOS: A cessation of life that cannot be attributed to a CTCAE term associated with Grade 5.
- Sudden death NOS: A sudden (defined as instant or within one hour of the onset of symptoms) or an unobserved cessation of life that cannot be attributed to a CTCAE term associated with Grade 5.
- Death due to progressive disease should be reported as **Grade 5 "Neoplasms benign, malignant and unspecified (including cysts and polyps) – Other (Progressive Disease)"** under the system organ class (SOC) of the same name. Evidence that the death was a manifestation of underlying disease (e.g., radiological changes suggesting tumor growth or progression: clinical deterioration associated with a disease process) should be submitted.

10.4. Required Routine Reporting

10.4.1. Toxicity Evaluations

Pretreatment symptoms/conditions to be graded at baseline and adverse events to be graded at each evaluation.

Grading is per CTCAE v5.0 **unless** alternate grading is indicated in the table below: (this table outlines the AE's you want to have assessed at each study visit the patient has)

CTCAE System/Organ/Class (SOC)	Adverse event/Symptoms	Baseline	Each evaluation
Gastrointestinal	*Diarrhea*	x	x
Dermatologic	*Rashes*	x	x

For peer review only

CTCAE System/Organ/Class (SOC)	Adverse event/Symptoms	Baseline	Each evaluation
Respiratory	*Pneumonitis*	x	x
Adrenal	*Hypophysitis*	x	x
Rheumatologic	*Myalgias, arthralgias*	x	x
Liver	*Hepatitis*	x	x

For peer review only

10.5. Monitoring and Auditing

The investigator will permit study-related monitoring, audits, and inspections by the IRB, the sponsor, and government regulatory agencies, of all study related documents (e.g. source documents, regulatory documents, data collection instruments, study data etc.). The investigator will ensure the capability for inspections of applicable study-related facilities (e.g. pharmacy, diagnostic laboratory, etc.). Participation as an investigator in this study implies acceptance of potential inspection by government regulatory authorities and applicable compliance offices

10.5.1. Medical Monitoring

It is the responsibility of the Principal Investigator to oversee the safety of the study at his/her site. This safety monitoring will include careful assessment and appropriate reporting of adverse events as noted above, as well as the construction and implementation of a site data and safety-monitoring plan (see section 10.5 "Monitoring and Auditing"). Medical monitoring will include a regular assessment of the number and type of serious adverse events. "Any serious adverse events will be followed up by the sentinel event reporting procedure"

10.5.2. Internal Data and Safety Monitoring Board

The trial will be reviewed by the Cancer Center Auditing area on a bi-annual or yearly basis dependent on random study selection to assess accrual, adverse events, and any endpoint problems. Any safety issues requiring protocol changes will be communicated through protocol amendments.

11. Treatment Evaluation/Measurement of Effect

Response and progression will be evaluated in this study using the new international criteria proposed by the revised Response Evaluation Criteria in Solid Tumors (RECIST) guidelines (version 1.1) [18]. Changes in the largest diameter (unidimensional measurement) of the tumor lesions and the short axis measurements in the case of lymph nodes are used in the RECIST guideline.

11.1. Schedule of Evaluations:

11.1.1. For the purposes of this study, patients should be reevaluated every 3-6 months according to clinical judgment.

11.2. Definitions of Measurable and Non-Measurable Disease

11.2.1. Measurable Disease

11.2.1.1. A non-nodal lesion is considered measurable if its longest diameter can be accurately measured as ≥2.0 cm with chest x-ray, or as ≥1.0 cm with CT scan, CT component of a PET/CT, or MRI.

11.2.1.2. A superficial non-nodal lesion is measurable if its longest diameter is ≥ 1.0 cm in diameter as assessed using calipers (e.g. skin nodules) or imaging. In the case of skin lesions, documentation by color photography, including a ruler to estimate the size of the lesion, is recommended.

11.2.1.3. A malignant lymph node is considered measurable if its short axis is >1.5 cm when assessed by CT scan (CT scan slice thickness recommended to be no greater than 5 mm).

NOTE: Tumor lesions in a previously irradiated area are not considered measurable disease.

11.2.2. Non-Measurable Disease

11.2.2.1. All other lesions (or sites of disease) are considered non-measurable disease, including pathological nodes (those with a short axis ≥1.0 to <1.5 cm). Bone lesions, leptomeningeal disease, ascites, pleural/pericardial effusions, lymphangitis cutis/pulmonis, inflammatory breast disease, and abdominal masses (not followed by CT or MRI), are considered as non-measurable as well.

For peer review only - http://bmjopen.bmj.com/site/about/guidelines.xhtml

Note: 'Cystic lesions' thought to represent cystic metastases can be considered as measurable lesions, if they meet the definition of measurability described above. However, if non-cystic lesions are present in the same patient, these are preferred for selection as target lesions. In addition, lymph nodes that have a short axis <1.0 cm are considered non-pathological (i.e., normal) and should not be recorded or followed.

11.3. Guidelines for Evaluation of Measurable Disease

11.3.1. Measurement Methods:

- All measurements should be recorded in metric notation (i.e., decimal fractions of centimeters) using a ruler or calipers.
- The same method of assessment and the same technique must be used to characterize each identified and reported lesion at baseline and during follow-up. For patients having only lesions measuring at least 1 cm to less than 2 cm must use CT imaging for both pre- and post-treatment tumor assessments.
- Imaging-based evaluation is preferred to evaluation by clinical examination when both methods have been used at the same evaluation to assess the antitumor effect of a treatment.

11.3.2. Acceptable Modalities for Measurable Disease:

- Conventional CT and MRI: This guideline has defined measurability of lesions on CT scan based on the assumption that CT slice thickness is 5 mm or less. If CT scans have slice thickness greater than 5 mm, the minimum size for a measurable lesion should be twice the slice thickness.

- As with CT, if an MRI is performed, the technical specifications of the scanning sequences used should be optimized for the evaluation of the type and site of disease. The lesions should be measured on the same pulse sequence. Ideally, the same type of scanner should be used and the image acquisition protocol should be followed as closely as possible to prior scans. Body scans should be performed with breath-hold scanning techniques, if possible.

- PET-CT: If the site can document that the CT performed as part of a PET-CT is of identical diagnostic quality to a diagnostic CT (with IV and oral contrast), then the CT portion of the PET-CT can be used for RECIST measurements and can be used interchangeably with conventional CT in accurately measuring cancer lesions over time.

For peer review only

- Chest X-ray: Lesions on chest x-ray are acceptable as measurable lesions when they are clearly defined and surrounded by aerated lung. However, CT scans are preferable.

- Physical Examination: For superficial non-nodal lesions, physical examination is acceptable, but imaging is preferable, if both can be done. In the case of skin lesions, documentation by color photography, including a ruler to estimate the size of the lesion, is recommended.

- FDG-PET: FDG-PET scanning is allowed to complement CT scanning in assessment of progressive disease [PD] and particularly possible 'new' disease. A 'positive' FDG-PET scanned lesion is defined as one which is FDG avid with an update greater than twice that of the surrounding tissue on the attenuation corrected image; otherwise, an FDG-PET scanned lesion is considered 'negative.' New lesions on the basis of FDG-PET imaging can be identified according to the following algorithm:

  a. Negative FDG-PET at baseline with a positive FDG-PET at follow-up is a sign of PD based on a new lesion.

  b. No FDG-PET at baseline and a positive FDG-PET at follow-up:

     i. If the positive FDG-PET at follow-up corresponds to a new site of disease confirmed by CT, this is PD.

     ii. If the positive FDG-PET at follow-up is not confirmed as a new site of disease on CT at the same evaluation, additional follow-up CT scans (i.e., additional follow-up scans at least 4 weeks later) are needed to determine if there is truly progression occurring at that site. In this situation, the date of PD will be the date of the initial abnormal PDG-PET scan.

     iii If the positive FDG-PET at follow-up corresponds to a pre-existing site of disease on CT that is not progressing on the basis of the anatomic images, it is not classified as PD.

11.3.3. Measurement at Follow-up Evaluation:

- In the case of stable disease (SD), follow-up measurements must have met the SD criteria at least once after study entry at a minimum interval of 6 weeks.

- The cytological confirmation of the neoplastic origin of any effusion that appears or worsens during treatment when the measurable tumor has

For peer review only

met criteria for response or stable disease is mandatory to differentiate between response or stable disease (an effusion may be a side effect of the treatment) and progressive disease.

- Cytologic and histologic techniques can be used to differentiate between PR and CR in rare cases (e.g., residual lesions in tumor types such as germ cell tumors, where known residual benign tumors can remain.)

11.4. Measurement of Effect

11.4.1. Target Lesions and Target Lymph Nodes

- Measurable lesions (as defined in Section 11.2.1) up to a maximum of 5 lesions, representative of all involved organs, should be identified as "Target Lesions" and recorded and measured at baseline. These lesions can be non-nodal or nodal (as defined in 11.2.1), where no more than 2 lesions are from the same organ and no more than 2 malignant nodal lesions are selected.

  Note: If fewer than 5 target lesions and target lymph nodes are identified (as there often will be), there is no reason to perform additional studies beyond those specified in the protocol to discover new lesions.

- Target lesions and target lymph nodes should be selected on the basis of their size, be representative of all involved sites of disease, but in addition should be those that lend themselves to reproducible repeated measurements. It may be the case that, on occasion, the largest lesion (or malignant lymph node) does not lend itself to reproducible measurements in which circumstance the next largest lesion (or malignant lymph node) which can be measured reproducibly should be selected.

- Baseline Sum of Dimensions (BSD): A sum of the longest diameter for all target lesions plus the sum of the short axis of all the target lymph nodes will be calculated and reported as the baseline sum of dimensions (BSD). The BSD will be used as reference to further characterize any objective tumor response in the measurable dimension of the disease.

- Post-Baseline Sum of the Dimensions (PBSD): A sum of the longest diameter for all target lesions plus the sum of the short axis of all the target lymph nodes will be calculated and reported as the post-baseline sum of dimensions (PBSD). If the radiologist is able to provide an actual measure for the target lesion (or target lymph node), that should

For peer review only

be recorded, even if it is below 0.5 cm. If the target lesion (or target lymph node) is believed to be present and is faintly seen but too small to measure, a default value of 0.5 cm should be assigned. If it is the opinion of the radiologist that the target lesion or target lymph node has likely disappeared, the measurement should be recorded as 0 cm.

- The minimum sum of the dimensions (MSD) is the minimum of the BSD and the PBSD.

11.4.2. Non-Target Lesions & Non-Target Lymph Nodes

Non-measurable sites of disease (Section 11.2.2) are classified as non-target lesions or non-target lymph nodes and should also be recorded at baseline. These lesions and lymph nodes should be followed in accord with the above.

11.4.3. Response Criteria

11.4.3.1. All target lesions and target lymph nodes followed by CT/MRI/PET-CT/Chest X-ray/physical examination must be measured on re-evaluation at evaluation times specified in Section 11.1. Specifically, a change in objective status to either a PR or CR cannot be done without re-measuring target lesions and target lymph nodes.

Note: Non-target lesions and non-target lymph nodes should be evaluated at each assessment, especially in the case of first response or confirmation of response. In selected circumstances, certain non-target organs may be evaluated less frequently. For example, bone scans may need to be repeated only when complete response is identified in target disease or when progression in bone is suspected.

11.4.3.2. Evaluation of Target Lesions

Complete Response (CR):

All of the following must be true:

a. Disappearance of all target lesions.

b. Each target lymph node must have reduction in short axis to <1.0 cm.

Partial Response (PR):

At least a 30% decrease in PBSD (sum of the longest diameter for all target lesions plus the sum of the

For peer review only - http://bmjopen.bmj.com/site/about/guidelines.xhtml

short axis of all the target lymph nodes at current evaluation) taking as reference the BSD (*see* Section 11.4.1).

Progression (PD):
At least one of the following must be true:

a. At least one new malignant lesion, which also includes any lymph node that was normal at baseline (< 1.0 cm short axis) and increased to ≥ 1.0 cm short axis during follow-up.

b. At least a 20% increase in PBSD (sum of the longest diameter for all target lesions plus the sum of the short axis of all the target lymph nodes at current evaluation) taking as reference the MSD (Section 11.4.1). In addition, the PBSD must also demonstrate an absolute increase of at least 0.5 cm from the MSD.

c. See Section 11.32 for details in regards to the requirements for PD via FDG-PET imaging.

Stable Disease (SD):
Neither sufficient shrinkage to qualify for PR, nor sufficient increase to qualify for PD taking as reference the MSD.

11.4.3.3. Evaluation of Non-Target Lesions & Non-Target Lymph Nodes

Complete Response (CR):
All of the following must be true:

a. Disappearance of all non-target lesions.

b. Each non-target lymph node must have a reduction in short axis to <1.0 cm.

Non-CR/Non-PD: Persistence of one or more non-target lesions or non-target lymph nodes.

Progression (PD): At least one of the following must be true:

For peer review only

a. At least one new malignant lesion, which also includes any lymph node that was normal at baseline (< 1.0 cm short axis) and increased to ≥ 1.0 cm short axis during follow-up.

b. Unequivocal progression of existing non-target lesions and non-target lymph nodes. (NOTE: Unequivocal progression should not normally trump target lesion and target lymph node status. It must be representative of overall disease status change.)

c. See Section 11.3.2 for details in regards to the requirements for PD via FDG-PET imaging.

11.4.4. Overall Objective Status

The overall objective status for an evaluation is determined by combining the patient's status on target lesions, target lymph nodes, non-target lesions, non-target lymph nodes, and new disease as defined in the following table:

For Patients with Measurable Disease

Target Lesions & Target Lymph Nodes	Non-Target Lesions & Non-Target Lymph Nodes	New Sites of Disease	Overall Objective Status
CR	CR	No	CR
CR	Non-CR/Non-PD	No	PR
PR	CR<br>Non-CR/Non-PD	No	PR
CR/PR	Not All Evaluated*	No	PR**
SD	CR<br>Non-CR/Non-PD<br>Not All Evaluated*	No	SD
Not all Evaluated	CR<br>Non-CR/Non-PD<br>Not All Evaluated*	No	Not Evaluated (NE)
PD	Unequivocal PD<br>CR<br>Non-CR/Non-PD<br>Not All Evaluated*	Yes or No	PD

For peer review only

Target Lesions & Target Lymph Nodes	Non-Target Lesions & Non-Target Lymph Nodes	New Sites of Disease	Overall Objective Status
CR/PR/SD/PD/Not all Evaluated	Unequivocal PD	Yes or No	PD
CR/PR/SD/PD/Not all Evaluated	CR<br>Non-CR/Non-PD<br>Not All Evaluated*	Yes	PD

*See Section 11.4.3.1
** NOTE: This study uses the protocol RECIST v1.1 template dated 2/16/2011. For data collection and analysis purposes the objective status changed from SD to PR in the NCCTG protocol RECIST v1.1 template as of 2/16/2011 and to match RECIST v1.1 requirements.

11.4.5. Symptomatic Deterioration

Patients with global deterioration of health status requiring discontinuation of treatment without objective evidence of disease progression at that time, and not either related to study treatment or other medical conditions, should be reported as PD due to "symptomatic deterioration." Every effort should be made to document the objective progression even after discontinuation of treatment due to symptomatic deterioration. A patient is classified as having PD due to "symptomatic deterioration" if any of the following occur that are not either related to study treatment or other medical conditions:

- Weight loss >10% of body weight.
- Worsening of tumor-related symptoms.
- Decline in performance status of >1 level on ECOG scale.

For peer review only

12. Descriptive Factors

12.1. Prior treatment: 1 vs 2 vs 3+

12.2. Disease stage

12.3. Patient age

12.4. Other medical problems

12.5. Baseline sPD-L1

12.6. Baseline evPD-L1

For peer review only

13. Treatment/Follow–up Decision at Evaluation of Patient

13.1. A patient is deemed *ineligible* if after registration, it is determined that at the time of registration, the patient did not satisfy each and every eligibility criteria for study entry. The patient will go off study.

13.1.1. If the patient received CT, MR, and/or PET for radiotherapy planning, all data up until the point of confirmation of ineligibility must be submitted.

13.1.2. If the patient never received plasma exchange, on-study material must be submitted. No additional follow up is necessary.

13.2. Those patients who will not receive any radiation treatment or who will receive radiation treatment elsewhere will go off study.

13.3. Patients who are CR, PR, REGR, or SD will continue to obtain imaging at each clinically indicated follow up for up to *2 years from registration*.

13.4. Patients who develop PD or withdraw from further follow up assessments will be followed for survival only.

13.5. Inevaluable patients: If a patient fails to complete therapy for reasons other than toxicity, the patient will be regarded as inevaluable and will be replaced.

For peer review only

14. Body Fluid Biospecimens

**Correlative Study (Section 4.0 for more information)**	**Mandatory or Optional**	**Blood or Body Fluid being Collected**	**Type of Collection Tube (color of tube top)**	**Volume to collect per tube (# of tubes to be collected)**	**Visit 1** ***(Screening)***	**Visit 2** ***(Pre-Initial TPE)***	**Visit 3** ***(Post-Final TPE***	**Visit 4** ***(Pre-2nd IO)***	**Process at site? (Yes or No)**	**Temperature Conditions for Storage /Shipping**
Pharmacokinetics, Circulating EVs, PBMCs	Mandatory	Serum	Yellow	6mL (6)	X	X	X	X	No	Ambient
Waste	Mandatory	Plasma exchange waste product	Waste bag	Up to 800cc (1)		X			No	Ambient

For peer review only

14.1. Collection and Processing

Specimens will be collected at the following time points:

- #1 Pre-SBRT (or one week prior to immunotherapy)
- #2 Pre-Initial TPE (for selected patients with sPD-L1 levels above cutoff)
- #3 Post-Final TPE
- #4 2-3 week follow-up at standard clinical appointment prior to next round of immunotherapy

14.1.1. 6-6ML tubes, 36 ml will be collected in citrate yellow-toped tubes at each timepoint. The tubes should be gently inverted 7 to 10 times to thoroughly mix the samples. These samples will be processed within 7 days of collection for sPD-L1, extracellular vesicles, and other metabolites using ELISA and flow cytometry per lab protocols. All other samples must be labeled for processing within one week. Citrate tubes will be provided for this project.

For peer review only

15. 0 Drug Information

15.1. **Pembrolizumab (Keytruda®)** – Standard of care treatment allowed in this study.

15.1.1. Approved for treatment of advanced melanoma (unresectable or metastatic)

15.1.2. Dose 200mg IV every 3 weeks until disease progression or unacceptable toxicity.

15.1.3. Class: Immune checkpoint inhibitor (ICI), monoclonal programmed death receptor-1 (PD-1) blocking human monoclonal antibody.

15.1.4. FDA approval for recurrent/metastatic cervical cancer, advanced endometrial carcinoma, recurrent locally advanced/metastatic esophageal or gastric cancer, unresectable/recurrent/metastatic head and neck squamous cell cancer, advanced hepatocellular carcinoma, relapsed/refractory classical Hodgkin lymphoma, recurrent/metastatic Merkel cell carcinoma, stage III or IV non-small cell lung cancer, relapsed/refractory mediastinal large B-cell lymphoma, advanced renal cell carcinoma, metastatic small cell lung cancer, high-risk locally advanced/metastatic urothelial carcinoma, and unresectable/metastatic microsatellite instability-high cancers of any kind [19].

15.1.5. Adverse events related to immune-related treatments (irAE) will be collected at each follow-up visit. Serious irAE include pneumonitis, colitis, hepatitis, nephritis/renal dysfunction, hypothyroidism/hyperthyroidism.

15.1.6. Pharmacokinetics: administered intravenously, clearance difference not considered to be clinically important.

15.2. **Nivolumab (Opdivo®)**– Standard of care treatment allowed in this study.

15.2.1. Approved for treatment of advanced melanoma (unresectable or metastatic)

15.2.2. Dose 240mg IV every 2 weeks until disease progression or unacceptable toxicity.

15.2.3. Class: Immune checkpoint inhibitor (ICI), monoclonal programmed death receptor-1 (PD-1) blocking human monoclonal antibody.

15.2.4. FDA approval for MSI-high colorectal cancer, recurrent/metastatic head and neck SCC, hepatocellular carcinoma, classical Hodgkin lymphoma,

For peer review only

unresectable/metastatic melanoma, advanced renal cell carcinoma, metastatic small cell lung cancer, and locally advanced/metastatic urothelial carcinoma [20].

15.2.5. Adverse events related to immune-related treatments (irAE) will be collected at each follow-up visit. Serious irAE include pneumonitis, colitis, hepatitis, nephritis/renal dysfunction, hypothyroidism/hyperthyroidism.

15.2.6. Pharmacokinetics: administered intravenously, clearance unchanged by dose. 30-minute infusion comparable to 60-minute infusion. Clearance increased by 20% in the presence of anti-nivolumab antibodies.

For peer review only

16.0 Statistical Considerations and Methodology

16.1 Overview and Study Design:

This is a feasibility trial that seeks to assess feasibility and kinetics of the plasma exchange approach in these patients. In addition, we plan to assess response rate, overall survival, progression-free survival, adverse events, and correlative analysis.

16.1.1 Primary Endpoint

The primary endpoint of this trial is to assess the feasibility and kinetics for this treatment approach. Feasibility will be assessed by being able to complete the study accrual in a reasonable time period, where if we cannot enroll all 20 patients within 2 years, we'll assume this approach isn't feasible. Kinetics of sPD-L1 production in melanoma will be asssessed in an exploratory fashion by assessing the change in the sPD-L1 levels between different time points of interest. Since this feasibility study is small and exploratory, no power calculations are needed. Additional comparator substances or potentially pathogenic tumor substances will also be measured for comparison.

16.1.2 Sample Size

Eighteen eligible patients will be accrued onto this feasibilty study. We anticipate accruing an additional 2 patients to account for ineligibility, cancellation, major treatment violation, or other reasons. Therefore, maximum accrual is expected to be 20 registered patients. To accrue 20 patients that exhibit sPD-L1 above our cutoff (usually ~35% of patients), we expect to pre-register around 60 patients total.

16.1.3 Accrual Time and Study Duration

The anticipated registration rate is approximately 2 patients per month. Therefore, the accrual period for this study is expected to be approximately 10 months. The final analysis can begin approximately 24 months after the trial begins, i.e. as soon as the last patient has been followed for at least 12 months plus time for data entry and clean-up.

16.2 Data and Safety Monitoring

The principal investigator(s) and the study statistician will review the study at least monthly to identify accrual, adverse event, and any endpoint problems that might be developing. The trial is monitored continually by the study team who are notified of every grade 4 and 5 event in real time. The Mayo Clinic Cancer Center (MCCC) Data Safety Monitoring Board (DSMB) is responsible for reviewing accrual and safety data for this trial at least twice a year, based on reports provided by the MCCC Statistical Office. Any safety

For peer review only

issues requiring protocol changes are communicated through protocol amendments.

Adverse Event Stopping Rule: Based on previous experience with this disease, we expect approximately 10% of patients to experience Grade 4+ adverse events. If at any time, 3 of the first 10 registered patients (or 30% or more beyond 10 registered patients) have experienced any Grade 4 or 5 adverse event (at least possibly related to the study treatment), accrual to the study will be suspended to allow for a full review of the data. Each grade 5 event will be reviewed on a case by case basis in a real time fashion to determine whether study accrual should be suspended. After consideration by the study team [ie, Study Chair(s), Statistician, Operations Office, etc] and consultation with representatives at the primary Internal Review Board (IRB) affiliated with the Operations Office, a decision will be made as to whether and how the study will proceed.

16.3. Analysis Plan

16.3.1. Primary Endpoint

For feasibility, we'll just keep track of the accrual and accrual rate. We're hoping for fast accrual, where we can register up to 2 patients per month. Feasibility will be assessed by being able to complete the study accrual in a reasonable time period, where if we can't register all 20 patients within 2 years, we'll assume this approach isn't feasible.

We'll also determine the kinetics of sPD-L1 removal and regeneration by plasma exchange in patients with melanoma. Graphical methods and descriptive statistics will be used to explore this endpoint. The Wilcoxon Signed-Rank test will be used to assess the change in the sPD-L1 levels over time across the different timepoints of interest (pre vs. post initial plasma exchange therapy; post plasma vs. 2 to 3-week follow-up; pre vs. 2 to 3-week follow-up timepoint). The hypothesis is that the sPD-L1 levels will decrease with the use of plasma exchange treatment. The hope is that this decrease will be maintained throughout the first 2 to 3 weeks of follow-up and beyond. Other potentially pathogenic tumor factors will also be measured and compared for correlated studies.

16.3.2. Definition and Analyses of Secondary Endpoints

16.3.2.1. Overall Response rate is the proportion of patients with a tumor response (PR or CR at least 4 weeks apart). All patients meeting the eligibility criteria who have signed a consent form and have begun treatment will be evaluable for the response rate.

For peer review only

16.3.2.2. Progression-free survival (PFS) is defined as the time from registration to the first of either disease progression or death from any cause. Patients who receive the study drug, but then never return for an evaluation will be censored on their last follow-up date. PFS will be estimated using the method of Kaplan-Meier.

16.3.2.3. Overall survival (OS) is defined as the time from registration to death from any cause. OS will be estimated using the method of Kaplan-Meier.

16.3.3. Adverse Events

All patients that have intitiated treatment will be considered evaluable for sadverse event (AE) analyses. The maximum grade for each type of AE will be recorded for each patient, and frequency tables will be reviewed to determine AE patterns.

16.3.4. Analysis of Translational Component

For this correlative analysis, we will determine the effects of plasma exchange on immune cell function, observe the kinetics of extracellular vesicles (EVs) after plasma exchange in patients with melanoma, and associate the kinetics with clinical outcome data (RR, OS, PFS). Associations of categorical data will be assessed using Fisher exact tests. Associations of continuous data with binary data will be assessed using standard Wilcoxon Rank-Sum tests. Assessment of the change in continuous data over time will be done using the Wilcoxon Signed-Rank test. Kaplan-Meier methods and the log-rank test will be used for time-to-event data. This translational study is considered exploratory and hypothesis generating due to the small proposed sample size for this study.

16.4. Inclusion of Women and Minorities

16.4.1. Study availability

This study will be available to all eligible patients, regardless of gender, race or ethnic origin.

16.4.2. Differential effects by race or ethnicity

There is no information currently available regarding differential effects of this regimen in subsets defined by gender, race or ethnicity, and there is no reason to expect such differences to exist. Therefore, although the planned analysis will, as always, look for differences in treatment effect based on racial groupings, the sample size is not increased in order to provide additional power for subset analyses.

16.4.3. Study population

For peer review only

Based on prior studies involving similar disease sites, we expect about 20% of patients will be classified as minorities by race and 5% will be women. Expected sizes (per study design) of racial by gender subsets are shown in the following table:

Accrual Estimates by Gender/Ethnicity/Race

Accrual Targets			
	Sex/Gender		
**Ethnic Category**	**Females**	**Males**	**Total**
Hispanic or Latino	2	2	4
Not Hispanic or Latino	8	8	16
**Ethnic Category: Total of all subjects**	**10**	**10**	**20**
**Racial Category**			
American Indian or Alaskan Native	0	0	0
Asian	1	1	2
Black or African American	1	1	2
Native Hawaiian or other Pacific Islander	0	0	0
White	8	8	16
**Racial Category: Total of all subjects**	**10**	**10**	**20**

Ethnic Categories: **Hispanic or Latino** – a person of Cuban, Mexican, Puerto Rican, South or Central American, or other Spanish culture or origin, regardless of race. The term "Spanish origin" can also be used in addition to "Hispanic or Latino."

Not Hispanic or Latino

Racial Categories: **American Indian or Alaskan Native** – a person having origins in any of the original peoples of North, Central, or South America, and who maintains tribal affiliations or community attachment.

Asian – a person having origins in any of the original peoples of the Far East, Southeast Asia, or the Indian subcontinent including, for example, Cambodia, China, India, Japan, Korea, Malaysia, Pakistan, the Philippine Islands, Thailand, and Vietnam. (Note: Individuals from the Philippine Islands have been recorded as Pacific Islanders in previous data collection strategies.)

Black or African American – a person having origins in any of the black racial groups of Africa. Terms such as "Haitian" or "Negro" can be used in addition to "Black or African American."

Native Hawaiian or other Pacific Islander – a person having origins in any of the original peoples of Hawaii, Guam, Samoa, or other Pacific Islands.

White – a person having origins in any of the original peoples of Europe, the Middle East, or North Africa.

For peer review only

17. Pathology Considerations/Tissue Biospecimens

No tissue Biospecimens.

For peer review only

18. Records and Data Collection Procedures

18.1 Data Handling and Record Keeping

18.1.1 Confidentiality

Information about study subjects will be kept confidential and managed according to the requirements of the Health Insurance Portability and Accountability Act of 1996 (HIPAA). Those regulations require a signed subject authorization informing the subject of the following:

- What protected health information (PHI) will be collected from subjects in this study
- Who will have access to that information and why
- Who will use or disclose that information
- The rights of a research subject to revoke their authorization for use of their PHI.

In the event that a subject revokes authorization to collect or use PHI, the investigator, by regulation, retains the ability to use all information collected prior to the revocation of subject authorization. For subjects that have revoked authorization to collect or use PHI, attempts should be made to obtain permission to collect at least vital status (long term survival status that the subject is alive) at the end of their scheduled study period.

18.1.2 Source Documents

Source data is all information, original records of clinical findings, observations, or other activities in a clinical trial necessary for the reconstruction and evaluation of the trial. Source data are contained in source documents. Examples of these original documents, and data records include: hospital records, clinical and office charts, laboratory notes, memoranda, subjects' diaries or evaluation checklists, pharmacy dispensing records, recorded data from automated instruments, copies or transcriptions certified after verification as being accurate and complete, microfiches, photographic negatives, microfilm or magnetic media, x-rays, subject files, and records kept at the pharmacy, at the laboratories, and at medico-technical departments involved in the clinical trial. Source documents are kept in a secure location that is locked and requires approved access.

18.1.3 Case Report Forms

The study case report form (CRF) is the primary data collection instrument for the study. All data requested on the CRF must be recorded. All missing data must be explained. All data will be entered into electronic case report forms (eCRF's)

For peer review only

through the Medidata Rave system. Case report forms will be automatically rolled out based on a predetermined and visit-based schedule to improve study staff workflow and data quality. Data will be exported nightly to a secure FTP for analysis and reporting.

18.1.4 Records Retention

The investigator will maintain records and essential documents related to the conduct of the study. These will include subject case histories and regulatory documents.

The investigator will retain the specified records and reports as outlined in the Mayo Clinic Research Policy Manual–"Retention of and Access to Research Data Policy" http://mayocontent.mayo.edu/research-policy/MSS_669717

For peer review only

19. Budget

19.1. Costs charged to patient: routine clinical care including SBRT, pembrolizumab or nivolumab depending on physician preference.

19.2. Tests to be research funded: Therapeutic Plasma Exchange (TPE), sPD-L1 ELISA, EV nanoflow, any other tests of potentially pathogenic or comparator substances in blood.

For peer review only

20. References

[1] M. Aitken, M. Kleinrock, A. Simorellis, and D. Nass, “Global Oncology Trends 2018,” London, 2018.

[2] A. Haslam and V. Prasad, “Estimation of the Percentage of US Patients With Cancer Who Are Eligible for and Respond to Checkpoint Inhibitor Immunotherapy Drugs,” *JAMA Netw. open*, vol. 2, no. 5, p. e192535, May 2019.

[3] J. S. O’Donnell, G. V Long, R. A. Scolyer, M. W. L. Teng, and M. J. Smyth, “Resistance to PD1/PDL1 checkpoint inhibition.,” *Cancer Treat. Rev.*, vol. 52, pp. 71–81, Jan. 2017.

[4] J. Zhou *et al.*, “Soluble PD-L1 as a Biomarker in Malignant Melanoma Treated with Checkpoint Blockade.,” *Cancer Immunol. Res.*, vol. 5, no. 6, pp. 480–492, 2017.

[5] K. M. Mahoney *et al.*, “A secreted PD-L1 splice variant that covalently dimerizes and mediates immunosuppression,” *Cancer Immunol. Immunother.*, pp. 1–12, Dec. 2018.

[6] J. et al Orme, “ADAM10 and ADAM17 cleave PD-L1 to mediate PD-(L)1 inhibitor resistance.,” *Oncoimmunology*, vol. In press, p. DOI 10.1080/2162402X.2020.1744980, 2020.

[7] Y. Romero, R. Wise, and A. Zolkiewska, “Proteolytic processing of PD-L1 by ADAM proteases in breast cancer cells,” *Cancer Immunol. Immunother.*, vol. 69, no. 1, pp. 43–55, Jan. 2020.

[8] G. Chen *et al.*, “Exosomal PD-L1 contributes to immunosuppression and is associated with anti-PD-1 response,” *Nature*, vol. 560, no. 7718, pp. 382–386, Aug. 2018.

[9] M. Poggio *et al.*, “Suppression of Exosomal PD-L1 Induces Systemic Anti-tumor Immunity and Memory,” *Cell*, vol. 177, no. 2, pp. 414-427.e13, Apr. 2019.

[10] Y. Li *et al.*, “Serum sPD-1 and sPD-L1 as Biomarkers for Evaluating the Efficacy of Neoadjuvant Chemotherapy in Triple-Negative Breast Cancer Patients.,” *Clin. Breast Cancer*, vol. 19, no. 5, pp. 326-332.e1, Oct. 2019.

[11] H. Ha *et al.*, “Soluble programmed death-ligand 1 (sPDL1) and neutrophil-to-lymphocyte ratio (NLR) predicts survival in advanced biliary tract cancer patients treated with palliative chemotherapy.,” *Oncotarget*, vol. 7, no. 47, pp. 76604–76612, Nov. 2016.

[12] Y. Fan *et al.*, “Exosomal PD-L1 Retains Immunosuppressive Activity and is Associated with Gastric Cancer Prognosis.,” *Ann. Surg. Oncol.*, vol. 26, no. 11, pp. 3745–3755, Oct. 2019.

[13] K. Ando *et al.*, “Plasma Levels of Soluble PD-L1 Correlate With Tumor Regression in Patients With Lung and Gastric Cancer Treated With Immune Checkpoint Inhibitors.,” *Anticancer Res.*, vol. 39, no. 9, pp. 5195–5201, Sep. 2019.

[14] A. Costantini *et al.*, “Predictive role of plasmatic biomarkers in advanced non-small cell lung cancer treated by nivolumab.,” *Oncoimmunology*, vol. 7, no. 8, p. e1452581, 2018.

[15] Y. Okuma *et al.*, “Soluble Programmed Cell Death Ligand 1 as a Novel Biomarker for Nivolumab Therapy for Non-Small-cell Lung Cancer.,” *Clin. Lung Cancer*, vol. 19, no. 5, pp. 410-417.e1, 2018.

[16] J. et al Orme, “Plasma exchange clears soluble PD-L1 and extracellular vesicles in vivo,” *Manuscr. Prep.*, 2020.

For peer review only

[17] X. Frigola *et al.*, "Identification of a soluble form of B7-H1 that retains immunosuppressive activity and is associated with aggressive renal cell carcinoma.," *Clin Cancer Res. Clin Cancer Res. April*, vol. 1, no. 177, pp. 1915–1923, 2011.

[18] E. A. Eisenhauer *et al.*, "New response evaluation criteria in solid tumours: Revised RECIST guideline (version 1.1)," *Eur. J. Cancer*, vol. 45, no. 2, pp. 228–247, Jan. 2009.

[19] "Keytruda: Highlights of Presciribing Information," 2017.

[20] "Nivolumab: Highlights of Prescribing Information," 2018.

For peer review only

Appendix I ECOG Performance Status

ECOG PERFORMANCE STATUS*

Grade	ECOG
0	Fully active, able to carry on all pre-disease performance without restriction
1	Restricted in physically strenuous activity but ambulatory and able to carry out work of a light or sedentary nature, e.g., light house work, office work
2	Ambulatory and capable of all selfcare but unable to carry out any work activities. Up and about more than 50% of waking hours
3	Capable of only limited selfcare, confined to bed or chair more than 50% of waking hours.
4	Completely disabled. Cannot carry on any selfcare. Totally confined to bed or chair.
5	Dead

*As published in Am. J. Clin. Oncol.:
Oken, M.M., Creech, R.H., Tormey, D.C., Horton, J., Davis, T.E., McFadden, E.T., Carbone, P.P.: Toxicity And Response Criteria Of The Eastern Cooperative Oncology Group. Am J Clin Oncol 5:649-655, 1982.

The ECOG Performance Status is in the public domain therefore available for public use. To duplicate the scale, please cite the reference above and credit the Eastern Cooperative Oncology Group, Robert Comis M.D., Group Chair.

From http://www.ecog.org/general/perf_stat.html

Adverse Event Form

STUDY NAME

Site Name:__________________________ Pt_ID:__________________________	This form is cumulative and captures adverse events of a single participant throughout the study.

Severity	Study Intervention Relationship	Action Taken Regarding Study Intervention	Outcome of AE	Expected	Serious Adverse Event (SAE)
1 = Mild<br>2 = Moderate<br>3 = Severe<br>4 = Life-Threatening	0 = Not related<br>1 = Unlikely related<br>2 = Possibly related<br>3 = Probably related<br>4 = Definitely related	0 = None<br>1 = Dose modification<br>2 = Medical Intervention<br>3 = Hospitalization<br>4 = Intervention discontinued<br>5 = Other	1 = Resolved<br>2 = Recovered with minor sequelae<br>3 = Recovered with major sequelae<br>4 = Ongoing/Continuing treatment<br>5 = Condition worsening<br>6 = Death<br>7 = Unknown	1 = Yes<br>2 = No	1 = Yes<br>2 = No<br>(if yes, complete SAE form)

At end of study only: Check this box if participant had no adverse events ☐ None

Adverse Event	Start Date	Stop Date	Severity	Relationship	Action Taken	Outcome of AE	Expected?	SAE?

Page __ of __

Adverse Event Form Version 2.0

Rescuing Cancer Immunity by Plasma Exchange in Metastatic Melanoma (ReCIPE-M1): Protocol for a single-institution, open-label safety trial of plasma exchange to clear sPD-L1 for immunotherapy

Journal:	*BMJ Open*
Manuscript ID	bmjopen-2021-050112.R2
Article Type:	Protocol
Date Submitted by the Author:	25-Feb-2022

Complete List of Authors:	Davidson, Tara M.; Mayo Clinic, Department of Internal Medicine<br>Foster, Nathan; Mayo Clinic, Department of Quantitative Health Sciences<br>Lucien, Fabrice; Mayo Clinic, Department of Urology<br>Svetomir, Markovic; Mayo Clinic, Division of Medical Oncology<br>Dong, Haidong; Mayo Clinic, Department of Urology<br>Winters, Jeffrey L.; Mayo Clinic, Department of Pathology and Laboratory Medicine<br>Park, Sean S.; Mayo Clinic, Radiation Oncology<br>Orme, Jacob; Mayo Clinic, Division of Medical Oncology

**Primary Subject Heading**:	Oncology
Secondary Subject Heading:	Oncology, Urology
Keywords:	ONCOLOGY, IMMUNOLOGY, Radiobiology < RADIOLOGY & IMAGING, Prostate disease < UROLOGY

Clinical trial design: BMJ Open

Title: Rescuing Cancer Immunity by Plasma Exchange in Metastatic Melanoma (ReCIPE-M1): Protocol for a single-institution, open-label safety trial of plasma exchange to clear sPD-L1 for immunotherapy

Authors: Tara M. Davidson[1]; Nathan Foster[2]; Fabrice Lucien[3]; Svetomir Markovic[4]; Haidong Dong[3]; Jeffrey L. Winters[5]; Sean S. Park[6,*]; Jacob J. Orme[4,*]

[1]Department of Internal Medicine, Mayo Clinic, Rochester, MN

[2]Department of Quantitative Health Sciences, Mayo Clinic, Rochester, MN

[3]Department of Urology, Mayo Clinic, Rochester, MN

[4]Division of Medical Oncology, Mayo Clinic, Rochester, MN

[5]Department of Pathology and Laboratory Medicine, Mayo Clinic, Rochester, MN

[6]Department of Radiation Oncology, Mayo Clinic, Rochester, MN

*These authors contributed equally

Running title: TPE for melanoma immunotherapy

Keywords: PD-L1, PD-1, soluble PD-L1, sPD-L1

Financial support: Support: NCI CA259236, Lawrence W. and Marilyn W. Matteson Award

For peer review only

For peer review only - http://bmjopen.bmj.com/site/about/guidelines.xhtml

COI: Intellectual property has been filed addressing discoveries disclosed in this manuscript. The authors report no other relevant conflicts of interest.

Corresponding Author:

Jacob Orme, M.D. Ph.D.

200 1st St NW

Rochester, MN 55902

Phone: 507-284-2511

Email: orme.jacob@mayo.edu

Word count: 2,237

Total figures and tables: 2 figures, 1 table

For peer review only

For peer review only - http://bmjopen.bmj.com/site/about/guidelines.xhtml

ABSTRACT

Background: Patients with metastatic melanoma rely on PD-(L)1 immunotherapy, but only one third of patients experience treatment response and all initial responders eventually develop resistance. Tumor-derived extracellular vesicles expressing PD-L1 (evPD-L1) and soluble PD-L1 (sPD-L1) in peripheral blood of patients with melanoma limit PD-(L)1 immunotherapy and correlate with poor survival. Therapeutic plasma exchange (TPE) removes immunosuppressive evPD-L1 and sPD-L1. We hypothesize that TPE may rescue and restore anti-melanoma immunity.

Methods: In this two-arm study (NCT04581382), 60 patients with metastatic melanoma progressing on checkpoint inhibition will be accrued. All patients will receive radiotherapy on days 1-5 (at least one measurable lesion will not be irradiated) and ongoing checkpoint inhibition on day 8 and every 2-3 weeks per standard of care. Patients with baseline sPD-L1 level ≥1.7 ng/ml and adequate clinical capacity will be enrolled in the TPE Intervention Arm and receive TPE on days 5-7 in addition to standard of care radiotherapy and immunotherapy. Other patients will remain in the Standard of Care Arm.

The primary endpoint of the study is to evaluate safety. Secondary endpoints include kinetics of sPD-L1 and evPD-L1 and clinical response by RECIST criteria.

Ethics and Dissemination: This trial has been approved by the Mayo Clinic IRB. It will assess the safety and feasibility of TPE in improving outcomes for PD-(L)1 inhibitor immunotherapy in melanoma. Data will be maintained on a secure database with de-identified patient information. Data will be shared on publication in a peer-reviewed

For peer review only - http://bmjopen.bmj.com/site/about/guidelines.xhtml

journal without the aid of professional writers. If successful, this trial will lay the ground for Phase II studies that will include cancer treated with PD-(L)1 inhibitors which may benefit from TPE such as renal, bladder, and lung cancers.

ARTICLE SUMMARY

Strengths and limitations of this study:

- Screens 60 patients with metastatic melanoma progressing despite checkpoint inhibition
- Evaluates the safety of TPE and kinetics of immunomodulatory molecules removed by TPE in vivo in melanoma
- Involves no randomization to intervention versus control arm in this early study

For peer review only

For peer review only - http://bmjopen.bmj.com/site/about/guidelines.xhtml

1. INTRODUCTION

The first immunotherapy with interferon was approved by the Food and Drug Administration (FDA) in 1986.[1] Since then, leveraging the body's own immune system to target and kill malignant cells has become the fastest-growing therapeutic approach to cancer worldwide.[2] While early immunotherapies focused on providing immunostimulatory cytokines (*e.g.,* pegilodecakin), more recent approaches have leveraged vaccination (*e.g.,* MAGE-A3), transplant of primed anti-tumor CD8+ T cells (*i.e.,* CAR-T therapy), and inhibiting immune checkpoint signals (*e.g.,* Programmed Death 1 [PD-1] and Programmed Death Ligand 1 [PD-L1] inhibitors like pembrolizumab, nivolumab, and atezolizumab).[3]

The latter approach with PD-(L)1 inhibitors has shown spectacular results in many indications, leading to first-line approvals of checkpoint inhibitor immunotherapy in renal cell carcinoma, non-small cell lung cancer, melanoma, and others.[4-6] Unfortunately, even in these highly promising settings less than half of patients have objective response to PD-(L)1 inhibitor therapy alone.[7] For instance, only about one in three melanomas respond to PD-(L)1 inhibitor pembrolizumab.[6] Intensive research is focused on identifying causes for intrinsic resistance to PD-(L)1 inhibitors.

PD-L1 is classically thought to be a membrane-bound ligand. Recently, however, three additional functional forms of *extracellular* PD-L1 have been identified. Soluble PD-L1 can be produced by transcribed PD-L1 splice variants, of which four variants have been identified in melanoma.[8,9] Metalloproteases ADAM10 and ADAM17 can also cleave the PD-L1 ectodomain from tumor cells and extracellular vesicles (EVs) to form sPD-L1.[10] Lastly, tumor cells release extracellular vesicles bearing intact PD-L1 (evPD-L1).[11]

For peer review only

sPD-L1 levels are significantly higher in patients with metastatic melanoma at baseline compared to healthy volunteers and elevated levels predict inferior outcomes.[8,10,12] This implies that the same inflammatory/immune response that is known to play a role in PD-L1 expression also increases the rate of extracellular PD-L1 production. In one study, PD-L1 cell surface expression paralleled sPD-L1 secretion in response to cytokines in melanoma cells.[8] Some cancer cells secrete a majority of their PD-L1 on exosomes, even with only minimal amounts of cellular PD-L1 found.[9,11] The leading hypothesis is that the tumor microenvironment, including non-neoplastic cells, may also be contributing to the production of sPD-L1 and evPD-L1. And although radiotherapy has been proposed as a method to induce anti-tumor immunity, studies have also shown that radiation increases the production of extracellular PD-L1 through inflammatory cytokines and other mechanisms.[13,14]

While the roles of these extracellular PD-L1 forms continue to be elucidated, the results of sPD-L1 and evPD-L1 signaling are clear from both in vitro and in vivo studies: sPD-L1 and evPD-L1 outcompete PD-(L)1 inhibitors, initiate apoptosis in CD8+ T cells, and cause systemic immunosuppression by limiting the ability of healthy peripheral blood mononuclear cells to kill tumor cells (Figure 1A). [9,10,15] Studies in multiple malignancies find a negative relationship between high sPD-L1 and outcomes.[16,17,18, 19-27] In melanoma, Zhou et al showed that high pretreatment levels of sPD-L1 were associated with increased likelihood of progressive disease in patients with melanoma treated by CTLA-4 or PD-1 blockade.[8]

We recently explored the potential of therapeutic plasma exchange (TPE) in removing sPD-L1 and evPD-L1.[28] In brief, TPE is a procedure for passing blood through an

For peer review only - http://bmjopen.bmj.com/site/about/guidelines.xhtml

apheresis machine to separate plasma from cellular components and is commonly used clinically for removal of deleterious antibodies.[29] This has been described as a method to “clean” or “flush out” the blood. Plasma containing the offending substance is replaced in this procedure with up to 30% crystalloid in the form of normal saline and either donor plasma or 5% human albumin derived from plasma donation. In our previous report, each session of TPE reduced sPD-L1 and evPD-L1 by 70.8% and 73.1%, respectively.[28] This represents the first clinical intervention to remove either of these substances from circulation in vivo. It is hypothesized that the removal of these extracellular forms of PD-L1 may re-sensitize tumors to immune checkpoint inhibitor treatment (Figure 1B).

Despite these important discoveries, several crucial questions remain unanswered. First, while TPE is commonly prescribed in the outpatient setting, it has not been studied specifically in cancer treated with immunotherapy. Second, the kinetics of sPD-L1 and evPD-L1 in patients with melanoma have not previously been determined. Pilot studies to establish the safety profile and durability of TPE-mediated changes in melanoma are warranted.

2. METHODS

2.1. Overview of study design

This feasibility trial (NCT04581382) will assess (1) the safety of TPE through adverse events monitoring at 1 and 6 months post-therapy (primary outcome), (2) the kinetics of evPD-L1, sPD-L1, and related immunosuppressive metabolites immediately and 2-3

For peer review only - http://bmjopen.bmj.com/site/about/guidelines.xhtml

weeks after TPE, and (3) the relative response of patients to combined therapy through response rate, overall survival, and progression-free survival.

2.2. Recruitment, screening procedures and treatment arms

Patients with metastatic melanoma progressing on standard of care on any PD-(L)1 inhibitor are eligible for this study (Table 1, Figure 2). Potentially eligible patients will be approached by the clinical trial coordinator and provide informed consent prior to a baseline screening blood draw. 60 patients will be screened from from December 2020 through October 2023 with an accrual goal of 20 patients to the TPE Intervention Arm. Inclusion criteria comprise age (18 or older), histologically confirmed melanoma, measurable disease per RECIST criteria, ECOG status of three or better, sPD-L1 levels 1.7ng/ml or greater, and feasible vascular access. Exclusion criteria include pregnancy or nursing. Also excluded are those who consume biotin supplementation as it interferes with accurate measurement of sPD-L1.

Enrolled patients with sPD-L1 level at or above 1.7ng/ml will be enrolled in the Intervention Arm where clinical availability of the procedure allows. Other patients will be enrolled in the Standard of Care Arm. All data will be collected in a deidentified fashion in a password-protected database.

2.3. Study interventions

All patients will receive treatment with any PD-(L)1 inhibitor per clinician preference, including but not limited to the continuation of the existing checkpoint inhibitor, escalation to ipilimumab and nivolumab, or switching to an alternative checkpoint inhibitor. Other concomitant therapies will be allowed on the trial. Additional treatment

For peer review only - http://bmjopen.bmj.com/site/about/guidelines.xhtml

comprises radiotherapy on days 1-5 and PD-(L)1 inhibitor treatment on day 8 and every 2-3 weeks thereafter as clinically indicated. Preferred targets for radiotherapy will be symptomatic lesions or lesions that can cause pain, fracture, or other symptoms in the near future. Patients in the Intervention Arm, in addition to the above, will undergo serial blood draws prior to the first TPE session, after the last TPE session and just prior to the next dose of immunotherapy. We will assess the kinetics of sPD-L1 and evPD-L1 at these timepoints.

TPE will be performed using centrifugation-based cell separators, either the Fenwal Amicus (Fresenius KABI USA LLC, Lake Zurich IL, USA) or the Spectra Optia (Terumo BCT Inc, Lakewood CO, USA). For each patient, a single plasma volume was exchanged using either peripheral IVs (preferred) or central lines for vascular access if a central line is present. Due to the possibility of sPD-L1 or PD-L1-positive EVs present in donor plasma, donor plasma (*i.e.,* fresh frozen plasma, FFP) will not be used as a replacement fluid. Rather, 5% normal serum human albumin with up to 500 ml of normal saline will be used as the replacement. The volume used is determined by the device based upon patient gender, height, weight, and hematocrit. Anticoagulation will consist of either 500 ml of acid citrate dextrose solution A (ACD-A) or 500 ml of ACD-A with 5000 units of unfractionated heparin. Anticoagulant to blood ratios will be 1:13 when ACD-A is used and 1:26 when ACD-A/Heparin is used. Patients will not receive routine electrolyte replacement but 10 ml of 10% calcium gluconate will be administered by slow IV push for signs and symptoms of hypocalcemia related to the ACD-A anticoagulant.

For peer review only - http://bmjopen.bmj.com/site/about/guidelines.xhtml

Toxicity assessment using CTCAE version 5.0 will be assessed within 7 days prior to registration as baseline in addition to daily during TPE and 2 weeks post first dose of immunotherapy if receiving nivolumab or 3 weeks post first dose of immunotherapy if receiving pembrolizumab as well as 3 months post-TPE. Safety will also be assessed during any clinically indicated follow-up until progressive disease, death or up to 2 years after registration.

Imaging will be obtained as standard of care and is not stipulated by the study. CT, PET, and/or MRI scans can be used as clinically indicated. Imaging to assess tumor response will be based on RECIST/PERCIST criteria (v1.1) until progression, death or up to 2 years after registration. At least one metastatic lesion will not be radiated to assess response per RECIST and/or PERCIST criteria for those in the Intervention Arm. All oligo-progressive lesions will be radiated for patients in the Standard of Care Arm while on standard-of-care immunotherapy. As patients who progress on immunotherapy are enrolled in this trial, partial or complete response of > 20% will be considered as a positive signal to proceed to a phase II trial. Study materials and procedures were approved by the Mayo Clinic IRB (approval #19-008055).

2.4 Measuring sPD-L1 and evPD-L1 levels

For measurements of sPD-L1, ELISA will be performed as previously published.[18] Both secreted splice variant and shed sPD-L1 are reliably detected by this ELISA. In brief, paired mouse IgG2 monoclonal antibody clones H1A and B11 against extracellular human PD-L1 will be utilized in a capture-detection plate assay using a standard biotinylated antibody and streptavidin-HRP detection method. This assay is specific for sPD-L1 and does not exhibit cross reactivity to other B7-H homologues, nor to evPD-L1.

For peer review only - http://bmjopen.bmj.com/site/about/guidelines.xhtml

For peer review only

Concentrations are determined by optical density measurements along a known standard curve of recombinant human PD-L1. ELISAs are performed by technicians blinded to the identity of the samples.

For measurements of evPD-L1, flow cytometry will be used as previously published.[30] In brief, plasma samples are centrifuged twice at 2000g to deplete platelets. Resultant platelet-free plasma is analyzed using an A60-Micro Plus Nanoscale Flow Cytometer (Apogee FlowSystems) gating for mid-intensity light angle light scatter and markers of interest. Anti-PD-L1 (Genentech: atezolizumab), antibodies are conjugated to fluorophores (Life Technologies: Alexa-647, PE phycoerythrin, and Alexa-488) and titrated prior to use. Nanoscale flow cytometer calibration is performed using a standard reference bead mix as previously published. Flow cytometry is performed by technicians blinded to the identity of the samples. Wilcoxon Signed-Rank test will be used to assess changes in sPD-L1 and evPD-L1 levels over time across the different timepoints of interest.

2.4. Endpoints

The primary endpoint of this trial is to assess the safety of TPE in patients with melanoma receiving PD-(L)1 immunotherapy. Adverse events will be collected according to CTCAE 5.0, and the protocol dictates that if three of the first ten patients experiences grade 4 or greater adverse events the study will be halted. The secondary endpoints of this trial are (i) the kinetics of sPD-L1 and evPD-L1 removal in patients with melanoma receiving PD-(L)1 immunotherapy, (ii) overall response rate as a proportion of patients with a tumor response of PR or CR at least 4 weeks apart, and (iii) progression-free and overall survival.

For peer review only - http://bmjopen.bmj.com/site/about/guidelines.xhtml

2.5. Statisical Methods

For this correlative analysis, we will determine the effects of plasma exchange on immune cell function, observe the kinetics of extracellular vesicles (EVs) after plasma exchange in patients with melanoma, and associate the kinetics with clinical outcome data (RR, OS, PFS). Associations of categorical data will be assessed using Fisher exact tests. Associations of continuous data with binary data will be assessed using standard Wilcoxon Rank-Sum tests. Assessment of the change in continuous data over time will be done using the Wilcoxon Signed-Rank test. Kaplan-Meier methods and the log-rank test will be used for time-to-event data. This translational study is considered exploratory and hypothesis generating due to the small proposed sample size for this study.

2.6. Data collection schedule

The principal investigator and study statistician will review the study at least monthly to identify accrual, adverse events, and any endpoint problems that might be developing. The trial is monitored continually by the study team who are notified of every grade 4 or 5 event in real time.

2.7. Patient and Public Involvement

No patient involved.

3. ETHICS AND DISSEMINATION

For peer review only - http://bmjopen.bmj.com/site/about/guidelines.xhtml

We hypothesize that TPE will reliably and safely reduce the levels of sPD-L1 and evPD-L1 in our study patients. We hypothesize that this will alleviate the immunosuppressive microenvironment caused by extracellular PD-L1 as well as minimize any competition for PD-(L)1 inhibitors prior to these study patients receiving their dose of PD-(L)1 inhibitor therapy. We hope to see improved response to these agents through this intervention. If successful, we will explore this further in other neoplasms in Phase II trials. While TPE does carry risks as an invasive procedure (including hypotension, infection and bleeding) and relies on adequate vascular access, it is not restricted to any particular tumor type and is widely available.

This trial has been approved by the Mayo Clinic IRB. Data will be maintained on a secure database with de-identified patient information. Data will be shared on publication in a peer-reviewed journal without the aid of professional writers. If successful, this trial will lay the ground for Phase II studies that will include cancer treated with PD-(L)1 inhibitors which may benefit from TPE such as renal, bladder, and lung cancers.

Any modifications to the protocol which may impact on the conduct of the study, potential benefit of the patient or may affect patient safety, including changes of study objectives, study design, patient population, sample sizes, study procedures, or significant administrative aspects will require a formal amendment to the protocol. Such amendment will be approved by the Mayo Clinic IRB prior to implementation and notified to the health authorities in accordance with local regulations.

For peer review only - http://bmjopen.bmj.com/site/about/guidelines.xhtml

REFERENCES

1. Golomb HM, Jacobs A, Fefer A, et al. Alpha-2 interferon therapy of hairy-cell leukemia: a multicenter study of 64 patients. *J Clin Oncol.* 1986;4(6):900-905.
2. Dighe S. Immune Checkpoint Inhibitors (ICI) Market by Type (CTLA-4 Inhibitor, PD-1 Inhibitor, and PD-L1 Inhibitor) and Application (Lung Cancer, Bladder Cancer, Melanoma, Hodgkin Lymphoma, and Others). *https://wwwalliedmarketresearchcom/immune-check-point-inhibitors-market (2018).*
3. Dong H, Strome SE, Salomao DR, et al. Tumor-associated B7-H1 promotes T-cell apoptosis: a potential mechanism of immune evasion. *Nat Med.* 2002;8(8):793-800.
4. Hellmann MD, Paz-Ares L, Bernabe Caro R, et al. Nivolumab plus Ipilimumab in Advanced Non-Small-Cell Lung Cancer. *N Engl J Med.* 2019;381(21):2020-2031.
5. Rini BI, Plimack ER, Stus V, et al. Pembrolizumab plus Axitinib versus Sunitinib for Advanced Renal-Cell Carcinoma. *N Engl J Med.* 2019;380(12):1116-1127.
6. Robert C, Schachter J, Long GV, et al. Pembrolizumab versus Ipilimumab in Advanced Melanoma. *N Engl J Med.* 2015;372(26):2521-2532.
7. Haslam A, Prasad V. Estimation of the Percentage of US Patients With Cancer Who Are Eligible for and Respond to Checkpoint Inhibitor Immunotherapy Drugs. *JAMA Netw Open.* 2019;2(5):e192535.
8. Zhou J, Mahoney KM, Giobbie-Hurder A, et al. Soluble PD-L1 as a Biomarker in Malignant Melanoma Treated with Checkpoint Blockade. *Cancer Immunol Res.* 2017;5(6):480-492.
9. Poggio M, Hu T, Pai CC, et al. Suppression of Exosomal PD-L1 Induces Systemic Anti-tumor Immunity and Memory. *Cell.* 2019;177(2):414-427 e413.
10. Orme JJ, Jazieh KA, Xie T, et al. ADAM10 and ADAM17 cleave PD-L1 to mediate PD-(L)1 inhibitor resistance. *Oncoimmunology.* 2020;9(1):1744980.
11. Chen G, Huang AC, Zhang W, et al. Exosomal PD-L1 contributes to immunosuppression and is associated with anti-PD-1 response. *Nature.* 2018;560(7718):382-386.
12. Chen Y, Wang Q, Shi B, et al. Development of a sandwich ELISA for evaluating soluble PD-L1 (CD274) in human sera of different ages as well as supernatants of PD-L1+ cell lines. *Cytokine.* 2011;56(2):231-238
13. Ding XC, Wang LL, Zhu YF, et al. The Change of Soluble Programmed Cell Death-Ligand 1 in Glioma Patients Receiving Radiotherapy and Its Impact on Clinical Outcomes. *Front Immunol.* 2020;11:580335.
14. Kim HJ, Park S, Kim KJ, Seong J. Clinical significance of soluble programmed cell death ligand-1 (sPD-L1) in hepatocellular carcinoma patients treated with radiotherapy. *Radiother Oncol.* 2018;129(1):130-135.
15. Parry RV, Chemnitz JM, Frauwirth KA, et al. CTLA-4 and PD-1 receptors inhibit T-cell activation by distinct mechanisms. *Mol Cell Biol.* 2005;25(21):9543-9553.
16. Dronca RS, Harrington S, Jegapragasan M, et al. Association of soluble PD-L1 (sPD-L1) with decreased survival in metastatic melanoma. *Journal of Clinical Oncology.* 2017;35(7_suppl):4-4.

For peer review only - http://bmjopen.bmj.com/site/about/guidelines.xhtml

17. Dronca RS, Mansfield AS, Liu X, et al. Bim and soluble PD-L1 (sPD-L1) as predictive biomarkers of response to anti-PD-1 therapy in patients with melanoma and lung carcinoma. *Journal of Clinical Oncology.* 2017;35(15_suppl):11534-11534.
18. Frigola X, Inman BA, Lohse CM, et al. Identification of a Soluble Form of B7-H1 That Retains Immunosuppressive Activity and Is Associated with Aggressive Renal Cell Carcinoma. *Clinical Cancer Research.* 2011;17(7):1915-1923.
19. Costantini A, Julie C, Dumenil C, et al. Predictive role of plasmatic biomarkers in advanced non-small cell lung cancer treated by nivolumab. *Oncoimmunology.* 2018;7(8):e1452581.
20. Finkelmeier F, Canli Ö, Tal A, et al. High levels of the soluble programmed death-ligand (sPD-L1) identify hepatocellular carcinoma patients with a poor prognosis. *Eur J Cancer.* 2016;59:152-159.
21. Ha H, Nam AR, Bang JH, et al. Soluble programmed death-ligand 1 (sPDL1) and neutrophil-to-lymphocyte ratio (NLR) predicts survival in advanced biliary tract cancer patients treated with palliative chemotherapy. *Oncotarget.* 2016;7(47):76604-76612.
22. Okuma Y, Hosomi Y, Nakahara Y, Watanabe K, Sagawa Y, Homma S. High plasma levels of soluble programmed cell death ligand 1 are prognostic for reduced survival in advanced lung cancer. *Lung Cancer.* 2017;104:1-6.
23. Sideras K, de Man RA, Harrington SM, et al. Circulating levels of PD-L1 and Galectin-9 are associated with patient survival in surgically treated Hepatocellular Carcinoma independent of their intra-tumoral expression levels. *Scientific Reports.* 2019;9(1):10677.
24. Wang H, Wang L, Liu WJ, et al. High post-treatment serum levels of soluble programmed cell death ligand 1 predict early relapse and poor prognosis in extranodal NK/T cell lymphoma patients. *Oncotarget.* 2016;7(22):33035-33045.
25. Wang L, Wang H, Chen H, et al. Serum levels of soluble programmed death ligand 1 predict treatment response and progression free survival in multiple myeloma. *Oncotarget.* 2015;6(38):41228-41236.
26. Zhang J, Gao J, Li Y, et al. Circulating PD-L1 in NSCLC patients and the correlation between the level of PD-L1 expression and the clinical characteristics. *Thorac Cancer.* 2015;6(4):534-538.
27. Zheng Z, Bu Z, Liu X, et al. Level of circulating PD-L1 expression in patients with advanced gastric cancer and its clinical implications. *Chin J Cancer Res.* 2014;26(1):104-111.
28. Orme JJ, Enninga EAL, Lucien-Matteoni F, et al. Therapeutic plasma exchange clears circulating soluble PD-L1 and PD-L1-positive extracellular vesicles. *J Immunother Cancer.* 2020;8(2).
29. Padmanabhan A, Connelly-Smith L, Aqui N, et al. Guidelines on the Use of Therapeutic Apheresis in Clinical Practice - Evidence-Based Approach from the Writing Committee of the American Society for Apheresis: The Eighth Special Issue. *J Clin Apher.* 2019;34(3):171-354.
30. Gomes J, Lucien F, Cooper TT, et al. Analytical Considerations in Nanoscale Flow Cytometry of Extracellular Vesicles to Achieve Data Linearity. *Thromb Haemost.* 2018;118(9):1612-1624

For peer review only - http://bmjopen.bmj.com/site/about/guidelines.xhtml

ACKNOWLEDGMENTS

ReCIPE-M1 is supported by grants from the Lawrence W. and Marilyn W. Matteson Fund at Mayo Clinic and R21 CA259236-01 from the National Cancer Institute. Some images were created with BioRender.

DECLARATION OF INTEREST STATEMENT

Intellectual property has been filed addressing discoveries disclosed in this manuscript. The authors report no other relevant conflicts of interest.

For peer review only

For peer review only - http://bmjopen.bmj.com/site/about/guidelines.xhtml

TABLES

Table 1.

INCLUSION CRITERIA	
**Inclusion Criteria**	**Exclusion Criteria**
≥18 years<br><br>Histologic melanoma progressing despite ICI<br><br>Measurable disease<br><br>ECOG ≤ 3<br><br>Ability to consent	Biotin supplement use<br><br>Pregnant or nursing

ARM ASSIGNMENTS	
**Intervention Arm**	sPD-L1 ≥1.7 ng/ml *AND* available clinical capacity
**Standard of Care Arm**	sPD-L1 <1.7 ng/ml *OR* unavailable clinical capacity

Table 1. Study inclusion and exclusion criteria. Eligible patients for the study will be consenting adults with histologically confirmed melanoma receiving PD-(L)1 immunotherapy who are referred for stereotactic body radiation therapy (SBRT). Patients who are receiving biotin supplements, are pregnant, or are nursing are excluded. Patients with elevated sPD-L1 on registration will enter the Intervention Arm where clinical capacity is sufficient. Patients with low sPD-L1 or who enter the trial when clinical capacity is not available will enter the Standard of Care Arm.

For peer review only - http://bmjopen.bmj.com/site/about/guidelines.xhtml

FIGURE LEGENDS

Figure 1. Extracellular PD-L1 Outcompetes Immune Checkpoint Inhibitors to Block Anti-tumor Immunity. (A) While PD-(L)1 inhibitors prevent tumor cell downregulation of T cells, tumor cells secrete evPD-L1 and sPD-L1 (either cleaved by ADAM10/ADAM17 or as splice variants) that outcompete PD-(L)1 inhibitors and downregulate systemic anti-cancer immunity. **(B)** Therapeutic plasma exchange clears extracellular PD-L1 and may re-sensitize tumors to immune checkpoint inhibitor therapy.

Figure 2. Study design in two arms. In brief, eligible patients undergo a registration blood draw and vascular access evaluation to determine appropriate study arm. Patients with elevated baseline sPD-L1 (≥1.7 ng/ml) will enter the Intervention Arm if there is adequate clinical capacity. Other patients will enter the Standard of Care Arm.

For peer review only

For peer review only - http://bmjopen.bmj.com/site/about/guidelines.xhtml

AUTHOR STATEMENT

TD wrote the manuscript. JJO developed hypotheses and concepts, performed initial studies, generated figures and helped write the manuscript. NF performed statistical design. HD, SSP and JLW provided mentorship, equipment, facilities, edits and funding. FL and SM provided additional logistical input, planning and edits. Sponsors and funders have had and will have NO role in study design; collection, management, analysis, or interpretation of data; writing of any report; nor the decision to submit any report for publication. Data will be shared on publication in a peer-reviewed journal without the aid of professional writers. One or more figures was produced using BioRender®.

BIOLOGICAL SPECIMENS

Laboratory specimens (blood draws) will be processed for plasma and peripheral blood mononuclear cells (PBMCs). They will be stored in study identity-labeled tubes at -80C until analysis by flow cytometry, ELISA, or other means for future studies.

DATA SHARING STATEMENT

Deidentified patient information will be made available in a public data repository on publication.

ETHICAL APPROVAL STATEMENT

Study materials and procedures were approved by the Mayo Clinic IRB (approval #19-008055).

For peer review only - http://bmjopen.bmj.com/site/about/guidelines.xhtml

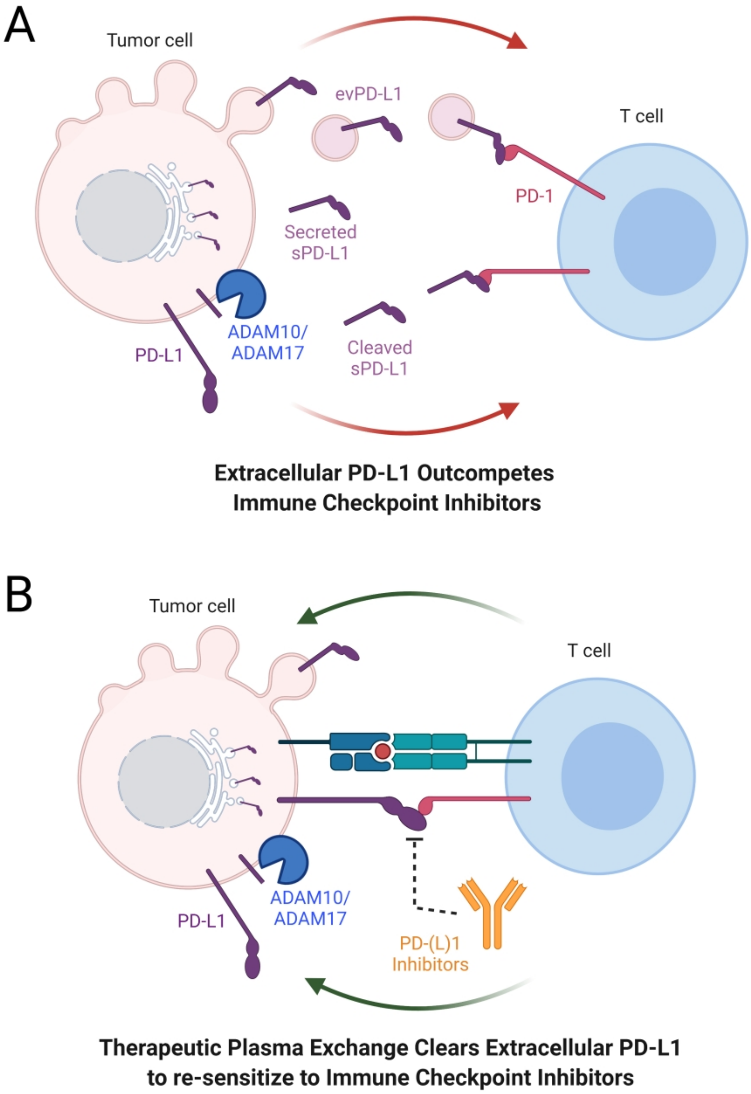

Figure 1. Extracellular PD-L1 Outcompetes Immune Checkpoint Inhibitors to Block Anti-tumor Immunity. (A) While PD-(L)1 inhibitors prevent tumor cell downregulation of T cells, tumor cells secrete evPD-L1 and sPD-L1 (either cleaved by ADAM10/ADAM17 or as splice variants) that outcompete PD-(L)1 inhibitors and downregulate systemic anti-cancer immunity. (B) Therapeutic plasma exchange clears extracellular PD-L1 and may re-sensitize tumors to immune checkpoint inhibitor therapy.

254x354mm (300 x 300 DPI)

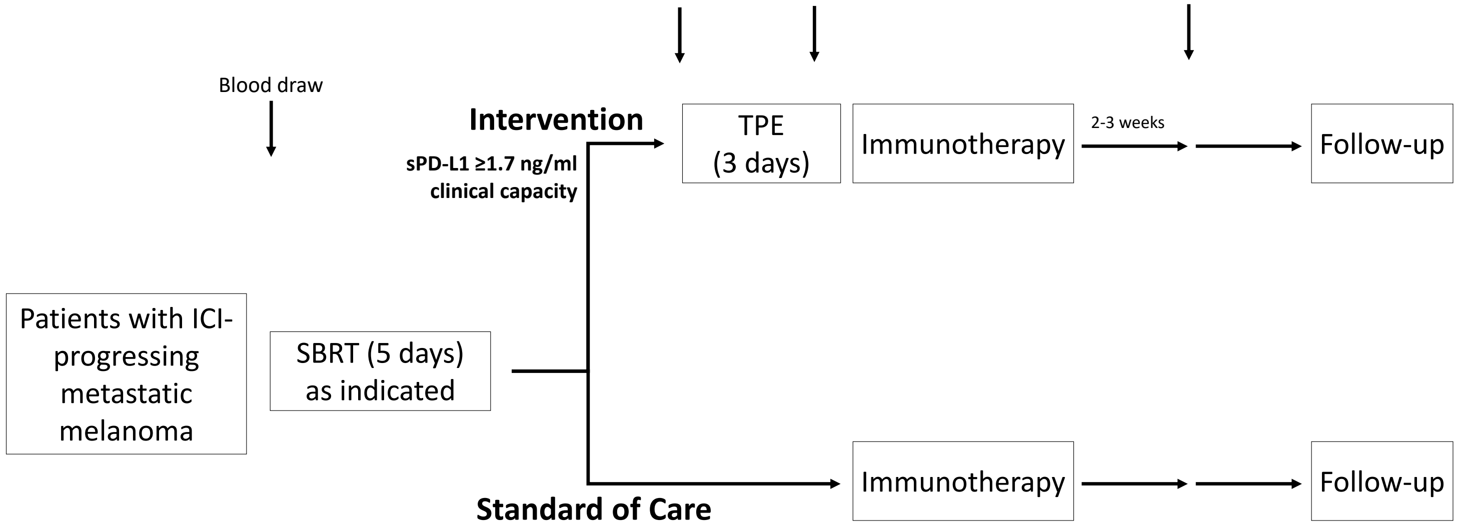
Blood draw
Blood draw
Blood draw
Blood draw
Intervention
sPD-L1 ≥1.7 ng/ml
clinical capacity
TPE
(3 days)
Immunotherapy
2-3 weeks
Follow-up
Patients with ICI-progressing metastatic melanoma
SBRT (5 days) as indicated
Immunotherapy
Follow-up
Standard of Care

1 **SPIRIT 2013 REGISTRATION DATA SET**

Data category	Information
Primary registry and trial identifying number	ClinicalTrials.gov NCT04581382
Date of registration in primary registry	October 9, 2020
Secondary identifying numbers	MC200703 v2
Protocol version	Version 2 2/2021
Source(s) of monetary or material support	NCI CA259236 (R21)<br><br>Lawrence W. and Marilyn W. Matteson Award
Primary sponsor	Mayo Clinic
Secondary sponsor(s)	
Contact for public queries	JJO, orme.jacob@mayo.edu
Contact for scientific queries	JJO, orme.jacob@mayo.edu
Public title	Rescuing Cancer Immunity by Plasma Exchange in Metastatic Melanoma (ReCIPE-M1): An open-label safety trial
Scientific title	*Rescuing Cancer Immunity by Plasma Exchange in Metastatic Melanoma (ReCIPE-M1): An open-label safety trial*
Roles and responsibilities	Study Chair: Jacob Orme, M.D., Ph.D<br><br>Study Co-Chairs: Sean Park, M.D., Ph.D., MS, Jeffrey Winters, M.D., Haidong Dong, M.D., Ph.D., Fabrice Lucien, Ph.D.; Svetomir Markovic, M.D., Ph.D., Matthew Block, M.D., Ph.D., Robert McWilliams, M.D., Lisa Kottschade, APRN, C.N.P.<br><br>Statistician: Nathan Foster, MS

Data category	Information
	Protocol manuscript author: Tara Davidson, MD<br><br>TD wrote the manuscript. JJO developed hypotheses and concepts, performed initial studies, generated figures, and helped write the manuscript. SSP and JLW provided mentorship, equipment, facilities, edits, and funding. FL and SM provided additional logistical input, planning, and edits.<br><br>Approved by the Mayo Clinic Institutional Review Board (IRB). Mayo Clinic independent data monitoring committee (IDMC) manages data in secured database<br><br>Funding source had no role in the design of this study and will not have any role during its execution, analyses, interpretation of the data, or decision to submit results.<br><br>Principal investigator and research physician<br><br>• Preparation of protocol and revisions<br>• Preparation of investigators brochure (IB) and CRFs [case report forms]<br>• Organizing steering committee meetings<br>• Publication of study reports<br>• Study planning<br>• Serious Adverse Events Reporting<br>• Responsible for trial master file<br>• Budget administration<br>• Assistance with international review, board/independent ethics committee applications<br>• Data verification<br>• Organization of serum sample collection<br>• Responsible for trial master file<br><br>Steering committee (SC) - see title page for members<br><br>• Agreement of final protocol<br>• Recruitment of patients and liaising with principle [sic] investigator<br>• Reviewing progress of study and if necessary agreeing changes to the protocol and/or investigators brochure to facilitate the smooth running of the study.

Data category	Information
	Data manager<br>• Maintenance of trial IT system and data entry<br>• Data verification
Countries of recruitment	USA
Health condition(s) or problem(s) studied	Metastatic melanoma
Intervention(s)<br>Pg 8 Line 14-17	Intervention Arm: SBRT (stereotactic body radiation therapy), TPE (therapeutic plasma exchange), ICI (immunotherapy)
	Standard of Care Arm: SBRT (stereotactic body radiation therapy), ICI (immunotherapy)
Key inclusion and exclusion criteria<br>Pg 8 Line 4-13	Ages eligible for study: ≥18 years<br>Sexes eligible for study: both<br>Accepts healthy volunteers: no
	Inclusion criteria: adult patient (≥ 18 years), metastatic melanoma, measurable disease per RECIST criteria, ECOG 3 or better
	Exclusion criteria: pregnancy or nursing, consumption of biotin supplements
Study type<br>Pg 7 Line 20	Interventional
	Allocation: selection by presentation criteria
	Primary purpose: treatment
	Phase I
Date of first enrolment	December 2020
Target sample size<br>Pg 8 Line 7-8	60

Data category	Information
Recruitment status<br><br>Pg 8 Line 7-8	Recruiting<br><br>Listing the trial as a treatment option for eligible patients. Mayo Clinic sees more than 300 patients with metastatic melanoma each year. Melanoma providers are made aware of this trial by research coordinators. 60 patients will be screened from December 2020 through October 2023 with an accrual goal of 20 patients to the TPE Intervention Arm. The principal investigator and study statistician will review the study at least monthly to identify accrual problems.
Primary outcome(s)<br><br>Pg 7 Line 20-21	Safety (adverse events at 1 and 6 months)
Key secondary outcomes<br><br>Pg 7 Line 20-22 and Pg 8 Line 1-2	Kinetics of evPD-L1, sPD-L1, and related immunosuppressive metabolites immediately and 2-3 weeks after TPE<br><br>Relative response of patients to combined therapy through response rate<br><br>Overall survival<br><br>Progression-free survival.
Data collection methods and management	Data will be maintained on a secure database with de-identified patient information. Data will be shared on publication in a peer-reviewed journal without the aid of professional writers.<br><br>Once a participant is enrolled, the study site will make every reasonable effort to follow the participant for the entire study period. It is projected that the rate of loss-to-follow-up on an annual basis will be at most 5%. Study site staff are responsible for developing and implementing local standard operating procedures to achieve this level of follow-up.<br><br>Participants may withdraw from the study for any reason at any

Data category	Information
	time. Participants also may be withdrawn if the study sponsor or government or regulatory authorities terminate the study prior to its planned end date.
Statistical methods<br>Pg 11 Line 12-14<br>Pg 12 Section 2.5	Wilcoxon Signed-Rank test will be used to assess changes in sPD-L1 and evPD-L1 levels over time across the different timepoints of interest.<br><br>For this correlative analysis, we will determine the effects of plasma exchange on immune cell function, observe the kinetics of extracellular vesicles (EVs) after plasma exchange in patients with melanoma, and associate the kinetics with clinical outcome data (RR, OS, PFS). Associations of categorical data will be assessed using Fisher exact tests. Associations of continuous data with binary data will be assessed using standard Wilcoxon Rank-Sum tests. Assessment of the change in continuous data over time will be done using the Wilcoxon Signed-Rank test. Kaplan-Meier methods and the log-rank test will be used for time-to-event data. This translational study is considered exploratory and hypothesis generating due to the small proposed sample size for this study.

Data category	Information
Ethics and Dissemination<br><br>Pg 12 Line 10-22, Pg 13 Line 1-9	Any modifications to the protocol which may impact on the conduct of the study, potential benefit of the patient or may affect patient safety, including changes of study objectives, study design, patient population, sample sizes, study procedures, or significant administrative aspects will require a formal amendment to the protocol. Such amendment will be approved by the Mayo Clinic IRB prior to implementation and notified to the health authorities in accordance with local regulations.<br><br>Administrative changes of the protocol are minor corrections and/or clarifications that have no effect on the way the study is to be conducted. These administrative changes will be documented in a memorandum. The Ethics Committee/IRB may be notified of administrative changes at the discretion of the study chair and co-chairs.
Ancillary and post-trial care	Patients that are enrolled into the study are covered by indemnity for negligent harm through the standard NHS [*National Health Service*] Indemnity arrangements.

For peer review only - http://bmjopen.bmj.com/site/about/guidelines.xhtml

Data category	Information
Amendments to the protocol	Protocol amended 3/2021 to allow central access for patients without patent peripheral blood access.
Data Monitoring	The Mayo Clinic Cancer Center (MCCC) Data Safety Monitoring Board (DSMB) is responsible for reviewing accrual and safety data for this trial at least twice a year, based on reports provided by the MCCC Statistical Office. Any safety issues requiring protocol changes are communicated through protocol amendments.<br><br>Adverse Event Stopping Rule: Based on previous experience with this disease, we expect approximately 10% of patients to experience Grade 4+ adverse events. If at any time, 3 of the first 12 patients have experienced any Grade 4 or 5 adverse event (at least possibly related to the study treatment), accrual to the study will be suspended to allow for a full review of the data. Each grade 5 event will be reviewed on a case by case basis in a real time fashion to determine whether study accrual should be suspended. After consideration by the study team [ie, Study Chair(s), Statistician, Operations Office, etc] and consultation with representatives at the primary Internal Review Board (IRB) affiliated with the Operations Office, a decision will be made as to whether and how the study will proceed.

Data category	Information
Model Informed Consent	See attached

For peer review only
