# Supplementary material for: Rescuing Cancer Immunity by Plasma Exchange in Metastatic Melanoma (ReCIPE-M1): protocol for a single-institution, open-label safety trial of plasma exchange to clear sPD-L1 for immunotherapy
Source: BMJ Open. 2022 May 11;12(5):e050112. doi: 10.1136/bmjopen-2021-050112 (PMC9109028; doi:10.1136/bmjopen-2021-050112)
Supplement: Reviewer comments [file bmjopen-2021-050112.reviewer_comments.pdf]

PEER REVIEW HISTORY

BMJ Open publishes all reviews undertaken for accepted manuscripts. Reviewers are asked to complete a checklist review form (**http://bmjopen.bmj.com/site/about/resources/checklist.pdf**) and are provided with free text boxes to elaborate on their assessment. These free text comments are reproduced below.

ARTICLE DETAILS

**TITLE (PROVISIONAL)**	Rescuing Cancer Immunity by Plasma Exchange in Metastatic Melanoma (ReCIPE-M1): Protocol for a single-institution, open-label safety trial of plasma exchange to clear sPD-L1 for immunotherapy
**AUTHORS**	Davidson, Tara M.; Foster, Nathan; Lucien, Fabrice; Svetomir, Markovic; Dong, Haidong; Winters, Jeffrey L.; Park, Sean S.; Orme, Jacob

VERSION 1 – REVIEW

**REVIEWER**	Umang Swami<br>University of Utah Health Huntsman Cancer Institute
**REVIEW RETURNED**	18-Apr-2021

**GENERAL COMMENTS**	It was a pleasure reviewing the manuscript "Rescuing Cancer Immunity by Plasma Exchange in Metastatic Melanoma (ReCIPE-M1): An open-label safety trial"<br><br>The authors present their proposed clinical trial. Manuscript is well written. I have few suggestions:-<br><br>1. In section 2.2 second and third sentence need to be re-written to correct grammatical errors<br><br>2. The manuscript mentions these patients are PD-1 refractory while figure 2 and protocol eligibility criteria does not mention this.<br><br>3. The authors need to explain more regarding radiation therapy as standard of care. It is not standard of care to give SBRT to all oligo-progressive lesions.<br><br>4. I believe 60 patients will be screened not accrued on the trial.<br><br>5. Why the standard of care arm will continue only checkpoint and not escalation to ipilimumab with nivolumab or BRAF targeted therapy? What is the rationale of continuing the same therapy on which they have progressed and missing the window of opportunity to treat them aggressively?

**REVIEWER**	Shalini Tanwar<br>NHLBI, Lab of Molecular Immunology
**REVIEW RETURNED**	04-May-2021

**GENERAL COMMENTS**	The study proposal is precise and succinct and appropriate to be accepted.<br>The study schema flow chart is great and self explanatory. However, section 2.3 'Study interventions' need similar clarity in the number

	and timing of blood draws from enrollees.<br><br>Figure 1 cartoon needs some work at being self explanatory. Showing parallel scenarios with or without the presence of sPD-L1 and evPD-L1 may help.

**REVIEWER**	Mike Bradburn<br>University of Sheffield, ScHARR
**REVIEW RETURNED**	15-Dec-2021

**GENERAL COMMENTS**	The manuscript describes a protocol for a non-randomised trial to evaluate therapeutic plasma exchange (TPE) in addition to radiotherapy and immunotherapy among patients with metastatic melanoma. Patients with a compromised immune response (here characterised by sPD-L1 >= 1.7ng/ml) will receive TPE as an add-on to their treatment pathway whilst those with lower levels will receive usual care without TPE. The trial aims to recruit 60 people of whom 20 are expected to meet the criteria for TPE.<br><br>The study has been approved and registered (NCT04581382; the manuscript appears consistent with this) and recruitment commenced; the trial will continue into 2023. Whilst this review does not attempt to modify this in any way, I feel some elements of the study could be better described here. My review is that of a statistician/methodologist rather than an oncologist or immunotherapist - as may be evident by my attempt to define the sPD-L1 marker.<br><br>I. The SPIRIT checklist (page 24 of the document) is incomplete compared to the version on the EQUATOR website.<br>https://www.equator-network.org/reporting-guidelines/spirit-2013-statement-defining-standard-protocol-items-for-clinical-trials/<br>Most items are nevertheless covered in the manuscript, but ones which I believe need more detail are<br>3: Protocol version (the attached clinical protocol is February 2021 but this is not in the main body of the manuscript)<br>5d/21a Composition and role of IDMC<br>15/18b: Strategies to achieve enrollment and follow up<br>19: data management<br>20a,20c,21b: Statistical methods<br>25: Amendments to protocol<br>30: Ancillary/Post trial care<br>32: Model consent form<br><br>Some of these are described further below<br><br>II. Please clarify the order in which participants consent and are tested for sPD-L1. Do participants consent to treatment before or after their sPD-L1 status is known? I assume before but please clarify.<br><br>III. It would also help to describe the importance of having a control group (sPD-L1 <1.7) - presumably this is to assess whether harms may be attributable to the addition of TPE over and above those of radiotherapy/immunotherapy, and to assess the feasibility of a future RCT?<br><br>IV. The comment "at least one metastatic lesion will not be radiated to assess response" - I assume this is acceptable from an ethical perspective but want to flag this. At face value it's not obvious why

	radiation would be withheld for the purpose of measurements (but this is likely my lack of awareness of PERCIST).<br><br>V. The protocol describes statistical methods for analysing the data and stopping rules/considerations for harms, neither of which are included in the main text. Why were these omitted? They seem important. Will participants be analysed based on their actual treatment (TPE+) rather than sPD-L1 group? I assume so but please say. Also, no classification of adverse events is given (MedDRA? AEs of specific interest?) Please add this.<br><br>VI. A more minor note, the Clinical Trials registry describes TPE as "a way to 'clean' or 'flush out' the blood", which could be a useful addition here, given the generalist readership of BMJ Open.

VERSION 1 – AUTHOR RESPONSE

Reviewer: 1

1. In section 2.2 second and third sentence need to be re-written to correct grammatical errors

This has been revised to correct grammatical errors, thank you.

2. The manuscript mentions these patients are PD-1 refractory while figure 2 and protocol eligibility criteria does not mention this.

Thank you. This has been added on ClinicalTrials.org (submitted 12/21/2021) and updated in both Table 1 and Figure 2.

3. The authors need to explain more regarding radiation therapy as standard of care. It is not standard of care to give SBRT to all oligo-progressive lesions.

We have rephrased our wording and excluded "standard of care". Preferential targets for radiotherapy will be symptomatic lesions or lesions that can cause pain, fracture, or other symptoms in the near future.

4. I believe 60 patients will be screened not accrued on the trial.

Thank you. This has been clarified in the manuscript.

5. Why the standard of care arm will continue only checkpoint and not escalation to ipilimumab with nivolumab or BRAF targeted therapy? What is the rationale of continuing the same therapy on which they have progressed and missing the window of opportunity to treat them aggressively?

Thank you. This has been clarified in the manuscript. Escalation to ipilimumab and nivolumab as well as BRAF-targeted therapy is allowed on the trial per clinician preference.

Reviewer: 2

1. The study schema flow chart is great and self explanatory. However, section 2.3 'Study interventions' need similar clarity in the number and timing of blood draws from enrollees.

Thank you very much. We have updated this section to better reflect the particulars of the study.

2. Figure 1 cartoon needs some work at being self explanatory. Showing parallel scenarios with or without the presence of sPD-L1 and evPD-L1 may help.

Thank you. As you have suggested, we split Figure 1 into parallel scenarios with or without the presence of sPD-L1 and evPD-L1. This has also been updated in the manuscript.

Reviewer: 3

1. I. The SPIRIT checklist (page 24 of the document) is incomplete compared to the version on the EQUATOR website. https://www.equator-network.org/reporting-guidelines/spirit-2013-statement-defining-standard-protocol-items-for-clinical-trials/ Most items are nevertheless covered in the manuscript, but ones which I believe need more detail are
   3: Protocol version (the attached clinical protocol is February 2021 but this is not in the main body of the manuscript) 5d/21a Composition and role of IDMC
   15/18b: Strategies to achieve enrollment and follow up
   19: data management
   20a,20c,21b: Statistical methods
   25: Amendments to protocol
   30: Ancillary/Post trial care
   32: Model consent form

This has been updated, thank you.

2. Please clarify the order in which participants consent and are tested for sPD-L1. Do participants consent to treatment before or after their sPD-L1 status is known? I assume before but please clarify.

Thank you. This has been clarified in the manuscript.

3. It would also help to describe the importance of having a control group (sPD-L1 <1.7) - presumably this is to assess whether harms may be attributable to the addition of TPE over and above those of radiotherapy/immunotherapy, and to assess the feasibility of a future RCT?

Thank you. That is exactly the reasoning behind the control group. This has been clarified in the manuscript.

4. The comment "at least one metastatic lesion will not be radiated to assess response" - I assume this is acceptable from an ethical perspective but want to flag this. At face value it's not obvious why radiation would be withheld for the purpose of measurements (but this is likely my lack of awareness of PERCIST).

Thank you. Patients with oligometastases (<=3 lesions) are treated "definitively" with SBRT and therefore are not referred for enrollment to our study. As the goal of our study is to improve immunotherapy response, patients with widespread metastases (>3 lesions) are referred to radiation for inclusion of this study. Therefore, having at least one non-irradiated lesion in this population with widespread metastases to allow treatment efficacy was approved by our institutional IRB. Furthermore, if the non-irradiated lesion is the only metastasis that persists after our therapy protocol, SBRT can be delivered on subsequent follow up. This is a key point and we have tried to clarify this further in the manuscript.

5. The protocol describes statistical methods for analysing the data and stopping rules/considerations for harms, neither of which are included in the main text. Why were these omitted? They seem important. Will participants be analysed based on their actual treatment (TPE+) rather than sPD-L1 group? I assume so but please say. Also, no classification of adverse events is given (MedDRA? AEs of specific interest?) Please add this.

Thank you, we have added these stopping rules and statistical methods to the text. Participants will be analyzed on their treatment, which we have also clarified. The CTCAE 5.0 terms are used in the trial, which we have added to the manuscript. Thank you very much, as we believe this significantly improves the manuscripts utility.

6. A more minor note, the Clinical Trials registry describes TPE as "a way to 'clean' or 'flush out' the blood", which could be a useful addition here, given the generalist readership of BMJ Open.

Thank you very much, we have added this phrasing to help the readership.

VERSION 2 – REVIEW

**REVIEWER**	Umang Swami<br>University of Utah Health Huntsman Cancer Institute
**REVIEW RETURNED**	07-Jan-2022

**GENERAL COMMENTS**	The authors have replied to all my queries. I do not have any additional questions.

**REVIEWER**	Mike Bradburn<br>University of Sheffield, ScHARR
**REVIEW RETURNED**	28-Dec-2021

**GENERAL COMMENTS**	The manuscript has addressed most of the previous comments but I believe has not done so completely. I've listed these below ordered by the original comment<br><br>Editor point 6: I didn't see a copy of the patient consent form in the resubmission.<br><br>Reviewer 1 point 2: Table 1 and figure 2 have been updated to include "progressing" but the clinicaltrials.gov entry (https://clinicaltrials.gov/ct2/show/record/NCT04581382) has not been updated as of 28 Dec2021. This isn't a problem with the manuscript per se but I can't verify this.<br><br>Reviewer 3 point 1: the SPIRIT checklist provided isn't exactly the same as the current version on the Equator website (https://www.equator-network.org/reporting-guidelines/spirit-2013-statement-defining-standard-protocol-items-for-clinical-trials/) Most of the points are there nonetheless, but the following still appear to be missing:<br><br>-5d Composition, roles, and responsibilities of the coordinating centre, steering committee, endpoint adjudication committee, data management team, and other individuals or groups overseeing the trial, if applicable<br><br>-15 Strategies for achieving adequate participant enrolment to reach target sample size<br><br>-18b: Plans to promote participant retention and complete follow-up, including list of any outcome data to be collected for participants who discontinue or deviate from intervention protocols<br><br>-20a Statistical methods for analysing primary and secondary outcomes. Reference to where other details of the statistical analysis plan can be found, if not in the protocol [see also below]<br><br>-21a Composition and role of IDMC

	Reviewer 3 point 3 : I accept the trial is largely exploratory and won't be based on formal statistical inference, but some more details on the statistical methods would help. this could be as simple as transcribing some of the detail from section 16.3.4 of the main protocol.

VERSION 2 – AUTHOR RESPONSE

Reviewer: 3

1. Editor point 6: I didn't see a copy of the patient consent form in the resubmission.
***Comment from the Editor: Thank you for supplying this as requested - we can see that you supplied it on the system.

Thank you very much for supplying.

2. Reviewer 1 point 2: "The manuscript mentions these patients are PD-1 refractory while figure 2 and protocol eligibility criteria does not mention this." Table 1 and figure 2 have been updated to include "progressing" but the clinicaltrials.gov entry (https://clinicaltrials.gov/ct2/show/record/NCT04581382) has not been updated as of 28 Dec2021. This isn't a problem with the manuscript per se but I can't verify this.

Thank you very much, we contacted our liaisons with ClinicalTrials.gov and have re-submitted these changes 2/23/2022. Thank you very much!

3. Reviewer 3 point 1: the SPIRIT checklist provided isn't exactly the same as the current version on the Equator website (https://www.equator-network.org/reporting-guidelines/spirit-2013-statement-defining-standard-protocol-items-for-clinical-trials/) Most of the points are there nonetheless, but the following still appear to be missing:
-5d Composition, roles, and responsibilities of the coordinating centwe, steering committee, endpoint adjudication committee, data management team, and other individuals or groups overseeing the trial, if applicable

-15 Strategies for achieving adequate participant enrolment to reach target sample size

-18b: Plans to promote participant retention and complete follow-up, including list of any outcome data to be collected for participants who discontinue or deviate from intervention protocols

-20a Statistical methods for analyzing primary and secondary outcomes. Reference to where other details of the statistical analysis plan can be found, if not in the protocol [see also below]

-21a Composition and role of IDMC

Thank you very much, we have updated the SPIRIT checklist as requested.

4. Reviewer 3 point 3 : I accept the trial is largely exploratory and won't be based on formal statistical inference, but some more details on the statistical methods would help. this could be as simple as transcribing some of the detail from section 16.3.4 of the main protocol.

Thank you for this excellent suggestion. The below paragraph has been added to the manuscript as section 2.6 Statistical Methods:

"For this correlative analysis, we will determine the effects of plasma exchange on immune cell function, observe the kinetics of extracellular vesicles (EVs) after plasma exchange in patients with melanoma, and associate the kinetics with clinical outcome data (RR, OS, PFS). Associations of categorical data will be assessed using Fisher exact tests. Associations of continuous data with binary data will be assessed using standard Wilcoxon Rank-Sum tests. Assessment of the change in continuous data over time will be done using the Wilcoxon Signed-Rank test. Kaplan-Meier methods and the log-rank test will be used for time-to-event data. This translational study is considered exploratory and hypothesis generating due to the small proposed sample size for this study."
